# Supplementary material for: CRISPR targeting of mmu-miR-21a through a single adeno-associated virus vector prolongs survival of glioblastoma-bearing mice
Source: Mol Ther. 2024 Nov 19;33(1):133–51. doi: 10.1016/j.ymthe.2024.11.023 (PMC11764731; doi:10.1016/j.ymthe.2024.11.023)
Supplement: Document S1. Figures S1–S8 and Tables S1–S6 [file mmc1.pdf]

YMTHE, Volume *33*

Supplemental Information

CRISPR targeting of mmu-miR-21a through a single adeno-associated virus vector prolongs survival of glioblastoma-bearing mice

Lisa Nieland, Anne B. Vrijmoet, Isabelle W. Jetten, David Rufino-Ramos, Alexandra J.E.M. de Reus, Koen Breyne, Benjamin P. Kleinstiver, Casey A. Maguire, Marike L.D. Broekman, Xandra O. Breakefield, and Erik R. Abels

SUPPLEMENTAL MATERIAL

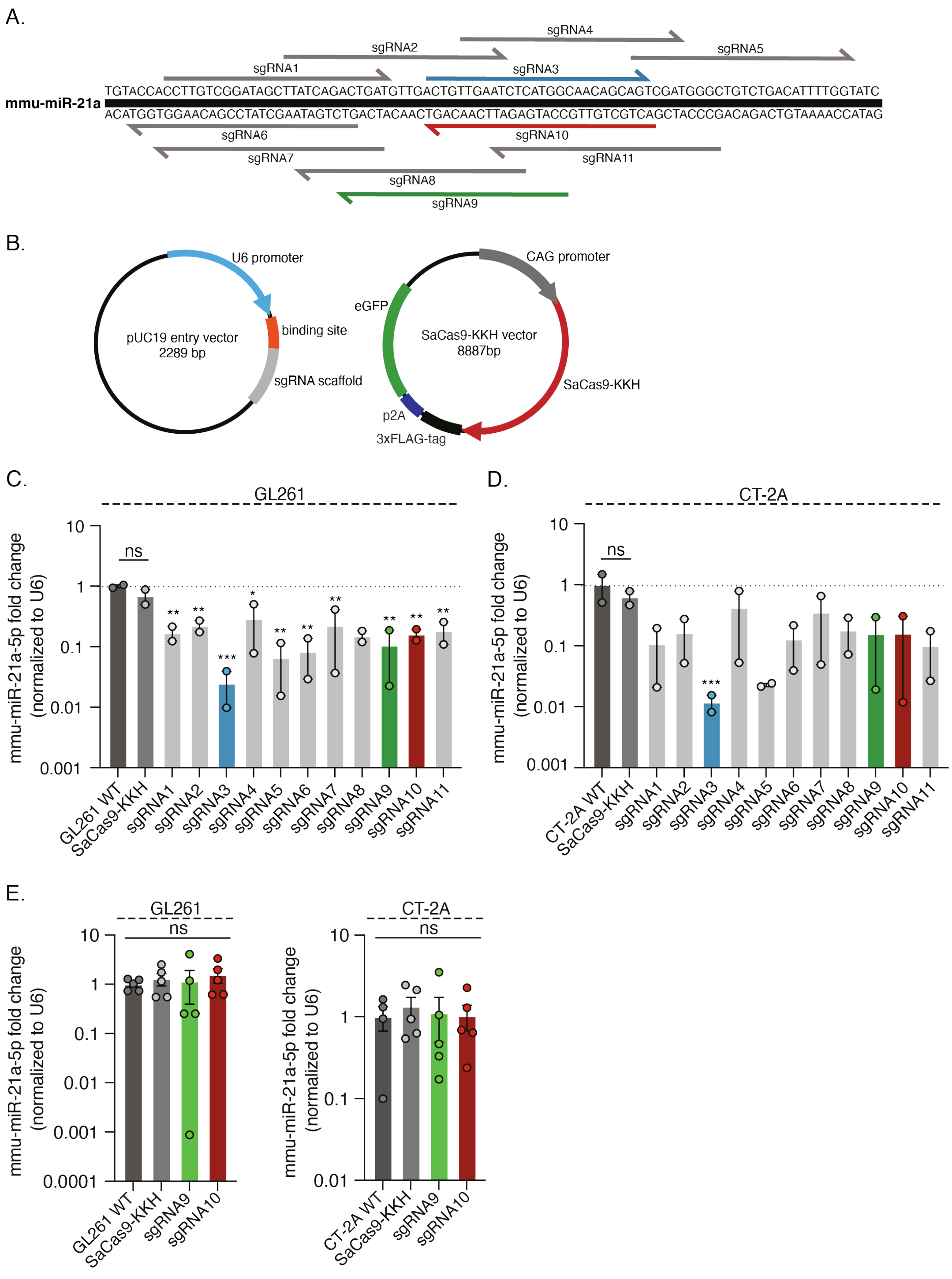

Figure S1. Screening of eleven guides targeting mouse mmu-mir-21a locus. (A) Schematic display of the mmu-mir-21a locus showing the locations of 11 guides at the mmu-miR-21a locus

at different locations showing sgRNA3 highlighted in blue, sgRNA9 in green, and sgRNA10 in red. (B) Plasmid pUC19 (left; size: 2289bp) schematically shows the U6 promoter (blue), binding site (orange), and sgRNA scaffold (grey). Plasmid MSP2588 (right; size: 8887bp) containing the SaCas9-KKH (red) under a CAG (grey) promoter and tagged by both 3xFLAG-tag (black), a peptide 2A (p2A) sequence (blue) and a green fluorescent protein (GFP - green). (C-D) Fold expression levels of mmu-miRa-21-5p targeted by 11 different guides showing 200-fold and 100-fold reduction for sgRNA3 (blue) in both GL261 and CT-2A respectively. And ~10-fold reduction for sgRNA9 (green) and sgRNA10 (red) in both GL261 and CT-2A. Cells co-transfected with SaCas9-KKH expressing plasmid and a single guide were compared to WT representing non-transfected control cell line or the SaCas9-KKH control, referring to cells single transfected with the SaCas9-KKH expressing plasmid. Data represent duplicates and are presented as the mean with SEM (error bars). Multi-comparison (Tukey) one-way ANOVA, $^{**}p < 0.01$, $^{***}p < 0.001$, (ns = not significant). (E) Cell lines (GL261 and CT-2A) co-transfected with sgRNA9 (green) and sgRNA10 (red) did not show a significant change in mmu-miR-21a-5p fold expression compared to WT representing non-transfected control cell line or SaCas9-KKH control, referring to cells single transfected with the or SaCas9-KKH expressing plasmid. Data are presented as the mean with SEM (error bars). Multi-comparison (Tukey) one-way ANOVA, (ns = not significant)

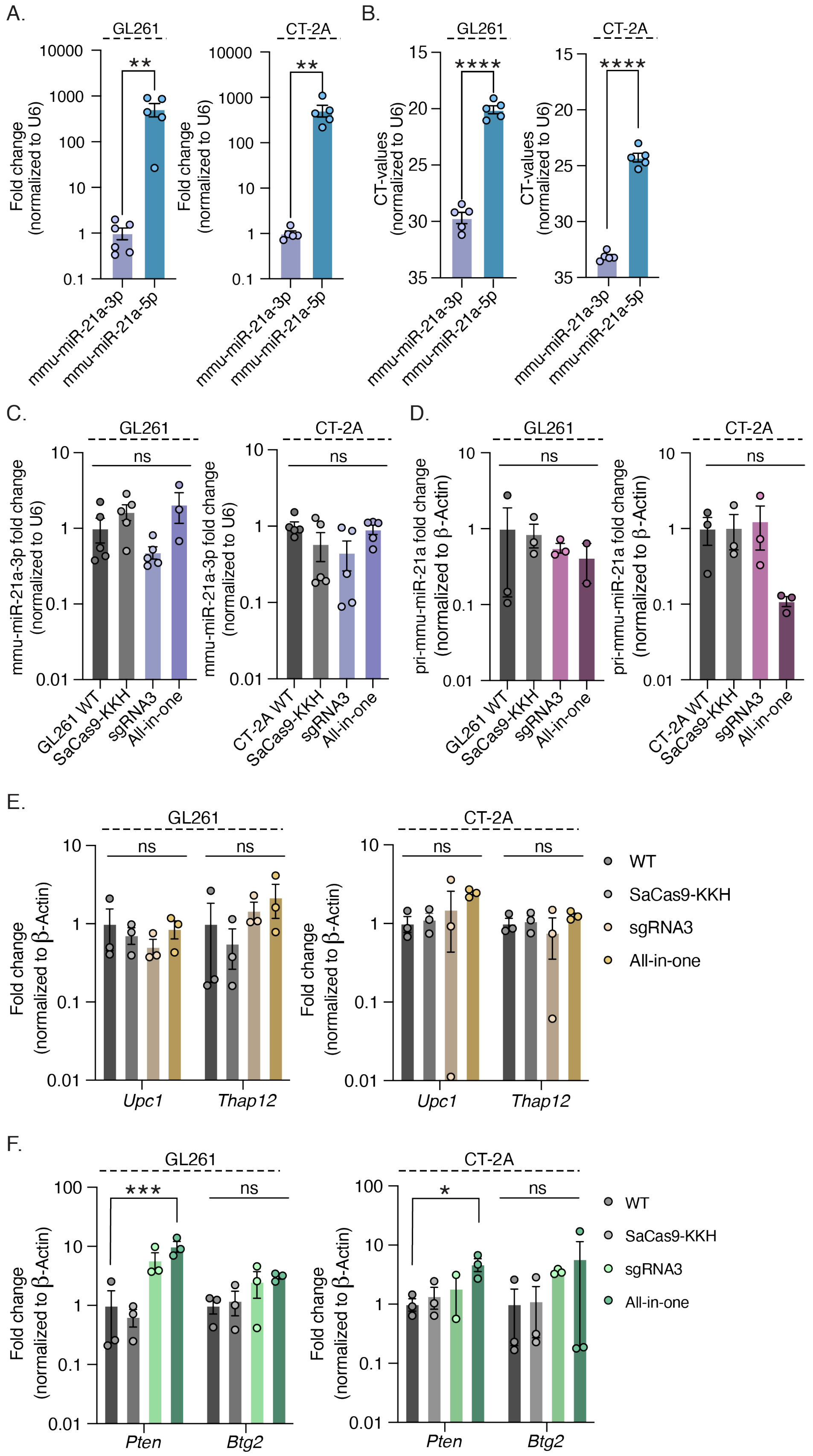

A.
GL261
CT-2A
Fold change (normalized to U6)
mmu-miR-21a-3p
mmu-miR-21a-5p
B.
CT-values (normalized to U6)
C.
mmu-miR-21a-3p fold change (normalized to U6)
GL261 WT
SaCas9-KKH
sgRNA3
All-in-one
CT-2A WT
D.
pri-mmu-miR-21a fold change (normalized to β-Actin)
E.
Fold change (normalized to β-Actin)
Upc1
Thap12
WT
SaCas9-KKH
sgRNA3
All-in-one
F.
Pten
Btg2

Figure S2. Mmu-miR-21a-3p and mmu-miR-21-5p arm analysis. (A) The mmu-miR-21a-5p arm is 516- and 520-fold upregulated compared to the mmu-miR-21a-3p arm in both GL261 and CT-2A wild-type (WT) cells respectively as normalized to the U6 housekeeping gene. Data are presented as the mean with SEM (error bars). Multi-comparison (Tukey) one-way ANOVA, ****p < 0.0001. (B) CT-values normalized to the U6 housekeeping show low expression levels of mmu-miR-21a-3p (~35 CT) compared to mmu-miR-21a-5p (~20 CT) in WT GL261 and CT-2A cell lines. Data are presented as the mean with SEM (error bars). Multi-comparison (Tukey) one-way ANOVA, ****p < 0.0001. (C) Fold expression of mmu-miR-21a-3p showed no differences between edited (sgRNA3 and all-in-one) compared to non-edited (WT and SaCas9-KKH) controls for both cell lines (GL261 and CT-2A). Data are presented as the mean with SEM (error bars). Multi-comparison (Tukey) one-way ANOVA, ns – not significant. (D) Fold expression of mmu-pri-miR-21a shows no differences between edited (sgRNA3 and all-in-one) compared to non-edited (WT and SaCas9-KKH) controls for both cell lines (GL261 and CT-2A). Data are presented as the mean with SEM (error bars). Multi-comparison (Tukey) one-way ANOVA, ns – not significant. (E) Downstream mRNA target of the mmu-miR-21a-5p arm *Pten* was upregulated in all-in-one CRISPR-edited cells compared to WT cells in GL261 and CT-2A cells, respectively. Btg2 was not differently expressed between groups. Data are presented as the mean with SEM (error bars). Multi-comparison (Tukey) one-way ANOVA, *p < 0.05, ***p < 0.001, ns – not significant. (F) Downstream mRNA targets of the mmu-miR-21a-3p arm, *Upc1* and *Thap12* showed no differences in edited cells (sgRNA3 and all-in-one) compared to WT for both GL261 and CT-2A cell lines. Data are presented as the mean with SEM (error bars). Multi-comparison (Tukey) one-way ANOVA, ns – not significant.

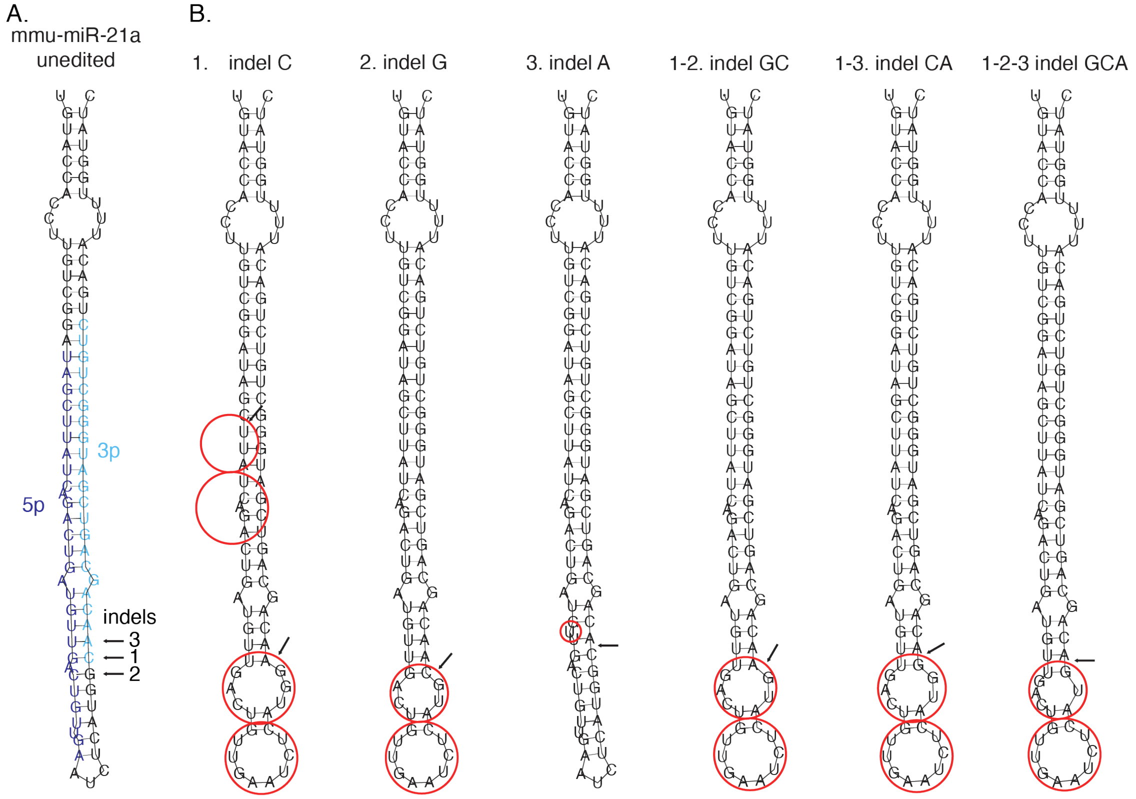

Figure S3. Predicted structures of RNA folding of the pre-mmu-miR-21a hairpin loop. (A) Schematic illustration of the non-edited pre-mmu-miR-21a hairpin loop with the -5p and -3p highlighted in blue. (B) By using RNA-fold (http://rna.tbi.univie.ac.at/cgi-bin/RNAWebSuite/RNAfold.cgi) hairpin loop predicted structures are displayed of RNA folding after deleting one of the three most frequent indels observed: 1) indel "C" (55.5%), 2) indel "G", 3) indel "T" (64.2%), or combinations; 1-2 indel “GC”, 1-3 indel CA; 1-2-3 indel “GCA” (Figure 1E (shown in reverse complement)). The arrows point to the location of the deletion and in red are the altered sequences highlighted compared to the non-edited hairpin loop.

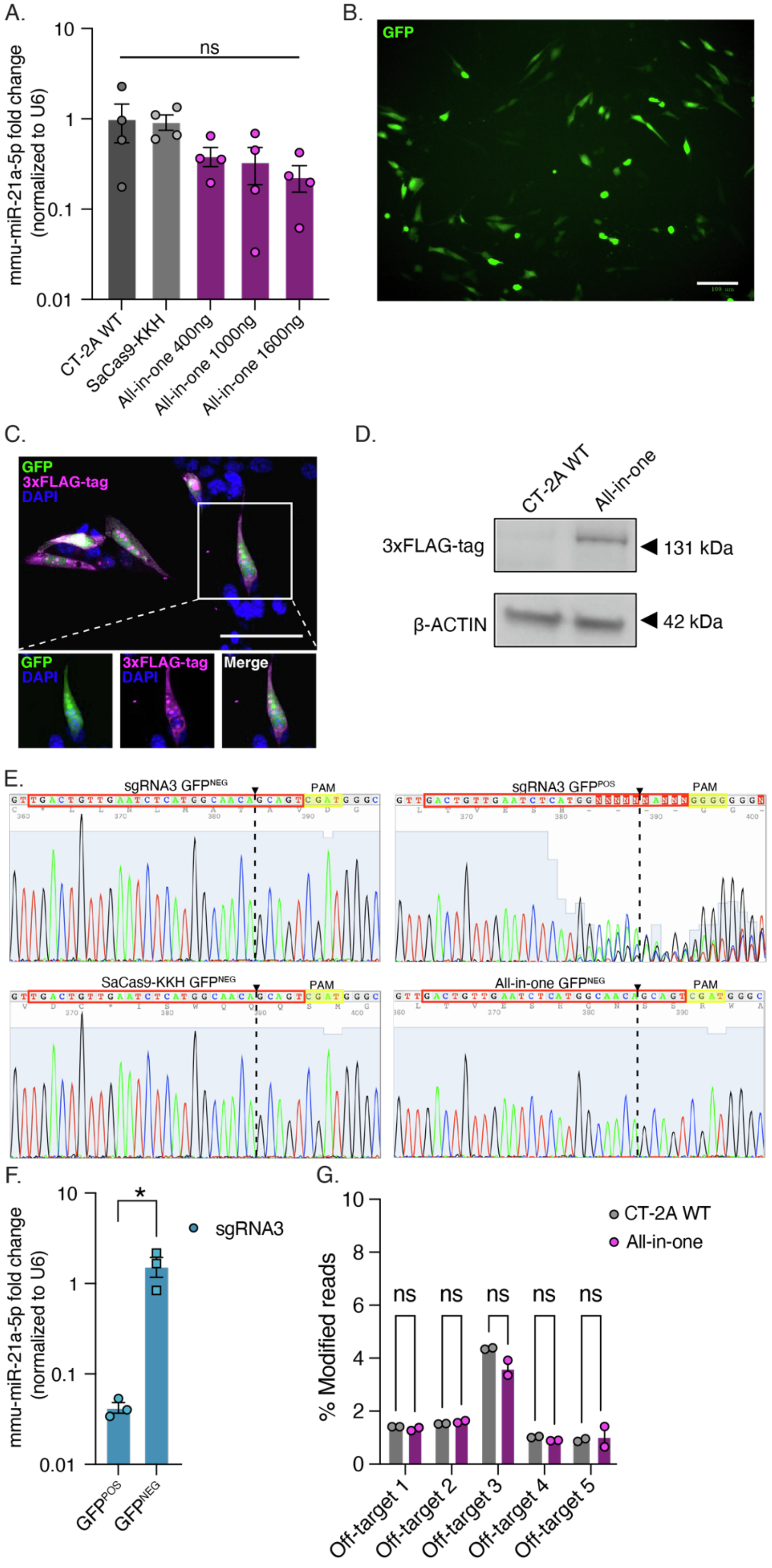
A.
ns
mmu-miR-21a-5p fold change (normalized to U6)
CT-2A WT
SaCas9-KKH
All-in-one 400ng
All-in-one 1000ng
All-in-one 1600ng
B.
GFP
C.
GFP
3xFLAG-tag
DAPI
Merge
D.
CT-2A WT
All-in-one
3xFLAG-tag
131 kDa
β-ACTIN
42 kDa
E.
sgRNA3 GFPNEG
sgRNA3 GFPPOS
SaCas9-KKH GFPNEG
All-in-one GFPNEG
PAM
F.
sgRNA3
GFPPOS
GFPNEG
G.
CT-2A WT
All-in-one
% Modified reads
ns
Off-target 1
Off-target 2
Off-target 3
Off-target 4
Off-target 5

Figure S4. Validation of all-in-one transfection components in tumor cells. (A) Fold expression of mmu-miR-21a-5p levels after transfection with three different concentrations of the all-in-one construct (400ng, 1000ng and 1600ng) targeting mmu-miR-21a-5p. Data represent triplicates and are presented as the mean with SEM (error bars). Multi-comparison one-way ANOVA, ns – not significant. (B) The representative image shows 80% of the cells positive for GFP after a single transfection with the all-in-one plasmid. (Magnification 4x; scale bar 100 μm). (C) Representative images showing GFP^{POS} cells post-transfection with the all-in-one plasmid co-localized with 3xFLAG-tag antibody which is fused to the SaCas9-KKH protein. (Magnification 40x; scale bar 10 μm). (D) Western blot analysis shows the presence of 3xFLAG-tag, GFP and SaCas9-KKH protein in GFP^{POS} sorted cells construct, normalized to β-actin. (E) Sanger sequencing plots show no disruption of the mmu-miR-21a sequence after co-transfection of glioma cells with sgRNA3 and SaCas9-KKH plasmid sorted for GFP^{NEG} but showed an indel for GFP^{POS} sorted cells. Both SaCas9-KKH only GFP^{NEG} and all-in-one GFP^{NEG} showed a non-edited mmu-miR-21a sequence. (F) qRT-PCR shows significantly reduced mmu-miR-21a-5p fold expression of CT-2A cells co-transfected with sgRNA3 and SaCas9-KKH sorted for GFP^{POS} compared to GFP^{NEG} (blue), non-transfected CT-2A WT cells and single-transfected SaCas9-KKH only (grey) GFP^{POS} and GFP^{NEG} control. Data represent triplicates and are presented as the mean with SEM (error bars). Multi-comparison one-way ANOVA, *p<0.05, ****p<0.0001. (G) NGS analysis of the top 5 saCas9-KKH predicted off-targets comparing CT-2A WT non-edited with CRISPR-edited cells of all-in-one GFP^{POS} sorted. No differences in the percentage of modified reads were observed.

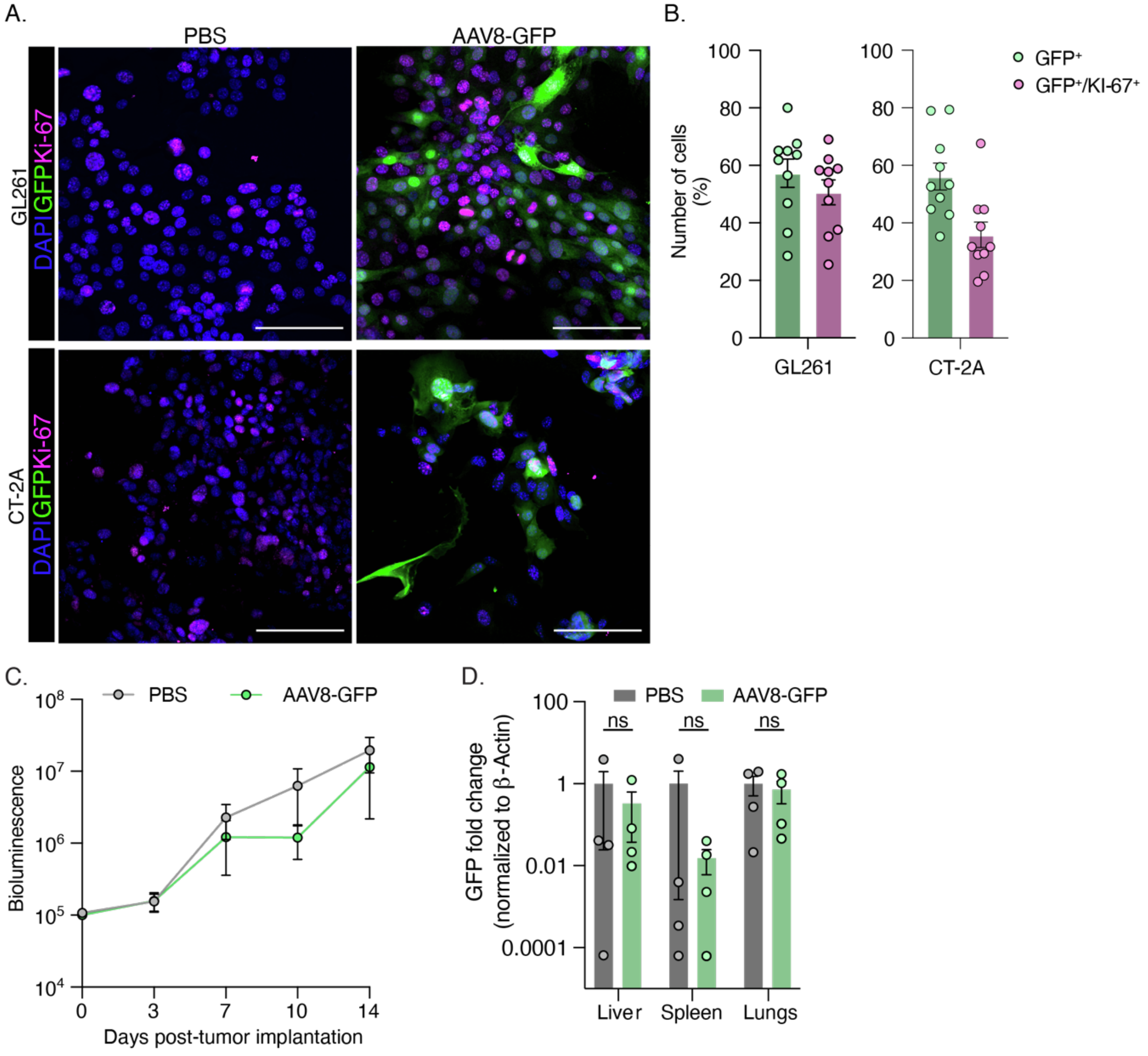

Figure S5. Transduction efficiency of AAV8 in GL261 and CT-2A in culture and orthotopic tumor grafts. (A) Representative images show the transduction profile of AAV8-GFP in glioma cell lines GL261 and CT-2A showing co-localization of GFP^{POS} cells with Ki-67 (proliferation marker) (pink) compared to PBS control. (Magnification 10x; scale bar 100 μm). (B) The number of GFP^{POS} cells as a percentage of total cells based on $DAPI^{POS}$ cells, were quantified showing a ~55% transduction rate in GL261 cells and CT-2A cells *in vitro*. Co-localization of and $GFP^{POS}Ki$-67^{POS} cells were quantified as a percentage of total cells ($DAPI^{POS}$) and showed a ~50% and ~35% in both cell lines, respectively. (C) Bioluminescence levels of mice implanted with CT-2A cells

and i.c. injected with AAV8-GFP (green) or PBS (grey) at day 7 post-tumor injection showing tumor growth, as measured by IVIS and over 14 days, did not significantly differ between mice injected with PBS (n=6) or AAV8-GFP (n=6). Data are presented as the mean with SEM (error bars). Using Šídák's multiple comparisons two-way ANOVA, showed no significant differences between groups. (D) qRT-PCR measuring GFP levels in the lungs and spleen showed no significant differences between AAV8-GFP (green) and PBS control (grey). In the liver, significant higher expression of GFP was observed in AAV8-GFP. Data are presented as four replicates and show the mean with SEM (error bars). Šídák's multiple comparisons two-way ANOVA, no significant differences between groups were observed.

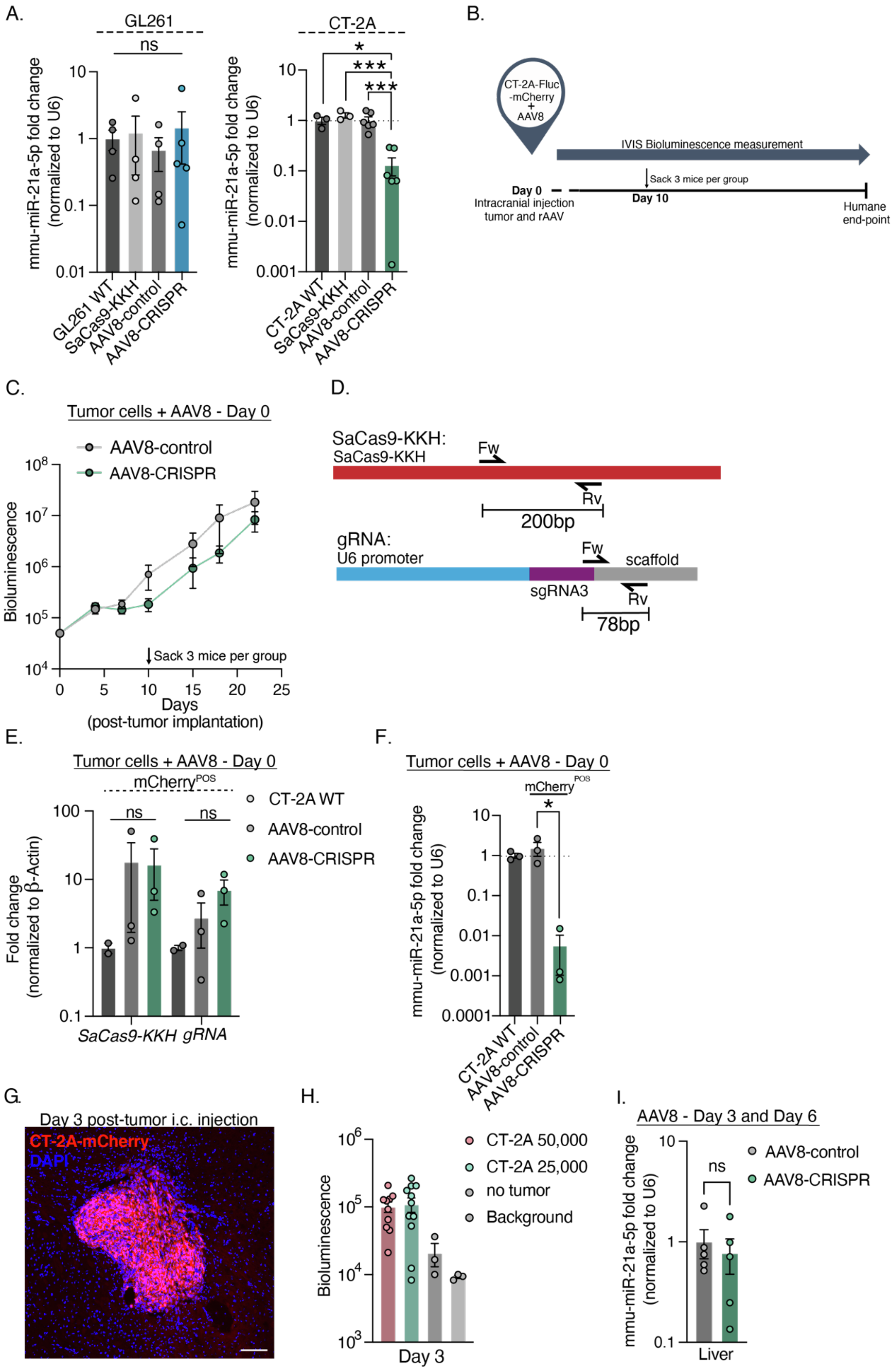
A.
GL261
ns
mmu-miR-21a-5p fold change (normalized to U6)
10
1
0.1
0.01
GL261 WT
SaCas9-KKH
AAV8-control
AAV8-CRISPR
CT-2A
*
***
***
0.001
CT-2A WT
B.
CT-2A-Fluc -mCherry + AAV8
IVIS Bioluminescence measurement
Sack 3 mice per group
Day 0
Intracranial injection tumor and rAAV
Day 10
Humane end-point
C.
Tumor cells + AAV8 - Day 0
AAV8-control
AAV8-CRISPR
Bioluminescence
Sack 3 mice per group
Days (post-tumor implantation)
D.
SaCas9-KKH:
SaCas9-KKH
Fw
Rv
200bp
gRNA:
U6 promoter
sgRNA3
scaffold
78bp
E.
Tumor cells + AAV8 - Day 0
mCherry POS
ns
ns
CT-2A WT
AAV8-control
AAV8-CRISPR
Fold change (normalized to β-Actin)
SaCas9-KKH gRNA
F.
Tumor cells + AAV8 - Day 0
mCherry POS
*
mmu-miR-21a-5p fold change (normalized to U6)
G.
Day 3 post-tumor i.c. injection
CT-2A-mCherry
DAPI
H.
CT-2A 50,000
CT-2A 25,000
no tumor
Background
Bioluminescence
Day 3
I.
AAV8 - Day 3 and Day 6
ns
AAV8-control
AAV8-CRISPR
mmu-miR-21a-5p fold change (normalized to U6)
Liver

Figure S6. Testing of AAV8-CRISPR *in vitro* and in tumor-bearing mice. (A) *In vitro* transfection with the AAV8-CRISPR and AAV8-control vectors pre-packaging in CT-2A (left) and GL261 (right) glioma cells. mmu-miR-21a-5p expression was significantly decreased in CT-2A cells transfected with 400ng pre-packaged AAV8-CRISPR plasmid compared to CT-2A WT, SaCas9-KKH, and AAV8-control. Mmu-miR-21a-5p expression was reduced in GL261 cells transfected with pre-packaged AAV8-CRISPR compared to GL261 WT, SaCas9-KKH, and AAV8-Control, observed differences were not significant. Multi-comparison (Tukey) one-way ANOVA, $*p < 0.05$. (B) Schematic illustration of the experimental setup to deliver AAV8-CRISPR i.c. simultaneously with the tumor implantation. At day 0, mice were i.c. injected with 5×10^4 CT-2A cells resuspended in AAV8-CRISPR or AAV8-control (2×10^{10} gc/μL). Three mice per group were sacrificed for pathology purposes at day 10 post i.c. injections. (C) Bioluminescence signal was measured by IVIS, comparing mice i.c. injected with AAV8-CRISPR (n=8) or AAV8-control (n=8). Data are presented as the mean with SEM (error bars). Šídák's multiple comparisons two-way ANOVA showed no significant differences between groups. (D) Schematic display of a primer set flanking the SaCas9-KKH with an amplicon of 200bp and a primer set to amplify the gRNA with an amplicon of 78bp. (E) Mice implanted with CT-2A-FLuc-mCherry tumor cells and AAV (AAV8-CRISPR and AAV8-control) simultaneously. Brains were FACS-sorted for mCherry^{POS} cells 10 days post-i.c. injection. Fold expression of SaCas9-KKH and guide (sgRNA3) were measured by qRT-PCR. No significant differences were observed for SaCas9-KKH and gRNA expression levels in mCherry^{POS} tumor cells transduced by AAV8-CRISPR and AAV8-SaCas9-Control compared to CT-2A WT. Data represent triplicates (n=3 mice) and are presented as the mean with SEM (error bars). Multi-comparison (Tukey) one-way ANOVA, (ns = not significant). (F) Mmu-miR-21a-5p expression analysis of FACS sorted

mCherry^{POS} tumor cells showed a 180-fold reduction in tumor-bearing mice injected with AAV8-CRISPR compared to AAV8-control and to CT-2A WT cells. Data represents triplicates (n=3 mice) and are presented as the mean with SEM (error bars). Multiple-comparison (Turkey) one-way ANOVA, *$p<0.05$ (G) Tumor engraftment was validated by mCherry fluorescent cells visualized in the left striatum 3 days post-implantation of CT-2A-FLuc-mCherry cells. (Magnification 4x; scale bar 100 µm). (H) Mice that got injected with 25,000 (n=12) or 50,000 (n=12) CT-2A-FLuc-mCherry cells showed positive bioluminescence signal at day 3 post-tumor implantation prior to AAV8 administration compared to mice without tumor cells implanted and to the IVIS background signal. Data are presented as the mean with SEM (error bars), Multi-comparison (Tukey) one-way ANOVA, (ns = not significant). (I) Fold expression of mmu-miR-21a-5p was measured after 16 days in the livers of mice that got treated with AAV8 at day 3 and day 6 post-tumor implantation and showed no significant difference between AAV8-control (n=5) and AAV8-CRISPR treated mice (n=5). Data represent five replicates and are presented as the mean with SEM (error bars). Unpaired t test, (ns = not significant).

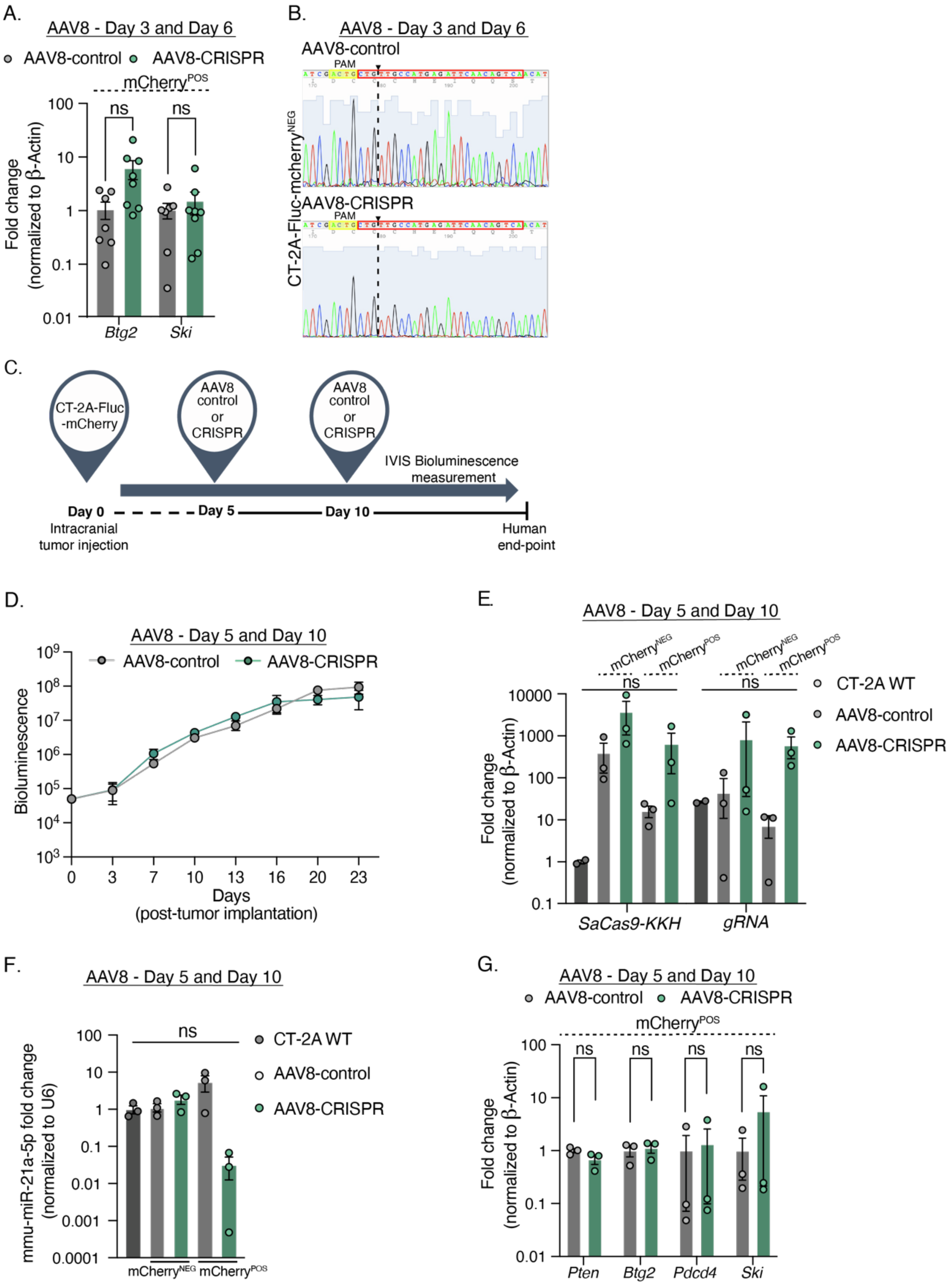
A.
AAV8 - Day 3 and Day 6
AAV8-control AAV8-CRISPR
mCherryPOS
ns
ns
Fold change (normalized to β-Actin)
Btg2
Ski
B.
AAV8 - Day 3 and Day 6
AAV8-control
PAM
AAV8-CRISPR
CT-2A-Fluc-mcherryNEG
C.
CT-2A-Fluc -mCherry
AAV8 control or CRISPR
AAV8 control or CRISPR
IVIS Bioluminescence measurement
Day 0
Intracranial tumor injection
Day 5
Day 10
Human end-point
D.
AAV8 - Day 5 and Day 10
AAV8-control
AAV8-CRISPR
Bioluminescence
Days (post-tumor implantation)
E.
AAV8 - Day 5 and Day 10
mCherryNEG
mCherryPOS
ns
CT-2A WT
AAV8-control
AAV8-CRISPR
Fold change (normalized to β-Actin)
SaCas9-KKH
gRNA
F.
AAV8 - Day 5 and Day 10
ns
CT-2A WT
AAV8-control
AAV8-CRISPR
mmu-miR-21a-5p fold change (normalized to U6)
mCherryNEG
mCherryPOS
G.
AAV8 - Day 5 and Day 10
AAV8-control
AAV8-CRISPR
mCherryPOS
ns
Fold change (normalized to β-Actin)
Pten
Btg2
Pdcd4
Ski

Figure S7. Timing of AAV treatment is important in tumor reduction. (A) Fold expression of mmu-miR-21a-5p downstream target genes (*Btg2*, *Ski*) were analyzed at day 16 post-tumor implantation, and no significant differences were observed in cells of mice treated with AAV8-CRISPR (n=8) or AAV8-control (n=8) at day 3 and day 6 post-tumor implantation for $mCherry^{POS}$ (tumor) cells. Data are presented as the mean with SEM (error bars). Unpaired t-test, (ns = not significant). (B) Representative panels of sanger sequencing of PCR products spanning mmu-miR-21a using 4Peaks software shows position of the sgRNA3 (red) and the nicking site (black dashed line) in the mmu-miR-21a reverse sequence traces in $mCherry^{NEG}$ cells of tumor-bearing mice i.c. injected with AAV8-CRISPR compared to the AAV8-control at day 3 and day 6 post-tumor implantation. Blue shading behind the peaks represents base quality. No indels were detected in the mmu-miR-21a sequence of both AAV-CRISPR and AAV8-control. (C) Schematic layout of the experimental design treating mice at day 5 and day 10 with AAV8-CRISPR or AAV8-control post-tumor implantation. Mice were sacrificed for pathology purposes at day 15 post i.c. injections. (D) Across all time points, bioluminescence signal (as measured by IVIS) did not significantly differ between mice injected with AAV8-CRISPR (n=11) or AAV8-control (n=11) at day 5 and day 10 post-tumor implantation. Data are presented as the mean with SEM (error bars). Šídák's multiple comparisons two-way ANOVA showed no significant differences between groups. (E) Fold expression of SaCas9-KKH and gRNA of mice treated at day 5 and day 10 with AAV8-CRISPR or AAV8-control post-tumor injection and FACS-sorted for $mCherry^{POS}$ tumor cells and $mCherry^{NEG}$ non-tumor cells at day 15 post-tumor implantation were measured by qRT-PCR and showed no significant differences between groups. Fold expression of gRNA levels were not significantly different between groups. Data represent triplicates (n=3 mice) and are presented as the mean with SEM (error bars). Multi-comparison (Tukey) one-way ANOVA. (F) Mmu-miR-

21a-5p expression analysis of FACS sorted mCherry^{POS} (tumor) cells and mCherry^{NEG} (non-tumor) cells in tumor-bearing mice administered with AAV8-CRISPR or AAV8-control showed no significant differences. Data represent triplicates and are presented as the mean with SEM (error bars). Multi-comparison one-way ANOVA, (ns = not significant). (G) Fold expression analysis of downstream mmu-miR-21a-5p mRNA targets (*Pten, Btg2, Pdcd4* and *Ski*) were analyzed at day 15 post-tumor implantation of mice administered with AAV8-CRISPR (n=3) or AAV8-control (n=3) at day 5 and day 10 post-tumor implantation for mCherry^{POS} (tumor) cells. Data and are presented as the mean with SEM (error bars). Unpaired t-test. (ns = not significant).

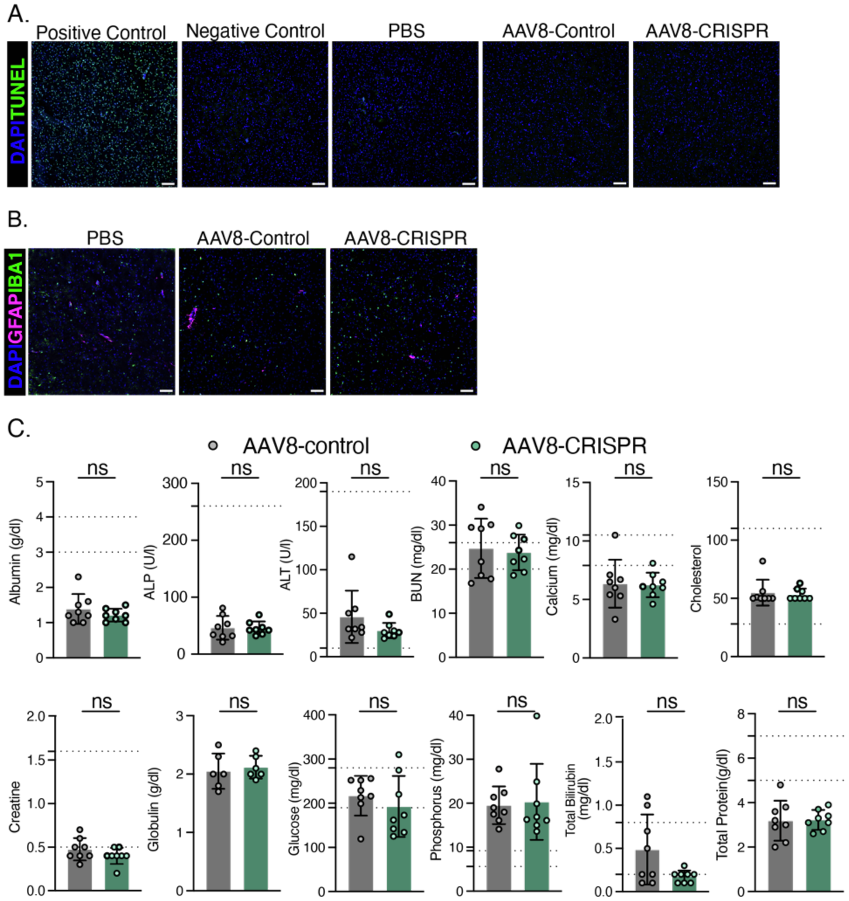

Figure S8. Local toxicity and blood toxicology following AAV injection. (A) AAV8-induced local toxicity was determined by terminal deoxynucleotidyl transferase dUTP nick end labeling (TUNEL) staining of brains from mice injected at day 7 with PBS, AAV8-control or AA8-CRISPR and sacrificed at day 14. The positive control, tissue incubated with DNAse I, showed DNA breaks in green in each cell stained for DAPI, no DNA breaks were observed in the negative control and PBS conditions, or brain sections of mice treated with AAV8-control or AA8-CRISPR. (B) Local toxicity post-AAV8-control or -AAV8-CRISPR injection compared to PBS was analyzed in brains stained for GFAP and IBA1. No increase in gliosis or microglia activation was observed. (C) Blood pathology was analyzed to determine AAV-induced toxicology after mice were treated with AAV-CRISPR or AAV8-control, no significant elevated levels of multiple blood

markers (Albumin; ALP; ALT; BUN; Calcium; Cholesterol; Creatine; Globulin; Glucose; Phosphorus; Total Bilirubin; Total Protein) were detected for both groups. Data represent two independent experiments (n=8 mice) and are presented as the mean with SEM (error bars). Multi-comparison one-way ANOVA, (ns = not significant).

SUPPLEMENTAL TABLES

Table S1. Overview of the top 5 predicted off-targets as determined by Cas-OFFinder.

Off-target	DNA	Location	Position	Direction	Mismatches
Off-target 1	ACTGTgGcATCTCATGaCAACAGAAAT	Chr4	153280201	+	3
Off-target 2	ACTGTTaAAcCTCATGGCAAtTATAGT	Chr11	112908309	-	3
Off-target 3	ACTGTTGAcTCTCcTGGCAAtCACGGT	Chr11	119227215	-	3
Off-target 4	tCTtTTGAAgCTCATGGCAACTTAAAT	Chr10	36314200	-	3
Off-target 5	AaTGTTGAtgaTCATGGCAACGTAAAT	Chr15	45517500	-	4

Table S2. Primers sets amplifying the off-targets.

Off-targets		Primer sequence (5'→3')	Amplicon size (bp)	Annealing temperature
Off-target 1	FW	CACTGCCCCAGCTGTAAGAT	248	60C
	RV	CCCTTCATCCACTTTGGCTT		
Off-target 2	FW	TAGAAAAGGTCGGCGCTGG	243	60C
	RV	CTCTTGAAGTATGGCCGTGGT		
Off-target 3	FW	CCCACACATGGTGATCCGA	247	60C
	RV	GGCCGGCTAAGATTCACCC		
Off-target 4	FW	ACAGCCAGGAAATATAGGATCATTC	236	60C
	RV	TTTTGGTGCAGCAGTTGGGT		
Off-target 5	FW	GTGGGGCTCAGATGCAGTAT	227	60C
	RV	TGACTAGGACACACAGCAAATGT		

Table S3. sgRNA sequences.

Name	Sequence	PAM
sgRNA1	GCCTTGTCGGATAGCTTATCAG	NNTGAT
sgRNA2	GTTATCAGACTGATGTTGACTG	NNGAAT
sgRNA3	GACTGTTGAATCTCATGGCAAC	NNCAGT
sgRNA4	GTTGAATCTCATGGCAACAGCA	NNCGAT
sgRNA5	GAGTCGATGGGCTGTCTGACAT	NNTGGT
sgRNA6	GGTCTGATAAGCTATCCGACAA	NNTGGT
sgRNA7	GTCAGTCTGATAAGCTATCCGA	NNAGGT
sgRNA8	GAGATTCAACAGTCAACATCAG	NNTGAT
sgRNA9	GCCATGAGATTCAACAGTCAAC	NNCAGT
sgRNA10	GACTGCTGTTGCCATGAGATTC	NNCAGT
sgRNA11	GGCCCATCGACTGCTGTTGCCA	NNAGAT

Table S4. Overview of the primer sets used to amplify mmu-miR-21a sequence to analyze for Sanger sequencing and NGS.

**Primer ID**		**Primer sequence (5'→3')**	**Amplicon size (bp)**	**Annealing temperature**
**Sanger sequencing**				
mmu-miR-21a	FW	GGTTCACCTAGAGTGGGAATCT	625	60C
mmu-miR-21a	RV	ATTGGGGTAGTCGTCACAGTC		
**NGS sequencing**				
mmu-miR-21a	FW	TTGACTGCAAACCATGATGCT	222	67C
mmu-miR-21a	RV	TGCTTTAAACCCTGCCTGAGC		
oS280 pUC19		CAGGGTTATTGTCTCATGAGCGG		

Table S5. TaqMan primers

**Primers**	**Assay ID**	**Manufacturer**
U6	001973	Thermo Fisher
Mmu-miR-21a-5p	000397	Thermo Fisher
Mmu-miR-21a-3p	002493	Thermo Fisher
pri-mmu-miR-21a	Mm03306822_pri	Thermo Fisher

Table S6. qRT-PCR Primer sequences

Primer ID	Primer sequence (5'→3')
SaCas9-KKH forward primer	AGAAGATGATTAACGAAATGCAGAAGCG
SaCas9-KKH reverse primer	TCGTAATTGAATGGATTATTCAGCAGATCTTC
gRNA forward primer	CTGTTGAATCTCATGGCAACGTTTTAGTAC
gRNA reverse primer	GAGATAAACACGGCATTTTGCCTT
GFP forward primer	CAAGGGCGAGGAGCTGTT
GFP reverse primer	TCGTCCATGCCGAGAGTGAT
Pten forward primer	TGGATTCGACTTAGACTTGACCT
Pten reverse primer	GCGGTGTCATAATGTCTCTCAG
Btg2 forward primer	TGAGCCACGGGAAGAGAAC
Btg2 reverse primer	GCCCTACTGAAAACCTTGAGTC
Pdcd4 forward primer	AAGACGACTGCGGAAAAATTCA
Pdcd4 reverse primer	CTTCTAACCGCTTCACTTCCATT
Ski forward primer	CAAAACAGACGACACTTCCTCA
Ski reverse primer	CAGCCGAGGCTCTTATTGGAG
Ucp1 forward primer	GCTTTGCCTCACTCAGGATTGG
Ucp1 reverse primer	CCAATGAACACTGCCACACCTC
Thap12 forward primer	TGAACAACCCGCATAGCAGACAC
Thap12 reverse primer	CCTGCTCTTGTTCAGAAGTCTCC
