# Supplementary material for: CRISPR targeting of mmu-miR-21a through a single adeno-associated virus vector prolongs survival of glioblastoma-bearing mice
Source: Mol Ther. 2024 Nov 19;33(1):133–51. doi: 10.1016/j.ymthe.2024.11.023 (PMC11764731; doi:10.1016/j.ymthe.2024.11.023)
Supplement: Document S2. Article plus supplemental information [file mmc2.pdf]

Molecular Therapy
Original Article

CRISPR targeting of mmu-miR-21a through a single adeno-associated virus vector prolongs survival of glioblastoma-bearing mice

Lisa Nieland,[1,2] Anne B. Vrijmoet,[1,10] Isabelle W. Jetten,[1,10] David Rufino-Ramos,[3,4,5] Alexandra J.E.M. de Reus,[1] Koen Breyne,[1] Benjamin P. Kleinstiver,[3,4,5] Casey A. Maguire,[1,6,7] Marike L.D. Broekman,[1,2,8,9] Xandra O. Breakefield,[1] and Erik R. Abels[2]

[1]Department of Neurology, Massachusetts General Hospital, Harvard Medical School, Boston, MA 02129, USA; [2]Department of Cell and Chemical Biology, Leiden University Medical Center, 2300 RC Leiden, the Netherlands; [3]Center for Genomic Medicine, Massachusetts General Hospital, Boston, MA 02114, USA; [4]Department of Pathology, Massachusetts General Hospital, Boston, MA 02114, USA; [5]Department of Pathology, Harvard Medical School, Boston, MA 02115, USA; [6]Department of Neurology, Massachusetts General Hospital, Boston, MA 02115, USA; [7]Harvard Medical School, Boston, MA 02116, USA; [8]Department of Neurosurgery, Haaglanden Medical Center, 2512 VA The Hague, the Netherlands; [9]Department of Neurosurgery, Leiden University Medical Center, 2300 RC Leiden, the Netherlands

Glioblastoma (GB), the most aggressive tumor of the central nervous system (CNS), has poor patient outcomes with limited effective treatments available. MicroRNA-21 (miR-21(a)) is a known oncogene, abundantly expressed in many cancer types. miR-21(a) promotes GB progression, and lack of miR-21(a) reduces the tumorigenic potential. Here, we propose a single adeno-associated virus (AAV) vector strategy targeting mmu-miR-21a using the *Staphylococcus aureus* Cas9 ortholog (SaCas9) guided by a single-guide RNA (sgRNA). Our results demonstrate that AAV8 is a well-suited AAV serotype to express SaCas9-KKH/sgRNA at the tumor site in an orthotopic GB model. The SaCas9-KKH induced a genomic deletion, resulting in lowered mmu-miR-21a levels in the brain, leading to reduced tumor growth and improved overall survival. In this study, we demonstrated that disruption of genomic mmu-miR-21a with a single AAV vector influenced glioma development, resulting in beneficial anti-tumor outcomes in GB-bearing mice.

INTRODUCTION

Glioblastoma (GB) is the most common and aggressive form of malignant primary brain tumor of the central nervous system (CNS).[1] Despite advances in local therapy,[2] the complex heterogeneity results in therapeutic resistance, high recurrence rates, and poor median survival outcomes of ∼15 months.[2,3] The 5-year overall survival rate is less than 7% with an estimated annual mortality of over 10,000 individuals in the United States.[4] The current standard of care involves surgical resection followed by radiation therapy and temozolomide; this treatment standard has not changed over the past two decades.[3] Therefore, the development of improved treatment strategies is urgently needed.

The expression of microRNA-21 (miR-21) (human hsa-miR-21 or murine mmu-miR-21a) is increased in nearly all cancers, including GB.[5,6] miR-21 was first discovered to be increased in human GB by Chan et al.,[7] and since then many other studies have reported upregulated miR-21 levels in patient-derived glioma tumor samples and cerebrospinal fluid from patients.[7–9] Elevated miR-21 levels have been negatively correlated with malignancy and patients with increased miR-21 levels have a worse prognosis.[10] Over the past years, miR-21(a) has therefore gained interest as a potential therapeutic target in many cancers, including GB.[11]

Clustered regularly interspaced short palindromic repeats (CRISPR) and CRISPR-associated protein (Cas) have had a major impact in the field of gene therapy in the last decade.[12] Cas9 nuclease guided by a single-guide RNA (sgRNA) has been extensively used for genome editing.[13] Various delivery methods for Cas nucleases have been developed for cultured cells, including plasmid and RNA transfections. Additionally, viral-based delivery methods, including lentivirus, adenovirus, and adeno-associated virus (AAV) vectors, as well as ribonucleoprotein (RNP) electroporation,[14] have also been explored. Since AAV vectors are severely constrained by their cargo limits (∼4.7 kb), precise and efficient *in vivo* gene editing is challenging to achieve. To utilize AAV vectors for precise and efficient *in vivo* gene editing, we therefore used hypercompact CRISPR-Cas nucleases that were developed to be compatible with a single AAV-mediated gene-editing therapy, such as *Staphylococcus aureus* Cas9 (SaCas9). SaCas9 is one of the smaller Cas9 orthologs, consisting of 1,368 amino acids.[15,16] SaCas9 variants, such as SaCas9-KKH, have been reported to have improved genome-wide editing accuracy and

Received 23 May 2024; accepted 15 November 2024;
https://doi.org/10.1016/j.ymthe.2024.11.023.

[10]These authors contributed equally

Correspondence: Erik R. Abels, Ph.D., Department of Cell and Chemical Biology, Leiden University Medical Center, 2300 RC Leiden, the Netherlands
E-mail: e.r.abels@lumc.nl

Published by Elsevier Inc. on behalf of The American Society of Gene and Cell Therapy.
This is an open access article under the CC BY license (http://creativecommons.org/licenses/by/4.0/).

single-base mismatch discrimination due to an expanded protospacer adjacent motif (PAM) requirement.[17]

The advantages of AAV-based gene therapy delivery are reflected in the number of clinical trials utilizing AAV vectors worldwide.[18] AAV-based gene therapy has attracted considerable interest in therapy development for GB patients.[19,20] In the past decade, scientists have tried to optimize AAV capsids to efficiently transduce GB cells.[21–23] For example, an AAV9 vector expressing the secreted anti-cancer agent soluble tumor necrosis factor-related apoptosis-inducing ligand (sTRAIL) showed potential for treating GB in a patient-derived orthotopic xenograft model.[24] AAV vectors are capable of transducing both dividing and non-dividing cells,[25,26] and their small size allows them to better penetrate solid tumors, such as GBs.[27] Recently, the success of all-in-one AAV vectors for precision Cas9 genome editing via homology-directed repair *in vivo* has been reported,[28–30] and multiple groups have used smaller Cas9 orthologs to develop single-AAV vector approaches.[31–33]

Here, we describe an all-in-one AAV vector with a cargo of ~4.5 kb, co-expressing SaCas9-KKH[34] guided by a single sgRNA to target the mmu-mir-21a locus. We provide evidence that successful mmu-miR-21a gene disruption through administration of this AAV vector hampers tumor progression, resulting in improved overall survival in a GB mouse model.

RESULTS

Efficient mmu-miR-21a editing of mouse GB *in vitro*

Previously, we have shown that the CRISPR-Cas12a genome-editing approach can efficiently knock out mmu-miR-21a, thereby reducing tumor growth in multiple mouse and human GB models.[35] The mmu-miR-21a hairpin, encoded in the precursor mmu-miR-21a (pre-mmu-miR-21a) sequence driven by the miR-21a promotor, miPPR-21,[36] is found within the 3′ untranslated region (UTR) of the vacuole membrane protein 1 (*Vmp1*) (known as *Tmem49*) gene located on chromosome 11 in mice and chromosome 17 in humans.[37] We schematically illustrated the location of sgRNA3, the PAM sequence, and the nicking site within the mmu-mir-21a locus, highlighting the most frequent indels relative to the hairpin structure and proposed consequence of the indels on the folding structure and subsequent Dicer processing of the pre-mmu-miR-21a (Figure 1A). To target mmu-miR-21a, we used the AAV-compatible SaCas9-KKH.[17] We tested the SaCas9-KKH efficiency with 11 different sgRNAs, specifically designed to target the mmu-mir-21a locus (Figure S1A), which were each cloned into an individual entry vector pUC19 and co-transfected with the SaCas9-KKH-expressing plasmid driven by a cytomegalovirus (CMV) enhancer fused to the chicken beta-maa (CAG) promoter[17] (Figure S1B). After measuring mmu-miR-21a-5p expression in mouse GB cell lines to screen the target efficiency of 11 different pUC19 plasmids, each expressing a single sgRNA driven by U6 promoter (1–11) (Figures S1C and S1D), we selected guides sgRNA3, sgRNA9, and sgRNA10 based on mmu-miR-21a-5p reduction in both GL261 and CT-2A murine tumor cells (Figures 1B and S1E). This mmu-miR-21a-5p arm was 516- and 520-fold upregulated compared to the mmu-miR-21a-3p arm in both GL261 and CT-2A wild-type (WT) cells, respectively (Figure S2A), indicating that the mmu-miR-21a-3p arm is expressed at low levels in mouse GB cells (Figure S2B). No significant difference in mmu-miR-21a-3p expression in GL261 and CT-2A cells was observed upon gene editing by co-transfection of the SaCas9-KKH plasmid and the sgRNA3-expressing plasmid or after transfection with a single plasmid co-expressing SaCas9-KKH and sgRNA3 (referred to as the "all-in-one" plasmid) (Figure S2C). We then checked the fold expression of primary-(pri)-mmu-miR-21a and showed reduced expression levels upon gene editing; however, no significant differences were found (Figure S2D). Next, phosphatase and tensin homolog (*Pten*), a downstream mRNA target of the mmu-miR-21a-5p arm, showed 10-fold and ~5-fold upregulation in all-in-one CRISPR-edited GL261 cells and CT-2A cells, respectively. B cell translocation gene 2 (*Btg2*) was not significantly upregulated upon gene editing (Figure S2E). In contrast, downstream mRNA targets of the mmu-miR-21a-3p arm, including uncoupling protein 1 (*Ucp1*) and Thanatos Associated Protein (THAP) domain containing 12 (*Thap12*), showed no differences between unedited WT cells and genome-edited cells (Figure S2F). Cells co-transfected with the SaCas9-KKH plasmid and the sgRNA3 plasmid showed significantly reduced mmu-miR-21a-5p expression levels in both GB cell lines compared to WT cells and cells that were transfected with only the SaCas9-KKH-expressing plasmid (Figure 1B). Therefore, sgRNA3 was used for further experiments throughout this study. Subsequently, GB cells co-transfected with both SaCas9-KKH and sgRNA3 expressing plasmids resulted in an insertion or deletion (indel) in CRISPR-edited cells compared to CT-2A WT or cells transfected with only the SaCas9-KKH plasmid, as determined by Sanger sequencing (Figure 1C). Next-generation sequencing (NGS) showed 59.78% modified reads after gene editing with SaCas9-KKH and sgRNA3 compared to CT-2A WT, which had 0.49% modified reads. Transfection with the SaCas9-KKH plasmid alone resulted in 1.62% modified reads (Figure 1D). CRISPR-edited cells showed a deletion around the nicking site compared to CT-2A WT and to cells transfected with the SaCas9-KKH plasmid non-edited reference sequence (Figure 1E). Unedited pre-mmu-miR-21a forms a hairpin loop structure as schematically illustrated (Figure S3A). We used RNAfold (http://rna.tbi.univie.ac.at/cgi-bin/RNAWebSuite/RNAfold.cgi) to predict the RNA folding and the hairpin loop structures with minimal free energy (MFE) *in silico*. We analyzed the RNA folding after deleting one of the three most frequent indels observed: (1) indel "C"; (2) indel "G"; (3) indel "T"; or combinations 1-2 indel "GC," 1-3 indel CA, or 1-2-3 indel "GCA," which showed different folding structures important for Dicer processing of the pre-mmu-miR-21a(Figures 1E [shown in reverse complement] and S3B). The indel distribution in the sgRNA3 CRISPR-edited cells showed the most frequent indels of 1 bp (17.6%) and 2 bp (18.3%) in size (Figure 1F). Taken together, we showed that sgRNA3 co-transfected with the SaCas9-KKH plasmid was most effective in targeting mmu-miR-21a in murine GB cells, resulting in reduced mmu-miR-21a-5p expression levels due to indels resulting from genome editing.

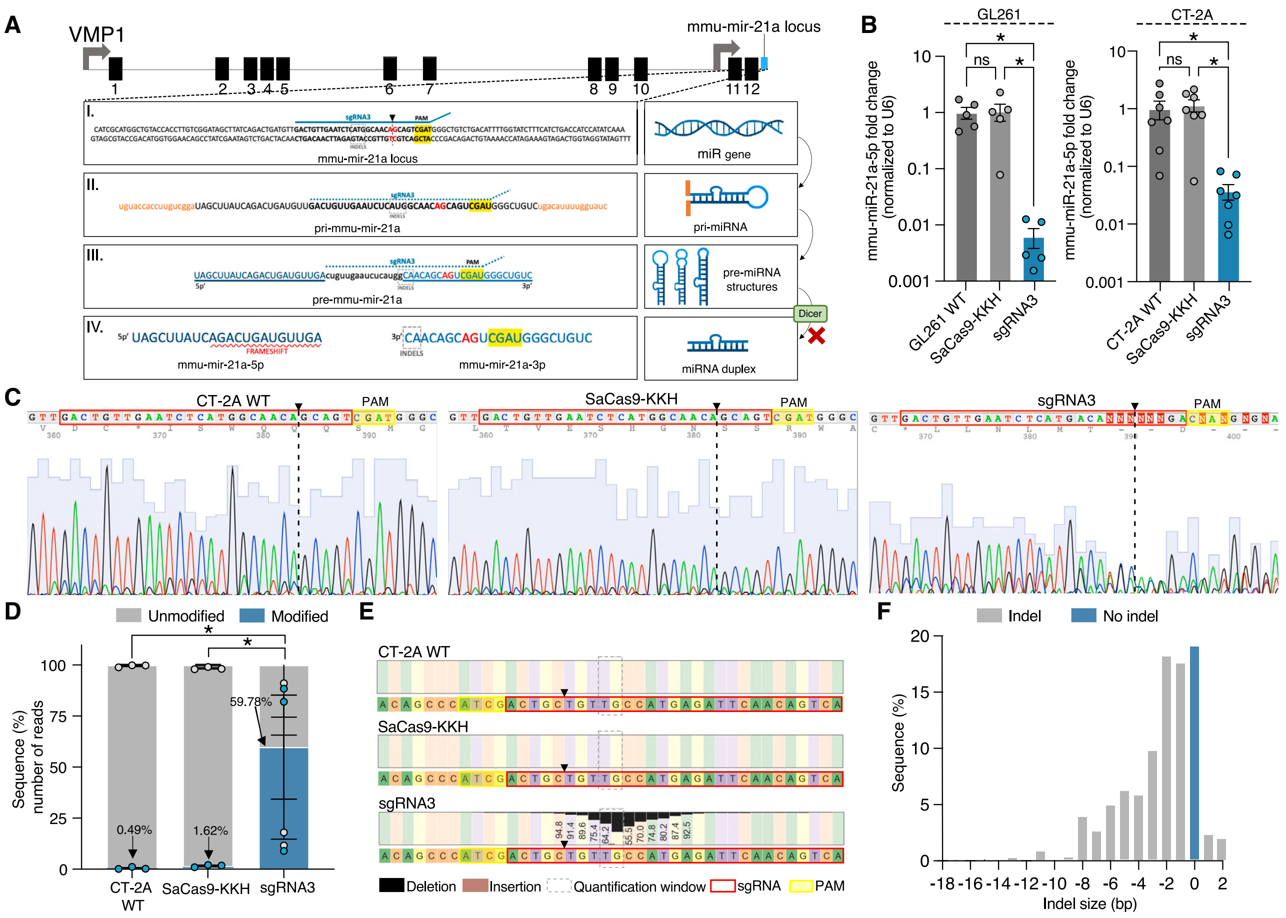

Figure 1. Efficient mmu-miR-21a editing of mouse GB *in vitro*

(A) Structure of the *Vmp1* gene with the mmu-miR-21a located at chromosome 11. Exon numbers are noted. The gray arrow shows the miR-21a promoter, miPPR-21. The light blue box indicates the location of the mature pre-mmu-miR-21a sequence. (I) Double-stranded DNA of the mmu-miR-21a gene is displayed in black. The position of sgRNA3 (TCAGACTGATGTTGACTGTTGAATCTCATGGCAAC) is annotated with a blue line and the PAM sequence is highlighted in yellow. A dotted vertical line shows the nicking site located between the "A" and "G" highlighted in red. (II) The pri-mmu-mir-21a single-stranded RNA sequence is shown with a dotted green line indicating the location of guide sgRNA3 and the PAM sequence highlighted in yellow. (III) The pre-mmu-mir-21a hairpin loop is illustrated with the -5p arm in dark blue and the -3p arm in light blue. Proposed mechanism showing different secondary folding structures upon gene editing and subsequent faulty Dicer processing of pre-mmu-miR21a. (IV) The miR-21 duplex is shown with the mature mmu-miR-21a-5p and the mmu-miR-21a-3p arm. (B) After co-transfection with the sgRNA3 (blue) and the SaCas9-KKH expressing plasmid for 72 h, mmu-miR-21a-5p-fold expression levels were measured by RT-qPCR normalized to the housekeeping gene U6. A 160-fold and 30-fold reduction of mmu-miR-21a-5p levels was found in the CRISPR-edited GB cell lines GL261 and CT-2A, respectively, compared to GL261 or CT-2A WT and single transfected cells with the SaCas9-KKH plasmid. Data represent 5–7 replicates and are presented as the mean with SEM (error bars). Multi-comparison one-way ANOVA, *$p < 0.05$. (C) Sanger sequencing of PCR products spanning mmu-miR-21a using 4Peaks software shows position of the sgRNA3 (red) and the PAM (yellow) sequence and the nicking site (black dashed line) in the mmu-miR-21a reverse-complement sequence traces of CT-2A WT compared to SaCas9-KKH and genetically edited CT-2A cells using sgRNA3. Blue shading behind the peaks represents base quality. WT and SaCas9-KKH show single-base peaks, while the sgRNA CRISPR-edited cells show double peaks and missing nucleotides (N) due to a disruption of the mmu-miR-21a sequence. (D) NGS was performed on a 200-bp PCR product spanning mmu-miR-21a; CRISPR analysis was conducted using CRISPResso2 to investigate the insertion or deletion (indel) formation within a specified quantification window. CT-2A WT (0.49%) and SaCas9-KKH (1.62%) were not modified (blue) compared to sgRNA3 CRISPR-edited cell (59.78%) indel modifications in the targeted mmu-miR-21a sequence. Data represent triplicates and are presented as the mean with SEM (error bars). Multiple comparisons two-way ANOVA, *$p < 0.05$. (E) Nucleotide percentage quantification was performed and showed deletions (black) in the region of interest in the sgRNA3 CRISPR-edited sequence compared to WT and SaCas9-KKH control. (F) The indel size (bp) was determined showing 1-bp deletion as the most commonly observed indel.

Downregulation of mmu-miR-21a expression levels using an all-in-one sgRNA3-SaCas9-KKH plasmid

To target mmu-miR-21a with a single plasmid, we generated a construct expressing SaCas9-KKH under the CMV enhancer fused to a CAG promoter, fused to a 3xFLAG-tag, a peptide 2A (p2A) sequence, a self-cleaving peptide allowing independent genes to be transcribed as a single mRNA, and a green fluorescent protein (GFP) together with the sgRNA3 driven by the U6 promoter (Figure 2A). Subsequently, we determined whether CRISPR editing mediated by the all-in-one plasmid could effectively target mmu-miR-21a

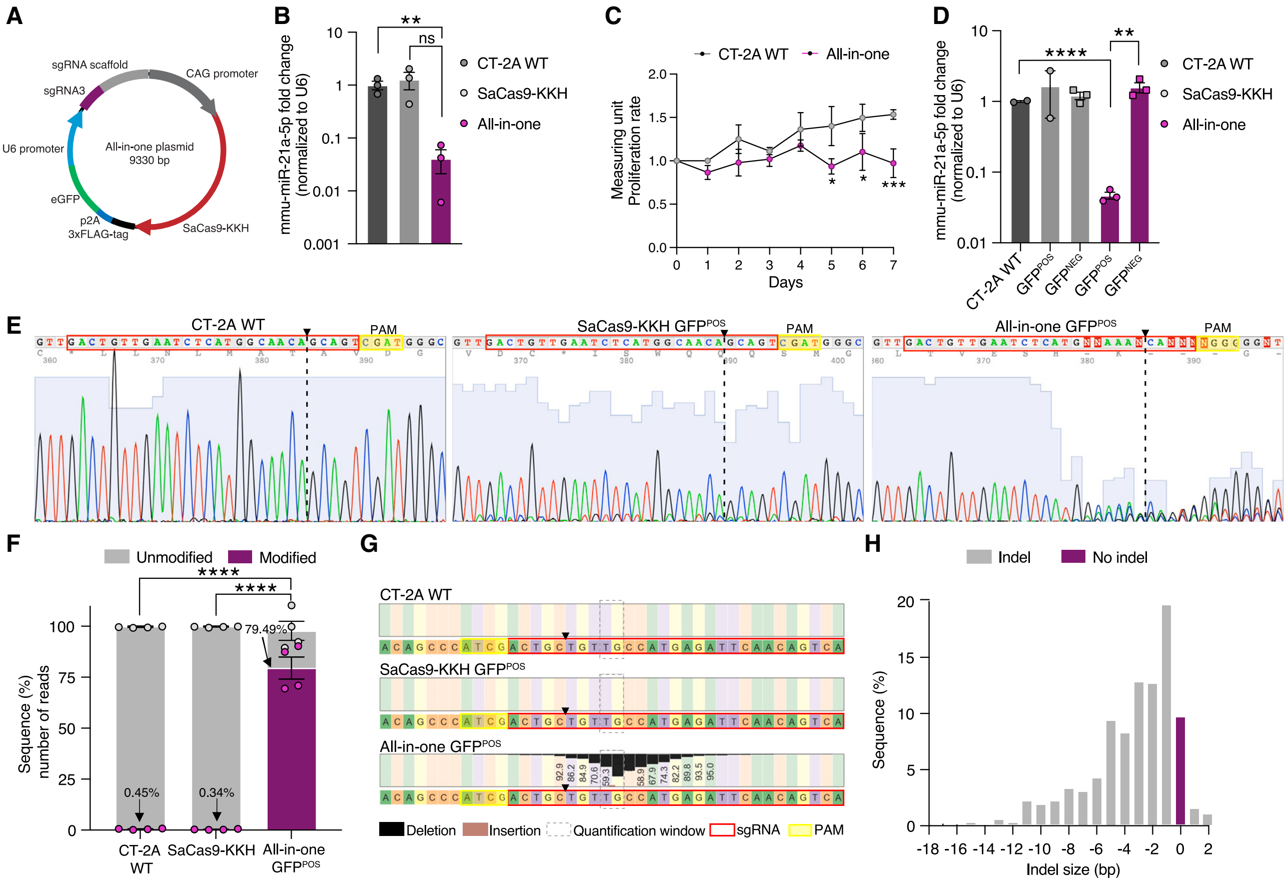

Figure 2. Downregulation of mmu-miR-21a expression levels using an all-in-one sgRNA3-SaCas9-KKH plasmid

(A) Schematic display of the all-in-one plasmid containing SaCas9-KKH under the CMV promoter tagged with both 3xFLAG-tag, a peptide 2A (p2A) sequence, and GFP together with the U6 promoter encoding the sgRNA3. (B) mmu-miR-21a-5p expression was measured by RT-qPCR and normalized to the U6 housekeeping gene. CRISPR-edited CT-2A cells after transfection with the all-in-one construct targeting mmu-miR-21a (purple) had 25-fold reduced mmu-miR-21a-5p levels compared to CT-2A WT. Data represent triplicates and are presented as the mean with SEM (error bars). Unpaired t test, $^{**}p < 0.01$, (ns, not significant). (C) Proliferation rates were measured every 24 h for 5 days with the WST reduction assay. CT-2A mmu-miR-21a-edited cells (all-in-one GFP^{POS}) were plated after cells were sorted for GFP^{POS} and proliferation was measured over time. All-in-one transfected cells proliferated at a lower rate as compared to CT-2A WT cells. Results display the ratio of mmu-miR-21a edited cells. Data are presented as the mean with SEM (error bars). Šídák's multiple comparisons two-way ANOVA, $^{*}p < 0.01$, $^{***}p < 0.001$. (D) RT-qPCR showed a 25-fold reduced mmu-miR-21a-5p expression of CT-2A cells single transfected with the all-in-one construct (purple) sorted for GFP^{POS} compared to GFP^{NEG}, non-transfected CT-2A WT cells, and single-transfected SaCas9-KKH (gray) GFP^{POS} and GFP^{NEG} control. Data represent triplicates and are presented as the mean with SEM (error bars). Unpaired t test, $^{**}p < 0.01$, $^{****}p < 0.0001$. (E) Sanger sequencing of PCR products spanning mmu-miR-21a using 4Peaks software shows the position of the sgRNA3 (red) and the PAM (yellow) sequence and the cleavage site (black dashed line) in the mmu-miR-21a reverse-sequence traces of CT-2A WT compared to SaCas9-KKH GFP^{POS} and genetically edited CT-2A cells using the all-in-one plasmid sorted for GFP^{POS}. Blue shading behind the peaks represents base quality. WT and SaCas9-KKH show single-base peaks, while the sgRNA CRISPR-edited cells show double peaks and missing nucleotides (N) due to a disruption of the mmu-miR-21a sequence. (F) NGS was performed on a specific PCR product spanning mmu-miR-21a, and CRISPR analysis was conducted using CRISPResso2 to investigate the indel formation within a specified quantification window. Indel modifications are shown in purple for CT-2A WT (0.45%), SaCas9-KKH only (0.34%), and the all-in-one CRISPR-edited cell (49.49%) indel modifications in the targeted mmu-miR-21a sequence. Data represent four replicates and are presented as the mean with SEM (error bars). Multiple comparisons two-way ANOVA, $^{****}p < 0.0001$. (G) Nucleotide percentage quantification was performed and showed deletions (black) in the region of interest in the sgRNA3 CRISPR-edited sequence compared to WT and SaCas9-KKH control. (H) The indel size (bp) was determined showing 1-bp deletion as the most commonly observed indel.

sequences. Mmu-miR-21a levels were significantly reduced compared to CT-2A WT and SaCas9-KKH control after *in vitro* transfection (Figure 2B). CT-2A cells transfected with the all-in-one plasmid resulted in downregulated of mmu-miR-21a levels and reduced proliferation rates compared to CT-2A WT cells (Figure 2C). A plasmid titration evaluation of the all-in-one construct showed increased transfection efficiency and reduced mmu-miR-21a-5p expression levels upon transfection with higher all-in-one dosages (400, 1,000, and 1,600 ng). However, the highest dose (1,600 ng) did not show more suppression of mmu-miR-21a-5p, probably due to the increased cell death observed (Figure S4A). The transfection efficiency was 60% based on the number of GFP-positive (GFP^{POS}) cells

determined by fluorescence microscopy in the 1,000-ng dose condition (Figure S4B). Immunofluorescent staining of the 3xFLAG-tag showed co-expression with GFP on representative images (Figure S4C). Next, we used fluorescence-activated cell sorting (FACS) to sort the all-in-one transfected cells for GFP^{POS} and GFP-negative (GFP^{NEG}) populations and we determined 3xFLAG-tag in GFP^{POS} sorted cells post transfection with the all-in-one plasmid, but not in CT-2A WT (Figure S4D). We determined mmu-miR-21a-5p expression levels showing that GFP^{POS} sorted cells, post all-in-one transfection, had significantly lower mmu-miR-21a-5p expression levels compared to the GFP^{NEG} population and to both CT-2A WT and SaCas9-KKH transfected CT-2A cells (Figure 2D). GFP^{POS} sorted cells were analyzed for the disrupted genomic sequence by Sanger sequencing and indels were observed in the all-in-one GFP^{POS} cells compared to cells transfected with the SaCas9-KKH plasmid and CT-2A WT cells (Figure 2E); the control conditions all-in-one GFP^{NEG} and SaCas9-KKH GFP^{NEG} did not show any changes in the mmu-miR-21a sequence. Moreover, an indel was observed in cells co-transfected with the SaCas9-KKH plasmid and the sgRNA3 expressing plasmid sorted for GFP^{POS}, but not in the GFP^{NEG} population of the co-transfected cells (Figure S4E). The cells co-transfected with the SaCas9-KKH plasmid and the sgRNA3-expressing plasmid sorted for GFP^{POS} showed a 1.5-fold reduced mmu-miR-21a-5p expression level compared to GFP^{NEG} (Figure S4F). NGS analysis showed 79.49% of the reads modified after gene editing with the all-in-one construct compared to CT-2A WT (0.45% modified reads) and cells transfected with the SaCas9-KKH plasmid (0.34% modified reads) (Figure 2F). The all-in-one CRISPR-edited cells showed a deletion around the cleavage site compared to CT-2A WT cells and cells transfected with SaCas9-KKH plasmid (Figure 2G). The indel distribution of the all-in-one mmu-miR-21a edited cells showed the most frequent indel of 1 bp (19.7%) (Figure 2H). Importantly, SaCas9-KKH editing showed minimal off-target effects in the mouse genome in the top five predicted off-target sites (Table S1) based on NGS analysis of amplified regions including the off-target sites (Table S2) in CRISPR-edited all-in-one GFP^{POS} sorted cells compared to non-edited WT CT-2A cells (Figure S4G). We conclude that the all-in-one gene-editing approach was successful in disrupting sequences encoding mmu-miR-21a in GB cells.

AAV8-GFP vector transduction profiles in murine GB cell lines

Multiple AAV capsids have been engineered to improve cancer cell-specific transduction.[38] Previously, it has been shown that the AAV8 serotype enhances therapeutic efficacy in the CNS[39] upon transduction of a murine GB model (GL261).[40] Therefore, we tested the AAV8 serotype encoding GFP under the control of a CAG promoter,[41] with the vector expression cassette flanked by inverted terminal repeats (ITRs) (Figure 3A). GB cell lines (CT-2A and GL261) were transduced *in vitro* and evaluated 7 days post transduction. Representative images revealed co-localization of GFP with Ki-67, showing the transduced proliferative GB cells after AAV8-GFP transduction (Figure S5A). The number of transduced cells (GL261 and CT-2A), as determined by GFP expression, was quantified (~55% for both cell lines). Co-localization of GFP and Ki-67 was quantified as a percentage of total cells (GL261 ~50% and CT-2A ~35%, respectively) (Figure S5B). To validate the transduction efficiency *in vivo*, mouse brains were implanted with GB cells (CT-2A-mCherry) co-expressing firefly luciferase (FLuc) (CT-2A-FLuc-mCherry) and tumor engraftment in the mouse striatum was monitored using an *in vivo* imaging system (IVIS) (Figure S5C). At day 7 post tumor implantation, mice were injected intracranially (i.c.) with a single dose of AAV8-GFP or PBS as a control and, on day 14 post tumor implantation, mice were sacrificed (Figure 3B). GFP gene expression levels were significantly higher in mice injected with AAV8-GFP compared to mice injected with PBS control in the tumor-bearing hemisphere, the left hemisphere (LH). No significant difference between groups was observed in the (non-tumor) right hemisphere (RH) (Figure 3C). Although previous studies reported limited to no expression of AAV8 in the contralateral side, positive GFP expression was observed in the RH, suggesting that the AAV8-GFP transferred to the non-injected hemisphere.[42–44] Moreover, brains were analyzed to determine transgene expression by immunofluorescence (IF) and AAV8-GFP showed the highest rate of transduction at the tumor border (Figure 3D). In cells transduced with AAV8-GFP, GFP did not colocalize with astrocytes expressing glial fibrillary acidic protein (GFAP) or microglia positive for ionized calcium-binding adaptor molecule 1 (IBA1) at the tumor border. However, the AAV8 serotype did not solely target mCherry-positive ($mCherry^{POS}$) tumor cells. We observed GFP in oligodendrocytes positive for oligodendrocyte transcription factor 2 (OLIG2) and neurons expressing NEUN, a mature neuronal marker at the tumor periphery (Figure 3E). Pre-clinical biodistribution studies have shown that AAV capsids are highly sequestered by the liver in a species-independent manner.[45] Liver uptake of AAV vectors causing AAV-mediated hepatotoxicity is commonly observed as an adverse clinical effect.[46,47] In our AAV8-GFP screening by RT-qPCR, GFP expression levels were low in the liver, lungs, and spleen (Figure S5D). Additionally, the flow cytometric analysis of the brain-derived dissociated tumor tissue showed $mCherry^{POS}$ tumor cells (13.4%), GFP^{POS} AAV8 transduced cells (3.6%), and $mCherry^{POS}/GFP^{POS}$ (3.4%) double-positive cells (Figure 3F). Since GB cells rapidly divide, AAV genomes can be diluted out, resulting in transient transduction,[48] so this is likely an underestimation of total tumor cell transduction. Taken together, the AAV8 serotype is capable of transducing CT-2A tumor cells in GB-bearing mice when injected locally at the tumor site.

In vivo genome editing of miR-21 by SaCas9-KKH delivered by AAV8 vector in GB-bearing mice

To test genome editing of mmu-miR-21a as a therapeutic intervention, we generated a smaller cassette (~4.5 kb) encoding both SaCas9-KKH and sgRNA3 between ITRs of the AAV vector construct. The all-in-one CMV-SaCas9-KKH-U6-sgRNA3 plasmid (AAV8-CRISPR) and the CMV-SaCas9-KKH-U6 plasmid lacking an sgRNA (AAV8-control) were generated for *in vivo* application (Figure 4A). Following *in vitro* transfection with the AAV8-CRISPR pre-packaged plasmid, mmu-miR-21a-5p expression levels were significantly reduced in CT-2A cells compared to AAV8-control. No downregulation and no significant differences between

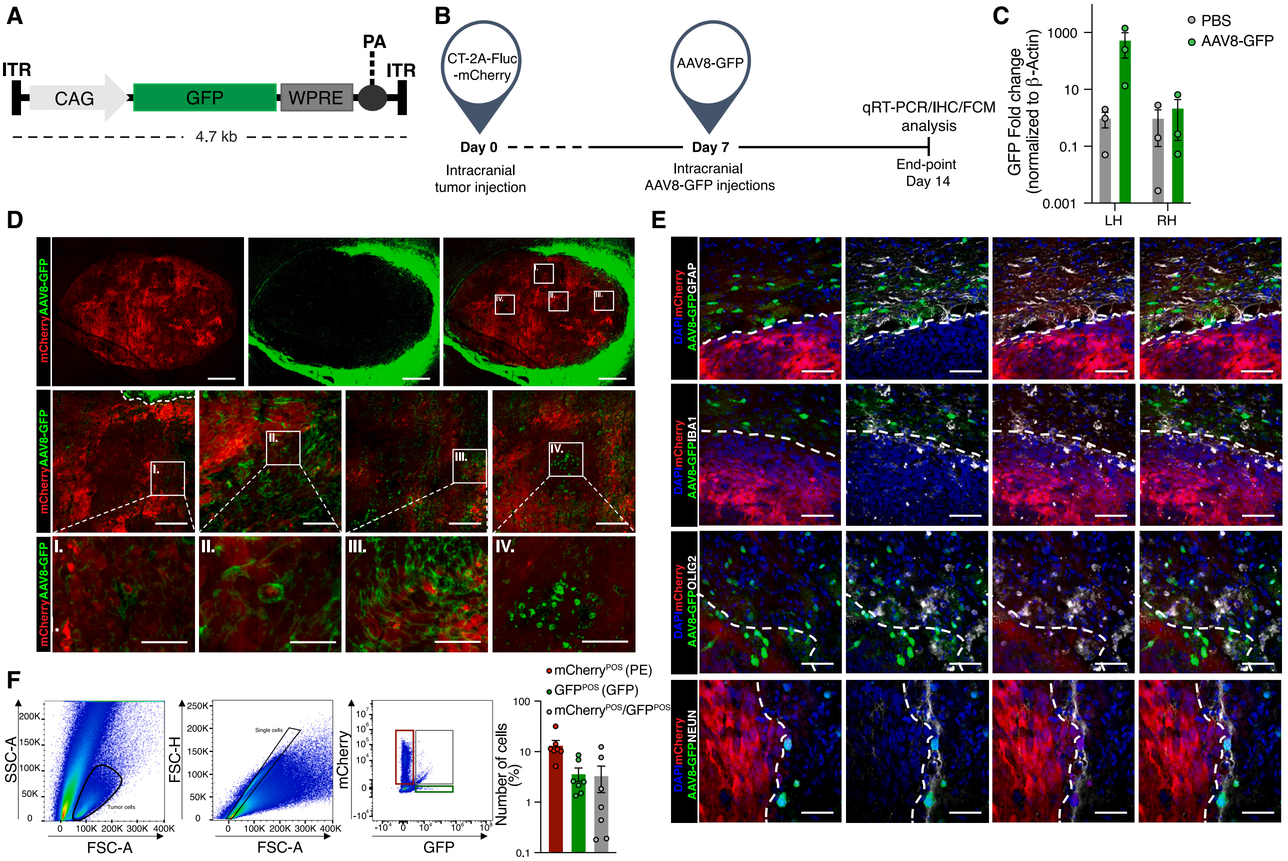

Figure 3. AAV8-GFP vector transduction profiles in murine GB cell lines

(A) Schematic representation of the AAV8 capsid encoding GFP under the CAG promoter flanked by ITRs. (B) Schematic illustration of *in vivo* experimental setup. At day 0, mice were intracranially injected in the left striatum with CT-2A-FLuc-mCherry tumor cells and measured for tumor growth by IVIS bioluminescence over time. At day 7 post tumor implantation, AAV8 or PBS was intratumorally injected at the same coordinates as used for tumor implantation and mice were sacrificed on day 14 for pathologic analyses (RT-qPCR, IHC, fluorescent microscopy). (C) GFP fold change was determined by RT-qPCR and showed 2,188-fold higher GFP levels (green) in the (tumor implanted) LH compared to PBS control (gray). GFP levels (green) were 3-fold higher compared to PBS in the (non-tumor) RH; no significant differences were observed. Data represent six replicates and are presented as the mean with SEM (error bars). Multiple comparisons two-way ANOVA. (D) Representative images of brain tissue of tumor (CT-2A-FLuc-mCherry)-bearing mice i.c. injected with AAV8-GFP on day 7. AAV8-GFP transduced tumor cells (green) were visible in the tumor vicinity and in the tumor mass. (E) Representative images of co-staining of AAV8-GFP transduced cells with GFAP, IBA1, OLIG2, or NEUN at the CT-2A-FLuc-mCherry tumor border (tumor border displayed as white dashed line). (F) Representative flow cytometry plots show the gating strategy for tumor cells, showing mCherry^{POS} and GFP^{POS} populations. mCherry^{POS} (13.4%), GFP^{POS} (3.6%), double-positive for mCherry^{POS} and GFP^{POS} (3.4%). Bar graphs represent quantification as a percentage of the number of cells. Each data point represents one mouse (n = 7) and is presented as the mean with SEM (error bars). Multi-comparison one-way ANOVA, $^{**}p < 0.001$.

groups were observed in GL261 cells (Figure S6A). To validate the mmu-miR-21a CRISPR-based editing approach in a GB mouse model, we delivered the AAV vectors simultaneously with the tumor cells (CT-2A-FLuc-mCherry) locally at the tumor site by i.c. injection into the left striatum, as schematically illustrated (Figure S6B). We monitored tumor growth over time using IVIS (Figure S6C). Two primer sets were designed to measure SaCas9-KKH and sgRNA RNA expression levels (Figure S6D). Mice that were simultaneously implanted with CT-2A-mCherry cells and AAV8 vectors on day 0 showed ~17-fold increased expression of SaCas9-KKH compared to non-treated CT-2A WT. gRNA expression was 7.0-fold higher in mice treated with AAV8-CRISPR compared non-treated CT-2A (Figure S6E). Three mice per group were sacrificed on day 10 post combined tumor and treatment injection and tumor cells in ipsilateral hemispheres were dissociated to analyze the therapeutic effect on gene expression. Expression levels of mmu-miR-21a-5p were 180-fold reduced in tumor-bearing brain treated with AAV8-CRISPR compared to AAV8-control and non-treated CT-2A tumor-bearing brain (Figure S6F). To evaluate a potential therapeutical effect, mice were treated with either AAV8-CRISPR or AAV8-control on day 3 and day 6 post tumor implantation, as schematically displayed (Figure 4B). Before AAV8 administration, we confirmed tumor engraftment on day 3 post tumor injection by immunofluorescence of mCherry^{POS} (Figure S6G) tumor cells and bioluminescence (Figure S6H). Mice treated on days 3 and 6 showed arrested tumor growth between day 13 and day 23 with significantly reduced tumor

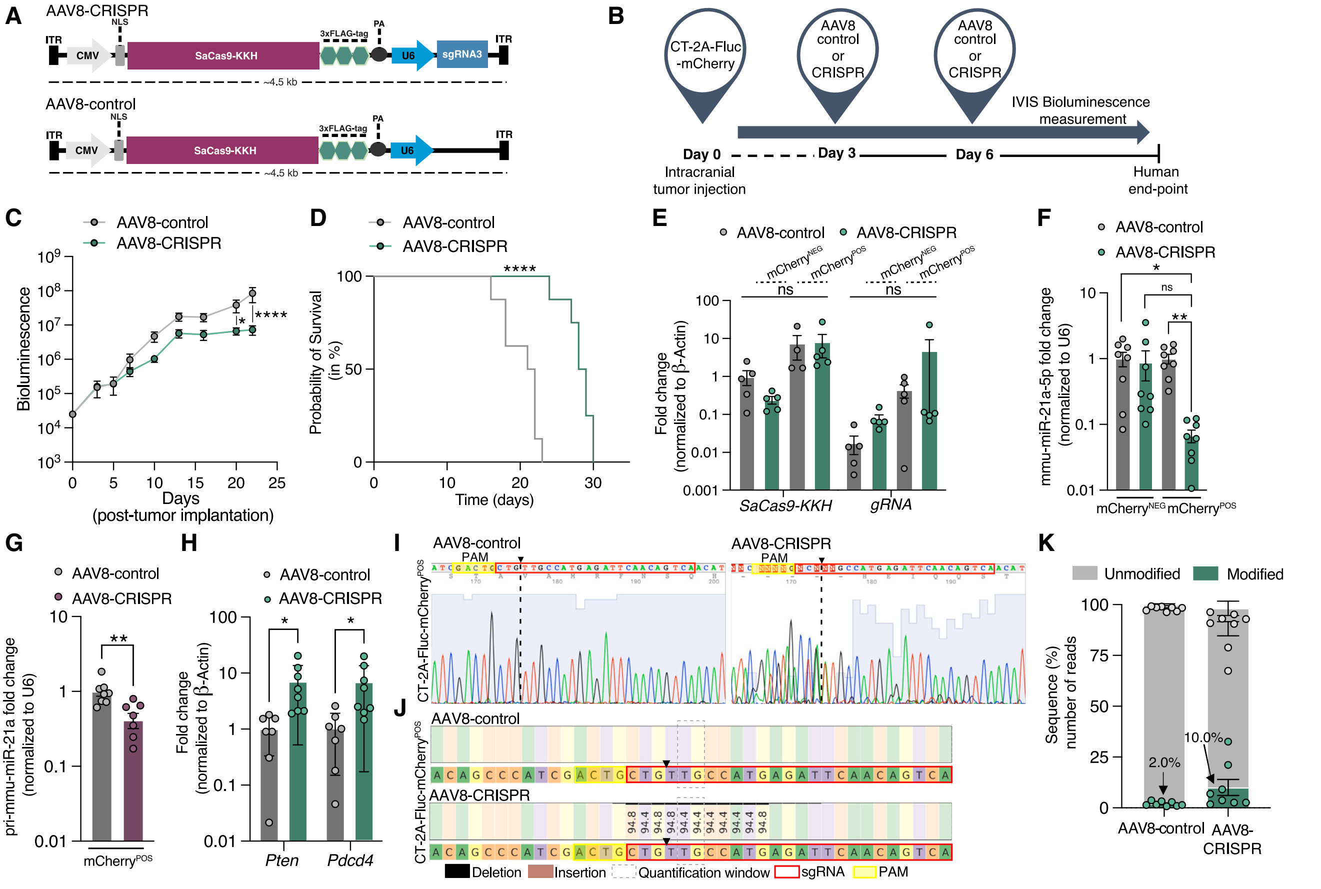

Figure 4. *In vivo* genome editing of mmu-miR-21a by SaCas9-KKH delivered by AAV-8 vector in GB-bearing mice

(A) The AAV8 serotype capsid with a total size of 4.5 kb expressing SaCas9-KKH under a CMV promoter tagged with the 3xFLAG-tag and the sgRNA3 driven by the U6 promoter flanked by ITRs (AAV8-CRISPR) and a control AAV8 vector lacking the sgRNA3 (AAV8-control). (B) Schematic layout of the experimental design treating mice on days 3 and 6 with AAV8-CRISPR or AAV8-control post tumor implantation. (C) Bioluminescence data show a reduction in tumor growth at day 12 post tumor injection in mice injected with the AAV8-CRISPR ($n = 8$) with significantly reduced tumor growth at day 20 ($p = 0.0204$) and day 22 ($p < 0.0001$) post tumor implantation compared to the AAV8-control ($n = 8$). Data are presented as the mean with SEM (error bars). Šídák's multiple comparisons two-way ANOVA, *$p < 0.01$, ****$p < 0.0001$. (D) Kaplan-Meier survival curve after intracranial injection of 50,000 tumor cells (CT-2A) in a total of 10 mice. Eight mice treated with AAV8-CRISPR had a significantly prolonged survival with a median survival of 28.5 days, compared to eight mice treated with AAV8-control with a median survival of 21.5 days. Log rank (Mantel-Cox) test. ****$p < 0.0001$. (E) Fold expression of SaCas9-KKH and sgRNA was measured by RT-qPCR of mice implanted with CT-2A-FLuc-mCherry tumor cells treated with AAV8-CRISPR or AAV8-control at day 3 and 6 post tumor injection; no significant differences were observed between groups. Data represent two independent experiment ($n = 5$ mice) and are presented as the mean with SEM (error bars). Multi-comparison (Tukey) one-way ANOVA; ns, not significant. (F) Eight mice per group were sacrificed on day 16 post tumor implantation and FACS sorted for $\text{mCherry}^{\text{POS}}$ tumor cells and mCherry-negative ($\text{mCherry}^{\text{NEG}}$) non-tumor cell populations. mmu-miR-21a-5p expression analysis showed 15-fold reduction of mmu-miR-21a-5p levels in tumor-bearing mice injected with AAV8-CRISPR compared to the AAV8-control in $\text{mCherry}^{\text{POS}}$ (tumor) cells but not in $\text{mCherry}^{\text{NEG}}$ (non-tumor) cells. Data are presented as the mean with SEM (error bars). Multi-comparison one-way ANOVA, *$p < 0.05$, **$p < 0.01$. (G) Tumor-bearing mice injected with AAV8-CRISPR compared to the AAV8-control in $\text{mCherry}^{\text{POS}}$ (tumor) cells showed a 2.5-fold pri-mmu-miR-21a reduction upon AAV8-CRISPR treatment. Unpaired t test, **$p < 0.01$. (H) mmu-miR-21a downstream mRNA target genes *Pten* and *Pdcd4* were normalized to *β-Actin*. Mice treated with AAV8-CRISPR or AAV8-control in mCherryPOS (tumor) cells were compared. 7-fold upregulation of *Pten* expression levels and 6.6-fold increased *Pdcd4* expression in the AAV-CRISPR-treated condition compared to AAV8-control. Each data point represents a single mouse (AAV8-control, $n = 7$; AAV8-CRISPR, $n = 8$) and data are presented as the mean with SEM (error bars). Unpaired t test and multiple t test, *$p < 0.05$. (I) Representative Sanger sequencing results of PCR products spanning mmu-miR-21a were analyzed using 4Peaks software and show the position of the sgRNA3 (red), the PAM sequence (yellow), and the nicking site (black dashed line) in the mmu-miR-21a reverse-sequence traces in $\text{mCherry}^{\text{POS}}$ cells of tumor-bearing mice i.c. injected with AAV8-CRISPR compared to the AAV8-control. Blue shading behind the peaks represents base quality. AAV8-control shows single-base peaks, while the AAV8-CRISPR-edited cells show double peaks and missing nucleotides (N) due to an indel in the mmu-miR-21a sequence. (J) Indel modifications in the targeted mmu-miR-21a sequence. Nucleotide percentage quantification was performed and showed deletions (black) in the region of interest in the sgRNA3 AAV8-CRISPR-edited sequence compared to AAV8-control. (K) NGS analysis showed that AAV8-CRISPR (10.0%) were modified (green) compared to AAV8-control (2.0%); reads not modified are shown in gray. Data represent eight replicates and are presented as the mean with SEM (error bars). Multi-comparison one-way ANOVA; ns, not significant.

size on day 20 and day 23 (Figure 4C). Interestingly, the mice that received AAV8-CRISPR therapy on days 3 and 6 had a significantly increased median overall survival (28.5 days) compared to the AAV8-control group (21.5 days), with a 31% increase in survival (Figure 4D). Next, to evaluate CRISPR activity, eight mice were sacrificed on day 16 post tumor injection, and ipsilateral hemispheres were FACS sorted for mCherry^{POS} tumor cells and mCherry-negative (mCherry^{NEG}) non-tumor cells. SaCas9-KKH expression was increased 7-fold in both AAV8-CRISPR and AAV8-control of mCherry^{POS} tumor cells compared to mCherry^{NEG} cells. Additionally, the expression of sgRNA was increased 4.4-fold in FACS-sorted mCherry^{POS} tumor cells only in mice treated i.c. with AAV8-CRISPR compared to AAV8-control (Figure 4E). Mmu-miR-21a-5p expression levels were significantly reduced in mice treated with AAV8-CRISPR only in the mCherry^{POS} tumor cells compared to AAV8-control and non-treated CT-2A cells (Figure 4F). Importantly, mmu-miR-21a-5p levels remained unaffected in the livers of mice that received AAV8 therapy intratumorally on days 3 and 6 post tumor cell injection (Figure S6I). The pri-mmu-miR-21a levels were reduced in mCherry^{POS} tumor cells treated with AAV8-CRISPR compared to AAV8-control (Figure 4G). Downstream mmu-miR-21a-5p mRNA targets, *Pten*, and programmed cell death protein 4 (*Pdcd4*) were analyzed using RT-qPCR analysis in mice treated with AAV8 on day 3 and day 6 post tumor implantations.[49,50] We found that the tumor suppressor genes *Pten* and *Pdcd4* were significantly upregulated by 7.9-fold and 6.6-fold, respectively, in mice treated with AAV8-CRISPR in mCherry^{POS} tumor cells compared to AAV8-control (Figure 4H). *Btg2* and SKI proto-oncogene (*Ski*) are other known downstream targets of mmu-miR-21a.[51,52] These downstream mRNA targets showed an upward trend, but they were not significant (Figure S7A). We further evaluated whether administration of treatment on days 5 and 10 would have a therapeutic effect (Figure S7C). However, no differences in bioluminescence signal were observed between mice that were treated with AAV8-CRISPR compared to AAV8-control on day 5 and day 10 (Figure S7D), indicating that this therapy may be time sensitive requiring administration within a specific time window. Mice that were i.c. treated with AAV8-CRISPR or AAV8-control at day 5 and day 10 post tumor implantation showed elevated SaCas-KKH expression in all AAV8-injected mice compared to non-treated CT-2A WT. Additionally, gRNA expression was 10.2-fold (mCherry^{POS}) or 13.6-fold (mCherry^{NEG}) increased in mice that were treated i.c. with AAV8-CRISPR compared to AAV8-control (Figure S7E). Mmu-miR-21a-5p expression levels were 32-fold downregulated in mCherry^{POS} tumor cells of mice that were treated with AAV8-CRISPR compared to CT-2A WT cells on day 5 and day 10 post tumor injection (Figure S7F). In mice treated with AAV8 on day 5 and day 10 post tumor implantation, no differences were observed in downstream mmu-miR-21a-5p mRNA targets (Figure S7G). Next, mCherry^{POS} tumor cells were analyzed using Sanger sequencing. The AAV8-CRISPR-edited mmu-miR-21a genome sequence was aligned with the AAV8-control sequence, revealing CRISPR-induced indels were observed (representative Sanger results shown) (Figure 4I). In mCherry^{NEG} (non-tumor) cells, no disruption of the mmu-miR-21a sequence was detected (Figure S7B). CRISPR-edited cells showed a deletion around the nicking site compared to AAV8-control (Figure 4J). NGS results showed that 10.0% of the reads were modified after gene editing with AAV8-CRISPR, compared to 2.0% in mice injected with AAV8-control (Figure 4K). In contrast, marginally modified reads (AAV-CRISPR ∼6%; AAV-SaCas9-control 1%) (data not shown) were observed in the other tested experimental setups (tumor and AAV8 vectors injected at the same time point and AAV8 vector injections at day 5 and day 10 post tumor implantation) (Figures S6B and S7C), which was expected because both experiments did not show any difference in survival outcomes between groups (data not shown). To study local toxicity of AAV injection, we performed terminal deoxynucleotidyl transferase dUTP nick end labeling (TUNEL) staining (Figure S8A), and this showed no AAV8-induced local toxicity. In addition, no gliosis or activated microglia were observed in the left ipsilateral striatum upon AAV8 injection based on GFAP and IBA1 immunostaining (Figure S8B). To assess potential systemic toxicity, blood pathology was evaluated by analysis of multiple blood markers such as albumin, alkaline phosphatase (ALP), alanine transaminase (ALT), blood urea nitrogen (BUN), calcium, cholesterol, creatine, globulin, glucose, phosphorus, total bilirubin, and total protein. None showed significantly elevated levels, suggesting no systemic toxicity for either AAV8-CRISPR and AAV8-control-injected mice (Figure S8C). Taken together, we showed that SaCas9-KKH/sgRNA-mediated gene editing delivered through an AAV8 vector significantly downregulated mmu-miR-21a levels in GB-bearing mice, reduced tumor growth, and increased overall survival. Moreover, the provided data demonstrate that safe *in vivo* gene editing through gene-therapy delivery using a single AAV vector caused a reduction of mmu-miR-21a-5p in the brains of CT-2A GB-bearing mice.

DISCUSSION

In this study, we designed a single AAV vector containing SaCas9-KKH guided by an sgRNA to target mmu-miR-21a coding sequences in mouse GB cells and the surrounding tumor microenvironment. This therapeutic vector resulted from screening 11 different sgRNAs through co-transfection with SaCas9-KKH and selecting the most efficient sgRNA in downregulating mmu-miR-21a expression levels *in vitro*. Knockout was confirmed at the genomic level by Sanger sequencing and NGS. Next, we tested our selected sgRNA in an all-in-one construct where gene editing was validated by reduced levels of mmu-miR-21a, inhibited proliferation rates of GB cells, and deletions in the mmu-miR-21a locus. Delivery of CRISPR-SaCas9 nucleases by AAV vectors[16] has shown promising results in pre-clinical models of various diseases,[53–59] including GB.[60,61] However, the episomal nature of the recombinant AAV vectors makes it sensitive to dilution in cell division, thus creating a challenge in transducing rapidly dividing cancer cells. To deliver the CRISPR nucleases *in vivo*, an AAV8 capsid with previously reported transduction of GB cells in the CNS[21,39,40,62] was used. As previously demonstrated,[63] we observed an intense layer of transduced cells surrounding the tumor with the AAV8-GFP vector. Direct fluorescence of tumor sections revealed the presence of transduced GFP^{POS} cells that did not colocalize

with tumor-expressed mCherry. These are likely neurons, which are more differentiated than the tumor cells and thus less susceptible to loss of transduction due to dilution of AAV genomes.[64] We only detected 3.4% of tumor cells transduced 1 week post transduction. This is presumably a vast underestimate of the extent of transduction. First, tumor cells divide rapidly and dilute out the episomally maintained AAV vector.[48] Second, AAV vectors can mediate long-term as well as short-term transduction events that are not detected by fluorescence reporters.[65] Evidence for this is our genome-editing data, which showed that ∼10% of tumor cells were edited, while we only detected 3.4% of GFP^{POS} tumor cells in the transduction experiment, a ∼3-fold difference. This is important information for the development of genome-editing strategies delivered by AAV vectors, as even short-term, low-level expression of SaCas9-KKH enzyme can lead to permanent genetic modifications, which separate this strategy from gene addition strategies, where effective therapy often relies on stable expression from the AAV concatemer.[66] The all-in-one CMV-SaCas9-KKH-U6-sgRNA3 cassette was packaged into a single AAV8 capsid, and we demonstrated targeting of mmu-mir-21a locus using this vector in a GB-bearing mouse model. We found that the tumor suppressor genes *Pten* and *Pdcd4*, downstream targets of mmu-miR-21a-5p, were upregulated in CT-2A tumor-bearing mice when mmu-miR-21a was knocked out in AAV8-CRISPR-treated mice. *Pten*, frequently suppressed in GB, is a validated target of mmu-miR-21a-5p,[67] and it has been previously reported that inhibition of miR-21 in GB tumors increases *Pten*.[68] The molecular process prior to gene suppression by miRNAs starts with the miRNAs being transcribed as pri-miRNAs, followed by processing by Drosha/DGCR8 to pre-miRNA. After being exported out of the nucleus, pre-miRNAs are further processed by TRBP/Dicer into mature miRNAs. After this, only one arm of the miRNA is integrated into the miRISC complex. This complex is responsible for binding target mRNAs followed by deadenylation and degradation, decapping, and further degradation or ribosomal drop-off. The remaining arm, not integrated into the complex, is mostly degraded. Based on sequencing data and miRNA expression data, we find that the mmu-miR21a-5p is present at high levels in GB while the mmu-miR-21a-3p is only detectable at low levels. We hypothesize that editing the mmu-mir-21a locus does not affect the targets of mmu-miR-21a-3p, since this form of the miRNA is only expressed at low levels in unedited cells, and its expression levels remain unchanged after mmu-mir-21a locus editing.

Excitingly, the number of clinical trials that study US Food and Drug Administration (FDA)-approved small RNAs as diagnostic biomarkers or therapeutics has been increasing in recent years.[69–71] RG-012, a phosphorothioate, 2′-O-methoxyethoxy modified antagomiR targeting miR-21, has been tested in a phase I clinical trial in patients with Alport syndrome (NCT03373786). Although the role of miR-21 in GB has been well understood and extensively reviewed,[11] the translation of this knowledge into clinical applications is still evolving. Multiple pre-clinical studies have tried to inhibit miR-21 levels by using xenograft mouse models to reflect clinical tumors more closely. For example, anti-miR-21 oligonucleotides injected directly into the cortex showing satisfactory knockdown of miR-21.[72] Suppression of miR-21 using locked nucleic acid-anti-miR-21 oligonucleotides, combined with the injection of engineered neuronal precursor cells expressing a secretable form of cytotoxic TRAIL (sTRAIL) resulted in a reduced GB volume in mice.[73] Nanoparticles have been used as a different approach to deliver anti-miR-21 sequences to suppress GB growth *in vivo*.[67,74] More recently, anti-seed peptide nucleic acids targeting the seed region of onco-miRNAs including miR-21(a) were delivered through nanoparticles in human GB xenograft mouse models and prolonged survival outcomes.[75] Reduction in tumor growth and improved survival have also been observed when injecting GB cells completely lacking miR-21(a).[35] Although studies that used antisense oligonucleotide inhibitors or miRNA sponges to reduce miR-21(a) expression have shown promising results, the affinity of these inhibitors is weak due to the short length of miRNAs.[70] CRISPR-Cas9 nuclease genomic knockout is a more robust approach with the advantage of high efficiency, specificity, and permanent disruption of the genomic source of miR21(a).

In recent years, CRISPR-Cas9 nucleases have been widely used in the GB field as summarized.[76] Attempts have been made to disrupt various potential therapeutical targets with CRISPR technology[77] in GB, such as EGFR,[78] IDH-1,[79] PD-L1,[80] TIM-3,[81] and CD44.[82] Realizing the full potential of CRISPR-based genome editing requires an efficient gene-transfer delivery strategy. Various approaches to deliver nucleases into the brain have recently been developed, including nanocapsules capable of penetrating the blood-brain barrier. These nanocapsules were utilized to target serine/threonine protein kinase (PLK1) non-invasively in GB.[83] Additionally, the delivery of nucleases through RNPs for genome editing in brains has been explored.[84] A variety of gene transfer platforms have been developed previously and have been applied in cancer disease models, including viral vectors, such as adenovirus, herpes simplex virus, and AAV vectors.[85] Among these, AAV-based gene therapy has been extensively tested in experimental GB models.[19] Currently, AAV vectors are preferred due to their targetability, low toxicity, and limited immune response.[86] With the recent FDA approval of AAV vectors for clinical use, this delivery platform offers multiple advantages, such as a relatively good safety profile and versatile manufacturing processes.[18] However, AAV vectors have been reported to cause dose-limiting toxicity in patients.[87] Simultaneous delivery of multiple vectors is limited by the total AAV vector dose, raising additional safety concerns. Moreover, the long-term expression of SaCas9-KKH mediated by AAV vectors is prone to increase off-target editing and can trigger an immune response from the host due to the bacterial origin of SaCas9-KKH. Non-viral delivery methods are potentially a safer option that offer a limited expression of SaCas9-KKH in the target tissue.[88] Due to the compact coding size of the SaCas9-KKH variant, we were able to deliver SaCas9-KKH and the corresponding sgRNA in an all-in-one vector, thereby minimizing the invasiveness of the treatment and lowering the dosage of the AAV vector necessary for the therapeutic outcome. This ultimately reduces toxicity and off-target side effects compared to dual AAV vector strategies, where AAV vectors are needed to deliver a split version of bigger nucleases,

such as *Streptococcus pyogenes* Cas9 (SpCas9). The transduction efficiency of specific cell types by AAV vectors depends on the AAV serotype, administration route, and target tissues. Previously, it has been shown that AAV8 can transduce GB cells.[40] Others have shown that screening for specific AAV capsids can increase the specificity of the AAV transduction of specific cell types.[89] Here, we used the established AAV8 serotype as a delivery vector for our genome-editing strategy. We found that edited tumor cells had downregulation of mmu-miR-21a, subsequent reduction in tumor growth, and an increase in overall survival. However, the strategy of delivering SaCas9-KKH together with the sgRNA could be further developed using more advanced delivery modalities such as lipid nanoparticles, extracellular contractile injection systems (eCISs),[90] or more efficient AAV capsids.[91] Future research should be conducted to optimize the delivery of the gene-editing reagent to GB cells to increase the specificity and decrease the potential off-target effect in non-tumor cells. For example, future studies could aim to optimize the AAV capsids,[21] develop AAV vectors with advanced tropism for the CNS,[92] and improve tumor specificity. The latter could be accomplished by adjusting the promoter driving the SaCas9-KKH nuclease expression, such as using the Ki-67 promoter, which is active in dividing cells and would confer specificity to targeting GB cells.[93] Finally, therapeutic potential could be improved by using different routes of administration for the AAV vector to avoid the invasive nature of intratumoral administration.[94] Recently, a gelatin-based AAV-delivering approach was developed to enhance local delivery and AAV expression in brain tumors.[95] This innovative approach could potentially complement our AAV-based therapeutic strategy.

GBs are known for their complex genetic heterogeneity, a hallmark that is correlated with resistance to currently available therapies. Our therapeutic strategy uses AAV8 to deliver saCas9 into the tumor cells and tumor microenvironment. Although we used a capsid that has been shown to mediate the highest level of transduced tumor cells in the brain, we showed that, despite injecting the AAV vector intratumorally, most of the cells that are transduced are located at the tumor periphery, indicating that the AAV vector has difficulties penetrating the tumor core. We further hypothesize that, within the GB, some tumor cells with a certain genetic profile are more susceptible to AAV transduction, while other tumor cell populations could be more therapy resistant. This intertumoral heterogeneity could affect the efficiency of the therapy. Improved AAV capsids and the use of more specific promoters that are currently being developed might improve delivery efficiency to tumor cells. Moreover, different delivery approaches, such as RNPs,[88,93] or combined therapeutic approaches with multiple modes of administration could potentially increase the number of targeted tumor cells with different phenotypic profiles. Another degree of heterogeneity important for our therapeutic outcome is the expression levels of miR-21(a) within the tumor. We do not know if every cell within the tumor has similar levels of miR-21(a) expression. We hypothesize that targeting cells with high levels of miR-21(a) would likely reduce cell proliferation and tumor growth as compared to targeting cells that express lower miR-21(a) levels. Moreover, we should highlight that GB mouse models do not reflect the degree of heterogeneity of human GBs. Further studies are therefore needed to test our AAV approach in patient-derived tumor models and screen its efficiency across the patient population. Additionally, GB is known for its heterogeneity among patients, and meta-analysis studies suggest that high levels of miR-21 are correlated with poor patient survival outcomes and resistance to therapy.[10,94] Our therapeutic approach would therefore be more beneficial for patients with high levels of miR-21. Hypothetically, downregulation of miR-21(a) would not only improve overall survival but also allow other therapies such as chemotherapy and radiotherapy to be more effective in combination with miR-21(a) downregulation.

To the best of our knowledge, miR-21 has never been targeted *in vivo* through the delivery of an AAV vector CRISPR-based therapy. This all-in-one AAV-SaCas9-KKH-sgRNA approach specifically targeting miRNA coding sequences *in vivo* can be applied not only in the context of GB but also in other cancers with abundant miR-21 expression, such as breast, lung, and colorectal tumors[96] and for a range of genome-editing purposes *in vivo*. This study shows effective genome editing of mmu-miR-21a through AAV-mediated delivery of CRISPR-SaCas9-KKH in a GB mouse model as a proof of concept. Further studies will focus on applying this approach in human GB xenograft mouse models to support the clinical translation of this method. While this approach does not entirely stop the tumor progression, it has a significant effect on halting tumor growth. Since treatment options for GB are currently limited, any alternative therapy should be beneficial. This genetic approach has the potency to use different delivery modes and could assist in the development of a more targeted treatment. However, we should acknowledge the social considerations when utilizing gene-editing tools, as they might elicit some public concerns. Fortunately, CRISPR-based therapies are currently approved by the FDA (e.g., sickle cell disease), which will aid in introducing and normalizing these new types of CRISPR-based treatments to the public. Legally, the use of CRISPR in patients is now being tested and approved. However, ethical issues remain when using these gene-editing tools, and potential off-target effects could potentially cause long-term, detrimental effects. Ultimately, in a currently uncurable disease like GB, any improvement in the therapeutic options should be considered, and the potential side effects of this therapy would be limited to the treated patient. Future clinical trials should evaluate whether the anticipated prolonged survival outcomes outweigh potential therapeutical side effects and whether such concerns are encompassed in current safety standards. In addition to specifically targeting miR-21(a), this strategy could be applied to target any miRNA by designing sgRNAs targeting the coding sequences of the miRNA of interest. Using Cas9 to target miRNAs offers the advantage of completely knocking out miRNA with a single cut, whereas targeting genes with CRISPR might still produce truncated proteins with potentially negative effects. Additionally, this system allows for the multiplexing of multiple sgRNAs simultaneously,[97] which can be beneficial for complex and polygenetic diseases like GB that involve multiple loci. Furthermore, our AAV-mediated gene therapy might be compatible with other anti-GB therapies. More importantly, miR-21(a) has been reported to be associated with resistance to chemotherapies in cancers such as GB.[98] The

downregulation of miR-21(a) achieved by our gene therapy could therefore be a promising strategy to reverse chemoresistance.[99] The engineering of a single AAV vector can achieve multiple therapeutic genome-editing outcomes. However, in our experimental setup, tumor cells did regrow, likely because we did not transduce all tumor cells. The heterogeneity of effects in different cells can be associated with different tropisms of the AAV vector serotypes and different chromatin accessibility to allow the mmu-mir-21a locus to be edited. Future studies should focus on targeting different miRNAs in the stromal cells located at the tumor border since these cells present a more stable reservoir for AAV transduction.[100] Previously, it has been shown that high levels of mmu-miR-21a in microglia located at the tumor periphery create a favorable microenvironment for GB progression.[101] Hence, the knockout of the miR-21a locus in cells within the tumor microenvironment could potentially reduce tumor growth. In summary, our approach shows the potency of a single AAV vector delivering SaCas9-KKH/sgRNA for therapeutic genome editing in GB.

Conclusions

We utilized CRISPR-SaCas9/sgRNA targeting mmu-miR-21a using AAV-based gene therapy in GB mouse models. Using an all-in-one AAV-sgRNA-SaCas9-KKH vector approach, we demonstrated *in vivo* editing in the context of GB that can potentially be extended for other genome-editing purposes. We anticipate that successful *in vivo* delivery of an accurate and effective SaCas9 could contribute to therapeutic gene editing in humans.

MATERIALS AND METHODS

Animals

All animal experiments were conducted under the oversight of the Massachusetts General Hospital Institution Animal Care and Use Committee (IACUC) and all experiments were performed conform to all relevant regulatory standards. C57BL/6J from Charles River Labs (IACUC protocol 2009N000054) were maintained with unlimited access to water and food under a 12-h/12-h light/dark cycle. To study tumor proliferation *in vivo*, we used a total of ~60 C57BL/6J adult male mice randomly assigned to each group.

Cells

Mouse CT-2A and GL261 GB cell lines were provided by The National Cancer Institute (NCI). CT-2A cells were cultured in a 5% CO_2 humidified incubator at 37°C in Dulbecco's modified Eagle's medium (DMEM; Corning, New York, NY) with 10% fetal bovine serum (FBS; GeminiBio, West Sacramento, CA) and 1% penicillin (100 units/mL)/streptomycin (100 mg/mL) (p/s). GL261 cells were cultured in Roswell Park Memorial Institute (RPMI) (Corning) with 10% FBS and 1% p/s. Cells were checked regularly for mycoplasma infections using the PCR Mycoplasma Detection Kit (G238; ABM, New York, NY).

Intracranial tumor or vector injection

Adult mice were anesthetized using 2.5% isoflurane in 100% oxygen via a nose cone. A total of 1×10^5 CT-2A-mCherry cells stably expressing FLuc were suspended in 2 μL of Opti-MEM (Gibco, Waltham, MA). The cells and/or AAV vectors (2×10^{10} genome copies [gc]/μL per mouse) in a total volume of 2 μL were then i.c. injected into the left striatum of C57BL/6J mice using a Hamilton syringe (Sigma-Aldrich, St. Louis, MO) and automatic stereotaxic injector (Stoelting, Wood Dale, IL) with a flow rate of 0.2 mL/min. In reference to bregma, three coordinates for stereotactic implantation were chosen: anterior-posterior (AP) = 2.0 mm, medial-lateral (ML) = 0.5 mm, and dorsal-ventral (DV) = 2.5 mm. The human endpoint was determined based on 20% weight loss or if mice were under clear distress or their actual death. Tumor growth in mice was followed by measuring bioluminescence using IVIS (PerkinElmer, Waltham, MA).

IVIS

IVIS (PerkinElmer) was used to monitor bioluminescence as a measure of tumor growth. To perform IVIS measurement, mice were anesthetized by a continuous flow of 2.5% isoflurane (Baxter, Deerfield, IL) in 100% oxygen. They were intraperitoneally (i.p.) injected with D-Luciferin sodium salt (5 mg per animal) (Gold Biotechnology, Olivette, MI), incubated for 5 min, and scanned with an exposure time of 3 min using IVIS.

Whole-blood collection

Mice were sacrificed by a lethal i.p. injection of 100 μL containing ketamine (5 μL), xylazine (45 μL), and saline (50 μL) (Patterson Veterinary, Hanover, MA). Upon ceasing of all reflexes, 500 μL of whole blood was collected directly from the heart into Eppendorf tubes or in EDTA anticoagulant-coated tubes (UKmedi, the Netherlands). Whole blood and serum were analyzed at the Pathology Core at Massachusetts General Hospital (MGH) using the comprehensive blood toxicology panel and complete blood count (CBC) panel. C-reative protein (CRP) levels were analyzed at Idexx BioAnalytics (West Sacramento, CA).

Tissue digestion

Tumor-bearing mice (CT-2A-mCherry) were exsanguinated with PBS perfusion after i.p. injection of a mixture of ketamine (Patterson Veterinary) and xylazine (Patterson Veterinary). Tumor Tissue Dissociation Kit (Miltenyi Biotec, Charlestown, MA) was used to process the brain into a single-cell suspension. The ipsilateral hemisphere of the brains was placed into a GentleMacs C-tube (Miltenyi Biotec) with 2.35 mL of RPMI 1640 containing enzymes D (100 μL), R (30 μL), and A (3.5 μL) and incubated for 40 min at 37°C. According to the manufacturer's protocol, the brains were dissociated using the gentle MACS Dissociator (Miltenyi Biotec). Samples were run through a 70-μm filter to obtain a single-cell suspension. Myelin removal was achieved using magnetic separation and anti-myelin beads (Miltenyi Biotec). The final cell suspension was resuspended in 1× Dulbecco's (D) PBS without calcium (Ca^{2+}) or magnesium (Mg^{2+}) (Corning), supplemented with 2 mM EDTA (Thermo Fisher Scientific, Waltham, MA) and 0.5% BSA (Sigma-Aldrich). Single cells were further processed using FACS to sort mCherry^{POS} tumor cells to analyze CRISPR efficiency post AAV vector treatment.

Cloning

The pUC19 plasmid (obtained from Dr. Kleinstiver, MGH) was used as the vector backbone. sgRNAs were cloned into the entry vector backbone by digesting the plasmid with BsmBI-v2 restriction enzyme (catalog: R0739; New England Biolabs, Ipswich, MA). In total, 11 sgRNAs, each targeting a specific part of the mmu-miR-21a sequence, were cloned. This resulted in 11 plasmids, each containing a unique sgRNA, expressed under a U6-promoter. sgRNA top (10 μM) and bottom (10 μM) oligos (Table S3) were annealed in T4 DNA ligase buffer (New England Biolabs (NEB)) at 95°C for 5 min and then cooled to 10°C at −5°C per min, using the C1000 Touch Thermal Cycler (Bio-Rad, Hercules, CA). To digest the pUC19 restriction enzyme BsmBI-v2 and 10× NEB 3.1 buffer were used. The samples were incubated at 37°C for 16 h. The plasmids were isolated using the Qiagen gel extraction kit. The digested plasmid was ligated with 0.5 μM oligo duplex and 10× T4 DNA ligase buffer (New England Biolabs) pre-warmed at 42°C for 5 min prior to use at 24°C for 10 min. Successful cloning was determined by Sanger sequencing using primers oS280 pUC19 (Table S4).

All-in-one plasmid constructs and AAV production

The SaCas9-KKH expression construct was amplified using a primer set amplifying 2,893 bp (forward, 5′cggtaggcgtgtacggtgggaggtctatat aagcagagctCCGGTCGCCACCATGGGGAAACGGAACTACATCC TGGGGCTTGACATTGGGATAACCAGC′3 and reverse, 5′GCA CTTTGAGTTTACTTCATAATAGTTCTCCTTTTTGATC′3) and a 500-bp G-block was designed containing sgRNA3 and the 3xFLAG-tag. All fragments were cloned between the ITRs (4,063 bp) of the pX601-AAV-CMV::NLS-SaCas9-NLS-3xHA-bGHpA; U6::BsaI-sgRNA plasmid (Addgene catalog: 61591; https://www.addgene.org/61591/)[15] after a digest using the restriction enzymes SmaI (catalog: R0141; New England Biolabs) and HindIII (catalog: R0104; New England Biolabs) followed by Gibson assembly with NEBuilder HiFi DNA Assembly Master Mix (New England Biolabs) at 50°C for 2 h. Both the CMV-SaCas9-KKH-U6-sgRNA3 and the CMV-SaCas9-KKH-U6 cassettes were then transformed into SURE Electroporation Competent cells (Agilent Technologies, Lexington, MA) by two pulses at 1,700 V. Plasmid DNA was isolated in nuclease-free water (Ambion Life Technologies, Carlsbad, CA) using the Qiaprep Spin miniprep kit (Qiagen, Hilden, Germany) after selection with 1 μg/mL ampicillin (ampicillin sodium salt, Sigma-Aldrich). Both plasmid constructs were fully sequenced with NGS at the MGH CCIB DNA core and analyzed with Snapgene software version 6.0.2. Upon confirmation of the sequence, the plasmid constructs were isolated at a large scale by AltaBiotech at a concentration of 2 μg/μL. Subsequently, both CMV-saCas9-U6-sgRNA3 and the CMV-saCas9-U6 cassettes were packaged into the AAV8 serotype capsids and both vectors were produced by Packgene at a titer of 1.0×10^{13} gc/mL.

Cell transfection

CT-2A and GL261 cell lines were transfected using Lipofectamine 2000 (Thermo Fisher Scientific) in Opti-MEM utilizing the manufacturer's protocol. More specifically, 8×10^5 GB cells were plated onto a six-well plate (Corning). Transfection mixes consisting of the SaCas9-KKH plasmid (400 ng) and/or sgRNA (1–11)-expressing plasmids (pUC19, 100 ng) or the all-in-one construct (400 ng) were incubated at room temperature (RT) for 20 min before adding to the six-well plate. Cells were incubated with the transfection mix for 72 h at 37°C. The transfection efficiency was monitored by the level of fluorescence using a ZOE Fluorescent Cell Imager (Bio-Rad).

FACS

To test the editing efficiency of the different sgRNAs, cells were sorted for GFP^{POS} in bulk after co-transfection with SaCas9-KKH and pUC19 plasmids. Cells transfected with the all-in-one vector were bulk sorted for GFP. Cells transfected and incubated for 72 h were stained with 40,6-diamidino-2-phenylindole (DAPI; Invitrogen, Carlsbad, CA) at final concentration of 1 mg/mL. Live cells were sorted by selecting only DAPI-negative cells using a BD FACSAria II SORP Cell Sorter (BD Biosciences). After sorting, cells were directly plated in 96-well plates to measure proliferation rates, pelleted for DNA extraction and sequencing, or sorted directly in QIAzol lysis buffer to extract total RNA with the Direct-Zol MicroRNA kit (Zymo Research, Irvine, CA). RNA concentrations were measured using the Nanodrop Spectrophotometer ND-1000 (Thermo Fisher Scientific).

For *in vivo* experiments, cells from the ipsilateral hemisphere of the brains were FACS sorted for $mCherry^{POS}$ tumor cells and $mCherry^{NEG}$ non-tumor cells after tissue digestion. Cells were sorted directly into QIAzol lysis buffer to extract total RNA with miRNeasy Micro Kit (Qiagen). RNA concentrations were measured using the Nanodrop Spectrophotometer ND-1000 (Thermo Fisher Scientific). Tumor-bearing brain samples were analyzed for mmu-miR-21a expression levels, downstream mRNA targets, and DNA sequencing to determine on-target gene-editing events.

In vivo genomic DNA extraction, NGS amplicon sequencing, and data analysis

Genomic (g)DNA was extracted using the adapted TRIzol Reagent Experimental protocol for DNA isolation (Invitrogen, 15596026), from FACS-sorted $mCherry^{POS}$ (tumor cells) and mCherryNEG (non-tumor cells) by utilizing the remaining sample post removal of the aqueous layer of the QIAzol lysed RNA (Qiagen). 300 μL of 100% isopropanol was added to the sample followed by a 30-min incubation at RT. To pellet the DNA, the sample was centrifuged for 5 min at 2,000 × *g* at 4°C. The DNA pellet was resuspended in 1 mL of 0.1 M sodium citrate in 10% ethanol incubated for 30 min and pelleted at 2,000 × *g* for 5 min at 4°C. Next, the pellet was washed in 1 mL of 75% ethanol and incubated for 10–20 min. Following 5-min centrifugation at 2,000 × *g* at 4°C, the pellet was air-dried and resuspended in H_2O. The DNA sample was then heated for 6 min at 66°C and heated to 95°C for 2 min. Following incubation, gDNA was purified using a 0.8× ratio of paramagnetic beads (speed-bead magnetic carboxylate modified particles, 15 mL, azide 0.05%). Genome-modified reads were determined by NGS using a two-step PCR-based Illumina library construction method. The first

PCR reaction was donewith approximately 100 ng of gDNA using Q5 High-fidelity DNA Polymerase (NEB) and the respective primers (forward: ACACTCTTTCCCTACACGACGCTCTTCCGATCTTT GACTGCAAACCATGATGCTG and reverse: GACTGGAGTTCAG ACGTGTGCTCTTCCGATCTTGCTTTAAACCCTGCCTGAGC), with cycling conditions of one cycle at 98°C for 2 min; 35 cycles of 98°C for 10 s, 65°C for 10 s, 72°C for 20 s; and one cycle of 72°C for 1 min. PCR-1 products were purified using paramagnetic beads at a ratio of 1.8×. Approximately 20 ng of purified PCR-1 product was used as a template for a second PCR (PCR-2) to add Illumina barcodes with adaptor sequences using Q5 (cycling conditions of one cycle at 98°C for 2 min; 10 cycles at 98°C for 10 s, 65°C for 30 s, 72°C for 30 s; and one cycle at 72°C for 5 min). PCR-2 products were pooled based on concentrations from capillary electrophoresis (QIAxcel, Qiagen). Final libraries were quantified by Qubit dsDNA High Sensitivity assay (Thermo Fisher) and sequenced on a MiSeq sequencer using a 300-cycle v2 kit (Illumina). On-target genome-editing activities were determined from amplicon sequencing data using CRISPResso2 (http://www.crispresso2.pinellolab.org).[102]

In vitro genomic DNA extraction, Sanger sequencing, and NGS

After transfection, cells were pelleted and gDNA was extracted using the DNAeasy extraction kit (Qiagen) and quantified using a NanoDrop (Wilmington, DE) ND-1000. First, the mmu-miR-21a alleles were amplified, using the Phusion High-Fidelity PCR kit (New England Biolabs) with two different primer sets specifically designed to flank the mmu-miR-21a target sequences. Primers were designed using primer-blast (http://www.ncbi.nlm.nih.gov/tools/primer-blast). For Sanger sequencing, specific primers amplified a DNA fragment of 625 bp with an melting temperature (Tm) of 60°C and, for NGS analysis, primers amplified a 222-bp fragment at a Tm of 67°C (Table S4). The PCR products were purified using the Qiagen PCR purification kit (Qiagen). Next, the PCR products were loaded on a 2% agarose gel (Bio-Research, Worcester, MA), and DNA fragments were resolved by gel electrophoresis. The purified PCR products were sequenced using both Sanger sequencing and NGS analysis at the MGH CCIB DNA Core. CRISPR sequencing was performed by the CCIB DNA Core Facility at MGH (Cambridge, MA). Illumina compatible adapters with unique barcodes were ligated onto each sample during library construction. Libraries were pooled in equimolar concentrations for multiplexed sequencing on the Illumina MiSeq platform with 2 × 150 run parameters. Upon completion of the NGS run, data are analyzed, demultiplexed, and subsequently entered in an automated *de novo* assembly pipeline, UltraCycler v1.0 (B. Seed and H. Wang, personal communication). CRISPR efficiency was determined based on CRISPResso2 analysis (http://www.crispresso2.pinellolab.org).[102]

Flow cytometry

CT-2A-mCherry tumor-bearing hemispheres injected with AAV8-GFP (6.24 × 10^{10} gc/µL) in a total volume of 2 µL on day 7 post tumor implantation were made into single-cell suspensions following the tissue digestion protocol. Single-cell suspensions were then incubated for 10 min on ice in FACS buffer DPBS without Ca^{2+} or Mg^{2+} and 0.5% BSA supplemented with TruStain fcX (anti-mouse CD16/32, BioLegend, San Diego, CA #101319, clone 93, 1:100). Cells were stained for 10 min with LIVE/DEAD Fixable Blue Dead Cell Stain Kit for UV excitation (Invitrogen #L23105, 1:300). Finally, cells were washed with 1 mL of DPBS (without Ca^{2+} or Mg^{2+}) with 0.5% BSA and 2 mM EDTA and were centrifuged at 300 × *g* for 10 min, resuspended in 0.5% BSA, 2 mM EDTA in DPBS (without Ca^{2+} or Mg^{2+}), and passed through a 35-mm nylon mesh strainer (BD Falcon, Franklin Lakes, NJ). Percentages of $mCherry^{POS}$, GFP^{POS}, and double-positive cell populations were measured by SORP 5 Laser BD LSRFortessa X-20 and analyzed by FlowJo (https://www.flowjo.com).

RNA extraction of organs

Lysis buffer was added to the freshly harvested tissue (lungs, spleens, livers, brains). Stainless steel 3-mm tungsten carbide beads (Qiagen) were added, and tissue was lysed and homogenized using the TissueLyser system (Qiagen) for 3 min at 0.25 Hz. RNA was extracted following manufactures' instructions using the Direct-Zol MicroRNA kit (Zymo Research).

Real-time qPCR

RNA was isolated from cell pellets that were lysed in 700 µL of QIAzol lysis reagent (Qiagen) after 72-h transfection or after FACS of brain tissue using the miRNeasy micro kit (Qiagen), and DNase treatment was performed for 15 min at RT according to manufacturer's guidelines. RNA was quantified using a NanoDrop (Wilmington, DE) and converted into cDNA immediately from fresh RNA samples. First, cDNA was produced using the TaqMan MicroRNA Reverse Transcription Kit using 5 ng of total RNA in a volume of 5 µL per reaction (Applied Biosystems, Foster City, CA). Reverse transcriptase reaction master mix contains 100 nM deoxyribonucleotide triphosphates (dNTPs) (with deoxythymidine triphosphate (dTTP)), MultiScribe reverse transcriptase, 50 U/µL, 10× reverse transcription buffer, RNase inhibitor, 20 U/µL, and nuclease-free water. Each reaction contains 7 µL of the master mix, 5 µL of RNA, and 3 µL (5× RT primer working solution) of TaqMan probe in a 15-µL total reaction (Applied Biosystems, Foster City, CA). Cycling settings were 95°C for 20 s, 40 cycles at 95°C for 1 s, and 60°C for 20 s cDNA samples were frozen in −20°C or immediately used for RT-qPCR performance. miRNA levels of the mmu-miR-21a-5p or mmu-miR-21a-3p were analyzed using the TaqMan microRNA assay. RT-qPCR was performed in triplicate using TaqMan Fast Advanced Master Mix (Applied Biosystems). 1 ng of cDNA in 1-µL volume was used per reaction, mmu-miR-21a-5p (000397; Thermo Fisher), mmu-miR-21a-3p (002493, Thermo Fisher), and U6 (001973; Thermo Fisher) primers were used (Table S5) following the manufacturer's protocol. Mmu-miR21a-pri expression was analyzed using TaqMan assay (Mm03306822_pri). cDNA was synthesized from 200 ng of total RNA in a 20-µL reaction volume and prepared using the SuperScript VILO cDNA Synthesis kit (Invitrogen). cDNA was diluted 1:10 prior to RT-qPCR performance.

For gene-expression analysis using RT-qPCR, cDNA was synthesized from 200 ng of total RNA diluted in 14 µL of nuclease-free water and

prepared using the SuperScript VILO cDNA Synthesis kit (Invitrogen), containing 4 μL of 5× VILO Reaction Mix and 2 μL of 10× SuperScript Enzyme Mix in a total volume of 20 μL. Cycling settings were 25°C for 10 min, 42°C for 60 min, and 85°C for 5 min cDNA samples were frozen at −20°C or immediately used for RT-qPCR performance. cDNA was diluted 1:10 prior to RT-qPCR performance. RT-qPCR analyses measuring SaCas9-KKH, gRNA expression, and downstream mmu-miR-21a mRNA targets were performed with specifically designed primers (Table S6) using SYBR green (Applied Biosystems). Gene expression was determined using the SYBR green protocol qPCR mix containing 1 ng/μL cDNA per reaction in a total volume of 20 μL per reaction, as prepared following the manufacturing protocol of Power SYBR Green PCR Master Mix (Applied Biosystems). Primers were diluted in nuclease-free water into a final concentration of 4 μM and forward and reverse primers were mixed in a 1:1 ratio. The cycling conditions using the fast protocol were 50°C for 2 min, 92°C for 10 min, and 40 cycles of 95°C for 1 s and 60°C for 20 s.

Gene expression was normalized to the appropriate housekeeping gene (U6 for microRNA, *β-Actin* for mRNA expression). Relative miRNA expression was then calculated by the 2(-Delta Delta C(T)) to show relative changes in gene expression. A fold difference for each gene was calculated by dividing its measurement in one group by its measurement in the other group to show the expression ratio as compared to the control. All RT-qPCR reactions were performed in triplicate and were carried out by the QuantStudio 3 PCR system (Applied Biosystems). The primer sequences used in this study are noted (Table S6).

Cell-proliferation assay

Cell proliferation was assessed *in vitro* by the water-soluble tetrazolium 1 (WST-1) reduction assay to determine cell viability (cell counting kit-8; Dojindo, Rockville, MD) of FACS-sorted GFP^{POS} cells. Cells were seeded at a low density (2×10^3 cells/well) in a 96-well plate. After 24 h, the medium was removed, and 10% WST-1 solution was added to the cells. The cells were incubated at 37°C for 1 h, and absorbance levels at wavelength 450 nm were measured using a microplate reader (SynergyH1; BioTek, Winooski, VT). Thereafter, the medium was changed, and cells were measured repeatedly every 24 h up until 90% confluency on day 7.

Immunohistochemistry staining

For imaging, cells were seeded on poly-D-lysine (PDL)-coated glass coverslips and incubated for 24 h prior to transfection. Transfection efficiency was analyzed 24 h post transfection. After transfection, cells were rinsed in PBS for 5 min and fixed using 4% paraformaldehyde (4% PFA) for 30 min. After fixation, cells were rinsed twice in PBS for 5 min each. Blocking was achieved by using 5% BSA and 0.1% Tween 20 in PBS (PBS-T) for 1 h. Cells were then incubated with the primary antibody GFP polyclonal antibody anti-rabbit (A11122; 1:400; Invitrogen), Ki-67 monoclonal antibody produced in rat (14569882; 1:400; Invitrogen) and primary antibody Anti-FLAG anti-rabbit (F7425; 1:400; Millipore) diluted in blocking buffer at 4°C overnight. Cells were rinsed three times in PBS-T for 5 min each. The secondary antibody Alexa Fluor 647 (catalog: A21245; goat anti-rabbit; Invitrogen; 1:400) (Invitrogen) was diluted in PBS-T and incubated for 1 h in the dark at RT. Coverslips were transferred to microscope slides (Fisher Scientific) on a droplet of mounting medium containing DAPI (Vectashield; Vector Laboratories). Fluorescence microscopy images were acquired on the Nikon W1-SoRa confocal microscope and processed using ImageJ2 (FIJI) version 2.9.0 software.

Brain slices on microscope slides (Fisher Scientific) were fixed with 4% PFA for 10 min at RT. After fixation, the slices were rinsed with PBS for 5 min and incubated for 10 min with PBS-T (1× PBS with 0.5% Triton X-100 [USB]), followed by 1-h blocking by using PBS-T including 5% goat serum (Gibco) for 1 h at RT. Brain slices were then incubated with the following primary antibodies: GFP (1:400, Invitrogen), GFAP (1:400, Invitrogen #13-0300), IBA1 (1:400, FujiFilm Wako #019-19741), OLIG2 (1:400 Abcam #ab109186), and NeuN (1:400 Abcam #ab177487) at 4°C overnight. Next day, tissue was rinsed three times in PBS-T for 5 min each. The appropriate secondary antibody (goat anti-rabbit, goat anti-rat, goat anti-mouse; Invitrogen) was diluted 1:400 in PBS-T and incubated for 1 h in the dark at RT. Slides were then mounted with mounting medium containing DAPI (Vectashield; Vector Labs). Images were acquired with the Nikon W1-SoRa confocal microscope and processed using ImageJ2 (FIJI) version 2.9.0 software.

TUNEL *in situ* apoptosis staining

One-step TUNEL In Situ Apoptosis Kit (green, fluorescein isothiocyanate [FITC]) catalyzes broken DNA by terminal deoxynucleotidyl transferase (TdT) with fluorescein-labeled deoxyuridine triphosphate (dUTP), which can be detected with fluorescence microscope (Elabscience). The TUNEL kit was used to detect cell apoptosis in brain sections of mice that were i.c. injected with either PBS or AAV8-control AAV8-CRISPR. AAV8 vectors (2×10^{10} gc/mL per mouse) in a total volume of 2 μL were then i.c. injected into the left striatum of C57BL/6J mice using a Hamilton syringe (Sigma-Aldrich, St. Louis, MO) as described above (intracranial tumor or vector implantation). 14 days post injection, mice were sacrificed and brains were harvested and frozen in O.C.T. tissue cryostat freezing medium (Fisher Scientific) and sectioned with the cryostat. Sections were incubated with 4% PFA for 30 min and washed with PBS 2 × 5 min. Brain sections were incubated with proteinase K at 37°C for 20 min and washed with PBS 3 × 5 min. The positive control was incubated with DNase I and negative control with buffer only for 30 min at 37°C and prepared according to the manufactures protocol. Slides were washed 3× with PBS for 5 min. The broken DNA can be catalyzed by TdT for 30 min at 37°C and then washed 3× with PBS for 5 min, stained for DAPI, and washed 4× with PBS for 5 min. Brain sections were analyzed using the FITC channel of the Nikon W1-SoRa confocal microscope and processed using ImageJ2 (FIJI) version 2.9.0 software.

AAV vector transduction *in vitro*

For *in vitro* testing of the AAV vectors, CT-2A and GL261 cells were plated in 24-well plates, and, 1 day after seeding, 2 μL of

AAV8 (2×10^{10} gc/μL per mouse) either AAV8-CRISPR or AAV8-control was added to each well and incubated for 48 h. Medium was refreshed and cells were cultured for 6 days. To determine mmu-miR-21a levels, cells were pelleted and RNA was extracted. Samples were processed according to the RT-qPCR protocol.

For immunohistochemistry (IHC) staining, 1×10^5 CT-2A or GL261 cells/well were plated on FBS-coated coverslips in a 24-well plate (Corning) and 2 μL of packaged AAV8-GFP^{POS} (6×10^{10} gc/μL) was added. After 7 days, cells were fixated and processed according to the IHC protocol.

Western blots

Total protein was extracted from cultured cells using RIPA lysis buffer (Thermo Fisher Scientific) supplemented with a protease inhibitor cocktail (Sigma-Aldrich). To remove non-soluble cell debris, samples were sonicated using a probe sonicator (Sonic Dismembrator Model 100, Thermo Fisher Scientific) at a setting of 3.0 for 5 s and centrifuged at 15,000 × *g* for 10 min at 4°C. Protein concentration was determined using the Pierce BCA Protein Assay Kit (Thermo Fisher Scientific). Absorbance was measured at 562 nm using the SynergyHI microplate reader (BioTek). Equal amounts of protein (20 μg) mixed with Laemmli SDS-Sample buffer (Boston BioProducts, Milford, MA) were loaded and resolved by electrophoresis on NuPage 4%–12% Bis-Tris polyacrylamide gels (Thermo Fisher Scientific) in NuPage MES SDS Running Buffer (Thermo Fisher Scientific). After transfer onto nitrocellulose membranes using the iBlot 2 (Thermo Fisher Scientific), samples were subsequently incubated for 1 h at RT on an orbital shaker in 5% nonfat dry milk (LabScientific, Danvers, MA) in Tris-buffered saline (pH = 7.4) with 0.05% Tween 20 (TBS-T). After blocking, the membrane was probed with primary antibody anti-mouse FLAG-tag 1:1000 (Merck, F3165) or β-actin anti-goat (Santa Cruz Biotechnology, Dallas, TX, I-19) overnight at 4°C. After washing three times with TBS-T for 10 min, membranes were incubated for 1 h at RT with secondary antibodies ECL donkey-anti-goat immunoglobulin G (IgG) (Sigma-Aldrich) and ECL sheep-anti-mouse IgG (Thermo Fisher Scientific) (1:5,000) corresponding to the primary antibodies. Membranes were developed with ECL or Femto staining (Thermo Fisher Scientific) and imaged on an Azure Biosystems C300 gel imager.

Statistical analysis

All data were tested for normality using the Shapiro-Wilkinson test and pairwise comparisons were tested for equal variances using the Levene's test. To analyze the pairwise comparisons of RT-qPCR data, unpaired t test and multiple-comparison one-way ANOVA were performed. Proliferation assay and bioluminescence data were analyzed using a two-way ANOVA. Kaplan-Meier survival experiments were analyzed using the log rank (Mantel-Cox) test. GraphPad Prism 10.2.0 was used for all statistical analyses and a significance level of $\alpha \leq 0.05$ was used for analysis and for graphical display of the results.

DATA AND CODE AVAILABILITY

The data supporting the findings described in this study are available within the article and in the supplementary materials. The NGS raw data (FASTQ files and processed data) discussed in this publication have been deposited in NCBI's Gene Expression Omnibus (GEO)[103] and are accessible through GEO Series accession number GSE277282 (https://www.ncbi.nlm.nih.gov/geo/query/acc.cgi?&acc=GSE277282). Raw processed RT-qPCR data are available on reasonable request.

ACKNOWLEDGMENTS

We thank all members of the Breakefield laboratory for their suggested ideas during laboratory meetings. We thank all laboratories within the Molecular Neurogenetics Unit at MGH for their input. Special thanks to members of the Maguire laboratory for sharing their expertise on AAV vector cloning and delivery. We thank Mrs. Suzanne McDavitt for her skilled editorial assistance. X.O.B. acknowledges grant support from the National Institutes of Health (NIH) NCI CA179563, CA069246, and CA232103 and NINDS NS122163 grants for supporting this work. U19 CA179563 was supported by the NIH Common Fund, through the Office of Strategic Coordination/Office of the NIH Director. L.N. is supported by Prins Bernhard Cultuur fonds. K.B. acknowledges support from NIH K22 (K22CA282019). D.R.R. is supported by Friedreich's Ataxia Research Alliance (FARA) and FARA Australia. B.P.K. was supported by the Kayden-Lambert MGH Research Scholar Award and NIH grants P01-HL142494 and DP2-CA281401. C.A.M. discloses support for the research described in this study from the NIH, DC017117. E.R.A. and M.L.D.B are supported by the Hersenstichting (DNFC).

AUTHOR CONTRIBUTIONS

X.O.B., E.R.A., and L.N. conceived the study and designed experiments. X.O.B., M.L.D.B., and E.R.A. supervised the project. B.P.K. designed the guides, provided the pUC19 and SaCas9-KKH plasmids, and assisted with genome-editing approaches. C.A.M. provided the AAV8-GFP and shared his expertise on AAV cloning and experiments. K.B. and E.R.A. designed cloning strategies. D.R.-R. cloned the all-in-one plasmids and assisted with analysis of genome-editing experiments. L.N., A.B.V., I.W.J., and A.J.E.M.d.R. performed and analyzed the experiments. L.N. prepared the figures. L.N. and E.R.A. wrote the manuscript. All authors edited or commented on the manuscript.

DECLARATION OF INTERESTS

B.P.K. is an inventor of patents or patent applications filed by Mass General Brigham (MGB) that describe genome engineering technologies. B.P.K. is a consultant for EcoR1 capital and Novartis Venture Fund and is on the scientific advisory boards of Acrigen Biosciences, Life Edit Therapeutics, and Prime Medicine. B.P.K. has a financial interest in Prime Medicine, Inc., a company developing therapeutic CRISPR-Cas technologies for gene editing. B.P.K.'s interests were reviewed and are managed by MGH and MGB in accordance with their conflict-of-interest policies.

C.A.M. has financial interests in Chameleon Biosciences, Skylark Bio, and Sphere Gene Therapeutics, companies developing AAV vector technologies for gene therapy applications. C.A.M. performs paid consulting work for all three companies. C.A.M.'s interests were reviewed and are managed by MGH and MGB in accordance with their conflict-of-interest policies.

SUPPLEMENTAL INFORMATION

Supplemental information can be found online at https://doi.org/10.1016/j.ymthe.2024.11.023.

REFERENCES

1. Miller, K.D., Ostrom, Q.T., Kruchko, C., Patil, N., Tihan, T., Cioffi, G., Fuchs, H.E., Waite, K.A., Jemal, A., Siegel, R.L., and Barnholtz-Sloan, J.S. (2021). Brain and other central nervous system tumor statistics, 2021. CA Cancer J. Clin. *71*, 381–406. https://doi.org/10.3322/caac.21693.

2. van Solinge, T.S., Nieland, L., Chiocca, E.A., and Broekman, M.L.D. (2022). Advances in local therapy for glioblastoma - taking the fight to the tumour. Nat. Rev. Neurol. *18*, 221–236. https://doi.org/10.1038/s41582-022-00621-0.

3. Stupp, R., Mason, W.P., van den Bent, M.J., Weller, M., Fisher, B., Taphoorn, M.J.B., Belanger, K., Brandes, A.A., Marosi, C., Bogdahn, U., et al. (2005). Radiotherapy plus

concomitant and adjuvant temozolomide for glioblastoma. N. Engl. J. Med. *352*, 987–996. https://doi.org/10.1056/NEJMoa043330.

4. Ostrom, Q.T., Cote, D.J., Ascha, M., Kruchko, C., and Barnholtz-Sloan, J.S. (2018). Adult Glioma Incidence and Survival by Race or Ethnicity in the United States From 2000 to 2014. JAMA Oncol. *4*, 1254–1262. https://doi.org/10.1001/jamaoncol.2018.1789.

5. Ciafre, S.A., Galardi, S., Mangiola, A., Ferracin, M., Liu, C.G., Sabatino, G., Negrini, M., Maira, G., Croce, C.M., and Farace, M.G. (2005). Extensive modulation of a set of microRNAs in primary glioblastoma. Biochem. Biophys. Res. Commun. *334*, 1351–1358. https://doi.org/10.1016/j.bbrc.2005.07.030.

6. Hashemi, M., Mirdamadi, M.S.A., Talebi, Y., Khaniabad, N., Banaei, G., Daneii, P., Gholami, S., Ghorbani, A., Tavakolpournegari, A., Farsani, Z.M., et al. (2023). Preclinical and clinical importance of miR-21 in human cancers: Tumorigenesis, therapy response, delivery approaches and targeting agents. Pharmacol. Res. *187*, 106568. https://doi.org/10.1016/j.phrs.2022.106568.

7. Chan, J.A., Krichevsky, A.M., and Kosik, K.S. (2005). MicroRNA-21 is an antiapoptotic factor in human glioblastoma cells. Cancer Res. *65*, 6029–6033. https://doi.org/10.1158/0008-5472.CAN-05-0137.

8. Lakomy, R., Sana, J., Hankeova, S., Fadrus, P., Kren, L., Lzicarova, E., Svoboda, M., Dolezelova, H., Smrcka, M., Vyzula, R., et al. (2011). MiR-195, miR-196b, miR-181c, miR-21 expression levels and O-6-methylguanine-DNA methyltransferase methylation status are associated with clinical outcome in glioblastoma patients. Cancer Sci. *102*, 2186–2190. https://doi.org/10.1111/j.1349-7006.2011.02092.x.

9. Baraniskin, A., Kuhnhenn, J., Schlegel, U., Maghnouj, A., Zöllner, H., Schmiegel, W., Hahn, S., and Schroers, R. (2012). Identification of microRNAs in the cerebrospinal fluid as biomarker for the diagnosis of glioma. Neuro. Oncol. *14*, 29–33. https://doi.org/10.1093/neuonc/nor169.

10. Jiang, G., Mu, J., Liu, X., Peng, X., Zhong, F., Yuan, W., Deng, F., Peng, X., Peng, S., and Zeng, X. (2020). Prognostic value of miR-21 in gliomas: comprehensive study based on meta-analysis and TCGA dataset validation. Sci. Rep. *10*, 4220. https://doi.org/10.1038/s41598-020-61155-3.

11. Aloizou, A.M., Pateraki, G., Siokas, V., Mentis, A.F.A., Liampas, I., Lazopoulos, G., Kovatsi, L., Mitsias, P.D., Bogdanos, D.P., Paterakis, K., and Dardiotis, E. (2020). The role of MiRNA-21 in gliomas: Hope for a novel therapeutic intervention? Toxicol. Rep. *7*, 1514–1530. https://doi.org/10.1016/j.toxrep.2020.11.001.

12. Doudna, J.A. (2020). The promise and challenge of therapeutic genome editing. Nature *578*, 229–236. https://doi.org/10.1038/s41586-020-1978-5.

13. Wang, M., Glass, Z.A., and Xu, Q. (2017). Non-viral delivery of genome-editing nucleases for gene therapy. Gene Ther. *24*, 144–150. https://doi.org/10.1038/gt.2016.72.

14. Glasner, A., Rose, S.A., Sharma, R., Gudjonson, H., Chu, T., Green, J.A., Rampersaud, S., Valdez, I.K., Andretta, E.S., Dhillon, B.S., et al. (2023). Conserved transcriptional connectivity of regulatory T cells in the tumor microenvironment informs new combination cancer therapy strategies. Nat. Immunol. *24*, 1020–1035. https://doi.org/10.1038/s41590-023-01504-2.

15. Ran, F.A., Cong, L., Yan, W.X., Scott, D.A., Gootenberg, J.S., Kriz, A.J., Zetsche, B., Shalem, O., Wu, X., Makarova, K.S., et al. (2015). In vivo genome editing using Staphylococcus aureus Cas9. Nature *520*, 186–191. https://doi.org/10.1038/nature14299.

16. Friedland, A.E., Baral, R., Singhal, P., Loveluck, K., Shen, S., Sanchez, M., Marco, E., Gotta, G.M., Maeder, M.L., Kennedy, E.M., et al. (2015). Characterization of Staphylococcus aureus Cas9: a smaller Cas9 for all-in-one adeno-associated virus delivery and paired nickase applications. Genome Biol. *16*, 257. https://doi.org/10.1186/s13059-015-0817-8.

17. Kleinstiver, B.P., Prew, M.S., Tsai, S.Q., Nguyen, N.T., Topkar, V.V., Zheng, Z., and Joung, J.K. (2015). Broadening the targeting range of Staphylococcus aureus CRISPR-Cas9 by modifying PAM recognition. Nat. Biotechnol. *33*, 1293–1298. https://doi.org/10.1038/nbt.3404.

18. Wang, J.H., Gessler, D.J., Zhan, W., Gallagher, T.L., and Gao, G. (2024). Adeno-associated virus as a delivery vector for gene therapy of human diseases. Signal Transduct. Target. Ther. *9*, 78. https://doi.org/10.1038/s41392-024-01780-w.

19. Xu, X., Chen, W., Zhu, W., Chen, J., Ma, B., Ding, J., Wang, Z., Li, Y., Wang, Y., and Zhang, X. (2021). Adeno-associated virus (AAV)-based gene therapy for glioblastoma. Cancer Cell Int. *21*, 76. https://doi.org/10.1186/s12935-021-01776-4.

20. Varela, M.L., Comba, A., Faisal, S.M., Argento, A., Franson, A., Barissi, M.N., Sachdev, S., Castro, M.G., and Lowenstein, P.R. (2023). Gene Therapy for High Grade Glioma: The Clinical Experience. Expert Opin. Biol. Ther. *23*, 145–161. https://doi.org/10.1080/14712598.2022.2157718.

21. Maguire, C.A., Gianni, D., Meijer, D.H., Shaket, L.A., Wakimoto, H., Rabkin, S.D., Gao, G., and Sena-Esteves, M. (2010). Directed evolution of adeno-associated virus for glioma cell transduction. J. Neurooncol. *96*, 337–347. https://doi.org/10.1007/s11060-009-9972-7.

22. Meijer, D.H., Maguire, C.A., LeRoy, S.G., and Sena-Esteves, M. (2009). Controlling brain tumor growth by intraventricular administration of an AAV vector encoding IFN-beta. Cancer Gene Ther. *16*, 664–671. https://doi.org/10.1038/cgt.2009.8.

23. GuhaSarkar, D., Su, Q., Gao, G., and Sena-Esteves, M. (2016). Systemic AAV9-IFNbeta gene delivery treats highly invasive glioblastoma. Neuro. Oncol. *18*, 1508–1518. https://doi.org/10.1093/neuonc/now097.

24. Crommentuijn, M.H., Kantar, R., Noske, D.P., Vandertop, W.P., Badr, C.E., Würdinger, T., Maguire, C.A., and Tannous, B.A. (2016). Systemically administered AAV9-sTRAIL combats invasive glioblastoma in a patient-derived orthotopic xenograft model. Mol. Ther. Oncolytics *3*, 16017. https://doi.org/10.1038/mto.2016.17.

25. Grieger, J.C., and Samulski, R.J. (2012). Adeno-associated virus vectorology, manufacturing, and clinical applications. Methods Enzymol. *507*, 229–254. https://doi.org/10.1016/B978-0-12-386509-0.00012-0.

26. Raguram, A., Banskota, S., and Liu, D.R. (2022). Therapeutic in vivo delivery of gene editing agents. Cell *185*, 2806–2827. https://doi.org/10.1016/j.cell.2022.03.045.

27. Enger, P.Ø., Thorsen, F., Lønning, P.E., Bjerkvig, R., and Hoover, F. (2002). Adeno-associated viral vectors penetrate human solid tumor tissue in vivo more effectively than adenoviral vectors. Hum. Gene Ther. *13*, 1115–1125. https://doi.org/10.1089/104303402753812511.

28. Li, Q., Su, J., Liu, Y., Jin, X., Zhong, X., Mo, L., Wang, Q., Deng, H., and Yang, Y. (2021). In vivo PCSK9 gene editing using an all-in-one self-cleavage AAV-CRISPR system. Mol. Ther. Methods Clin. Dev. *20*, 652–659. https://doi.org/10.1016/j.omtm.2021.02.005.

29. Zhang, Y., Nishiyama, T., Li, H., Huang, J., Atmanli, A., Sanchez-Ortiz, E., Wang, Z., Mireault, A.A., Mammen, P.P.A., Bassel-Duby, R., and Olson, E.N. (2021). A consolidated AAV system for single-cut CRISPR correction of a common Duchenne muscular dystrophy mutation. Mol. Ther. Methods Clin. Dev. *22*, 122–132. https://doi.org/10.1016/j.omtm.2021.05.014.

30. Ekman, F.K., Ojala, D.S., Adil, M.M., Lopez, P.A., Schaffer, D.V., and Gaj, T. (2019). CRISPR-Cas9-Mediated Genome Editing Increases Lifespan and Improves Motor Deficits in a Huntington's Disease Mouse Model. Mol. Ther. Nucleic Acids *17*, 829–839. https://doi.org/10.1016/j.omtn.2019.07.009.

31. Davis, J.R., Wang, X., Witte, I.P., Huang, T.P., Levy, J.M., Raguram, A., Banskota, S., Seidah, N.G., Musunuru, K., and Liu, D.R. (2022). Efficient in vivo base editing via single adeno-associated viruses with size-optimized genomes encoding compact adenine base editors. Nat. Biomed. Eng. *6*, 1272–1283. https://doi.org/10.1038/s41551-022-00911-4.

32. Zhang, H., Bamidele, N., Liu, P., Ojelabi, O., Gao, X.D., Rodriguez, T., Cheng, H., Kelly, K., Watts, J.K., Xie, J., et al. (2022). Adenine Base Editing In Vivo with a Single Adeno-Associated Virus Vector. GEN. Biotechnol. *1*, 285–299. https://doi.org/10.1089/genbio.2022.0015.

33. Ibraheim, R., Tai, P.W.L., Mir, A., Javeed, N., Wang, J., Rodríguez, T.C., Namkung, S., Nelson, S., Khokhar, E.S., Mintzer, E., et al. (2021). Self-inactivating, all-in-one AAV vectors for precision Cas9 genome editing via homology-directed repair in vivo. Nat. Commun. *12*, 6267. https://doi.org/10.1038/s41467-021-26518-y.

34. Tan, Y., Chu, A.H.Y., Bao, S., Hoang, D.A., Kebede, F.T., Xiong, W., Ji, M., Shi, J., and Zheng, Z. (2019). Rationally engineered Staphylococcus aureus Cas9 nucleases with high genome-wide specificity. Proc. Natl. Acad. Sci. USA *116*, 20969–20976. https://doi.org/10.1073/pnas.1906843116.

35. Nieland, L., van Solinge, T.S., Cheah, P.S., Morsett, L.M., El Khoury, J., Rissman, J.I., Kleinstiver, B.P., Broekman, M.L.D., Breakefield, X.O., and Abels, E.R. (2022).

CRISPR-Cas knockout of miR21 reduces glioma growth. Mol. Ther. Oncolytics *25*, 121–136. https://doi.org/10.1016/j.omto.2022.04.001.

36. Cai, X., Hagedorn, C.H., and Cullen, B.R. (2004). Human microRNAs are processed from capped, polyadenylated transcripts that can also function as mRNAs. RNA *10*, 1957–1966. https://doi.org/10.1261/rna.7135204.

37. Ribas, J., Ni, X., Castanares, M., Liu, M.M., Esopi, D., Yegnasubramanian, S., Rodriguez, R., Mendell, J.T., and Lupold, S.E. (2012). A novel source for miR-21 expression through the alternative polyadenylation of VMP1 gene transcripts. Nucleic Acids Res. *40*, 6821–6833. https://doi.org/10.1093/nar/gks308.

38. Hacker, U.T., Bentler, M., Kaniowska, D., Morgan, M., and Büning, H. (2020). Towards Clinical Implementation of Adeno-Associated Virus (AAV) Vectors for Cancer Gene Therapy: Current Status and Future Perspectives. Cancers (Basel) *12*, 1889. https://doi.org/10.3390/cancers12071889.

39. Broekman, M.L.D., Comer, L.A., Hyman, B.T., and Sena-Esteves, M. (2006). Adeno-associated virus vectors serotyped with AAV8 capsid are more efficient than AAV-1 or -2 serotypes for widespread gene delivery to the neonatal mouse brain. Neuroscience *138*, 501–510. https://doi.org/10.1016/j.neuroscience.2005.11.057.

40. Harding, T.C., Dickinson, P.J., Roberts, B.N., Yendluri, S., Gonzalez-Edick, M., Lecouteur, R.A., and Jooss, K.U. (2006). Enhanced gene transfer efficiency in the murine striatum and an orthotopic glioblastoma tumor model, using AAV-7- and AAV-8-pseudotyped vectors. Hum. Gene Ther. *17*, 807–820. https://doi.org/10.1089/hum.2006.17.807.

41. Dou, Y., Lin, Y., Wang, T.Y., Wang, X.Y., Jia, Y.L., and Zhao, C.P. (2021). The CAG promoter maintains high-level transgene expression in HEK293 cells. FEBS Open Bio *11*, 95–104. https://doi.org/10.1002/2211-5463.13029.

42. Pignataro, D., Sucunza, D., Vanrell, L., Lopez-Franco, E., Dopeso-Reyes, I.G., Vales, A., Hommel, M., Rico, A.J., Lanciego, J.L., and Gonzalez-Aseguinolaza, G. (2017). Adeno-Associated Viral Vectors Serotype 8 for Cell-Specific Delivery of Therapeutic Genes in the Central Nervous System. Front. Neuroanat. *11*, 2. https://doi.org/10.3389/fnana.2017.00002.

43. McLeod, F., McDermott, E., Mak, S., Walsh, D., Turnbull, M., LeBeau, F.E.N., Jackson, A., Trevelyan, A.J., and Clowry, G.J. (2024). AAV8 vector induced gliosis following neuronal transgene expression. Front. Neurosci. *18*, 1287228. https://doi.org/10.3389/fnins.2024.1287228.

44. Cearley, C.N., and Wolfe, J.H. (2006). Transduction characteristics of adeno-associated virus vectors expressing cap serotypes 7, 8, 9, and Rh10 in the mouse brain. Mol. Ther. *13*, 528–537. https://doi.org/10.1016/j.ymthe.2005.11.015.

45. Zincarelli, C., Soltys, S., Rengo, G., and Rabinowitz, J.E. (2008). Analysis of AAV serotypes 1-9 mediated gene expression and tropism in mice after systemic injection. Mol. Ther. *16*, 1073–1080. https://doi.org/10.1038/mt.2008.76.

46. Asokan, A., and Shen, S. (2023). Redirecting AAV vectors to extrahepatic tissues. Mol. Ther. *31*, 3371–3375. https://doi.org/10.1016/j.ymthe.2023.10.005.

47. Jagadisan, B., and Dhawan, A. (2023). Multidisciplinary approach for gene therapy-related hepatotoxicity. J. Thromb. Haemost. *21*, 1998–1999. https://doi.org/10.1016/j.jtha.2023.03.043.

48. Lang, J.F., Toulmin, S.A., Brida, K.L., Eisenlohr, L.C., and Davidson, B.L. (2019). Standard screening methods underreport AAV-mediated transduction and gene editing. Nat. Commun. *10*, 3415. https://doi.org/10.1038/s41467-019-11321-7.

49. Meng, F., Henson, R., Wehbe-Janek, H., Ghoshal, K., Jacob, S.T., and Patel, T. (2007). MicroRNA-21 regulates expression of the PTEN tumor suppressor gene in human hepatocellular cancer. Gastroenterology *133*, 647–658. https://doi.org/10.1053/j.gastro.2007.05.022.

50. Chen, Y., Liu, W., Chao, T., Zhang, Y., Yan, X., Gong, Y., Qiang, B., Yuan, J., Sun, M., and Peng, X. (2008). MicroRNA-21 down-regulates the expression of tumor suppressor PDCD4 in human glioblastoma cell T98G. Cancer Lett. *272*, 197–205. https://doi.org/10.1016/j.canlet.2008.06.034.

51. Li, F., Liu, J., Park, E.S., Jo, M., and Curry, T.E., Jr. (2009). The B cell translocation gene (BTG) family in the rat ovary: hormonal induction, regulation, and impact on cell cycle kinetics. Endocrinology *150*, 3894–3902. https://doi.org/10.1210/en.2008-1650.

52. Li, J., Zhao, L., He, X., Yang, T., and Yang, K. (2014). MiR-21 inhibits c-Ski signaling to promote the proliferation of rat vascular smooth muscle cells. Cell. Signal. *26*, 724–729. https://doi.org/10.1016/j.cellsig.2013.12.013.

53. Amoasii, L., Hildyard, J.C.W., Li, H., Sanchez-Ortiz, E., Mireault, A., Caballero, D., Harron, R., Stathopoulou, T.R., Massey, C., Shelton, J.M., et al. (2018). Gene editing restores dystrophin expression in a canine model of Duchenne muscular dystrophy. Science *362*, 86–91. https://doi.org/10.1126/science.aau1549.

54. Bengtsson, N.E., Hall, J.K., Odom, G.L., Phelps, M.P., Andrus, C.R., Hawkins, R.D., Hauschka, S.D., Chamberlain, J.R., and Chamberlain, J.S. (2017). Muscle-specific CRISPR/Cas9 dystrophin gene editing ameliorates pathophysiology in a mouse model for Duchenne muscular dystrophy. Nat. Commun. *8*, 14454. https://doi.org/10.1038/ncomms14454.

55. Gyorgy, B., Loov, C., Zaborowski, M.P., Takeda, S., Kleinstiver, B.P., Commins, C., Kastanenka, K., Mu, D., Volak, A., Giedraitis, V., et al. (2018). CRISPR/Cas9 Mediated Disruption of the Swedish APP Allele as a Therapeutic Approach for Early-Onset Alzheimer's Disease. Mol. Ther. Nucleic Acids *11*, 429–440. https://doi.org/10.1016/j.omtn.2018.03.007.

56. Ibraheim, R., Song, C.Q., Mir, A., Amrani, N., Xue, W., and Sontheimer, E.J. (2018). All-in-one adeno-associated virus delivery and genome editing by Neisseria meningitidis Cas9 in vivo. Genome Biol. *19*, 137. https://doi.org/10.1186/s13059-018-1515-0.

57. McCullough, K.T., Boye, S.L., Fajardo, D., Calabro, K., Peterson, J.J., Strang, C.E., Chakraborty, D., Gloskowski, S., Haskett, S., Samuelsson, S., et al. (2019). Somatic Gene Editing of GUCY2D by AAV-CRISPR/Cas9 Alters Retinal Structure and Function in Mouse and Macaque. Hum. Gene Ther. *30*, 571–589. https://doi.org/10.1089/hum.2018.193.

58. Ruan, G.X., Barry, E., Yu, D., Lukason, M., Cheng, S.H., and Scaria, A. (2017). CRISPR/Cas9-Mediated Genome Editing as a Therapeutic Approach for Leber Congenital Amaurosis 10. Mol. Ther. *25*, 331–341. https://doi.org/10.1016/j.ymthe.2016.12.006.

59. Maeder, M.L., Stefanidakis, M., Wilson, C.J., Baral, R., Barrera, L.A., Bounoutas, G.S., Bumcrot, D., Chao, H., Ciulla, D.M., DaSilva, J.A., et al. (2019). Development of a gene-editing approach to restore vision loss in Leber congenital amaurosis type 10. Nat. Med. *25*, 229–233. https://doi.org/10.1038/s41591-018-0327-9.

60. Jiang, Y., Xie, M., Chen, W., Talbot, R., Maddox, J.F., Faraut, T., Wu, C., Muzny, D.M., Li, Y., Zhang, W., et al. (2014). The sheep genome illuminates biology of the rumen and lipid metabolism. Science *344*, 1168–1173. https://doi.org/10.1126/science.1252806.

61. Moreno, A.M., Fu, X., Zhu, J., Katrekar, D., Shih, Y.R.V., Marlett, J., Cabotaje, J., Tat, J., Naughton, J., Lisowski, L., et al. (2018). In Situ Gene Therapy via AAV-CRISPR-Cas9-Mediated Targeted Gene Regulation. Mol. Ther. *26*, 1818–1827. https://doi.org/10.1016/j.ymthe.2018.04.017.

62. Harding, T.C., Lalani, A.S., Roberts, B.N., Yendluri, S., Luan, B., Koprivnikar, K.E., Gonzalez-Edick, M., Huan-Tu, G., Musterer, R., VanRoey, M.J., et al. (2006). AAV serotype 8-mediated gene delivery of a soluble VEGF receptor to the CNS for the treatment of glioblastoma. Mol. Ther. *13*, 956–966. https://doi.org/10.1016/j.ymthe.2006.02.004.

63. Maguire, C.A., Meijer, D.H., LeRoy, S.G., Tierney, L.A., Broekman, M.L.D., Costa, F.F., Breakefield, X.O., Stemmer-Rachamimov, A., and Sena-Esteves, M. (2008). Preventing growth of brain tumors by creating a zone of resistance. Mol. Ther. *16*, 1695–1702. https://doi.org/10.1038/mt.2008.168.

64. Venkataramani, V., Yang, Y., Schubert, M.C., Reyhan, E., Tetzlaff, S.K., Wißmann, N., Botz, M., Soyka, S.J., Beretta, C.A., Pramatarov, R.L., et al. (2022). Glioblastoma hijacks neuronal mechanisms for brain invasion. Cell *185*, 2899–2917.e31. https://doi.org/10.1016/j.cell.2022.06.054.

65. Davidsson, M., Wang, G., Aldrin-Kirk, P., Cardoso, T., Nolbrant, S., Hartnor, M., Mudannayake, J., Parmar, M., and Björklund, T. (2019). A systematic capsid evolution approach performed in vivo for the design of AAV vectors with tailored properties and tropism. Proc. Natl. Acad. Sci. USA *116*, 27053–27062. https://doi.org/10.1073/pnas.1910061116.

66. Hanlon, K.S., Kleinstiver, B.P., Garcia, S.P., Zaborowski, M.P., Volak, A., Spirig, S.E., Muller, A., Sousa, A.A., Tsai, S.Q., Bengtsson, N.E., et al. (2019). High levels of AAV vector integration into CRISPR-induced DNA breaks. Nat. Commun. *10*, 4439. https://doi.org/10.1038/s41467-019-12449-2.

67. Seo, Y.E., Suh, H.W., Bahal, R., Josowitz, A., Zhang, J., Song, E., Cui, J., Noorbakhsh, S., Jackson, C., Bu, T., et al. (2019). Nanoparticle-mediated intratumoral inhibition

of miR-21 for improved survival in glioblastoma. Biomaterials *201*, 87–98. https://doi.org/10.1016/j.biomaterials.2019.02.016.

68. Belter, A., Rolle, K., Piwecka, M., Fedoruk-Wyszomirska, A., Naskręt-Barciszewska, M.Z., and Barciszewski, J. (2016). Inhibition of miR-21 in glioma cells using catalytic nucleic acids. Sci. Rep. *6*, 24516. https://doi.org/10.1038/srep24516.

69. Hanna, J., Hossain, G.S., and Kocerha, J. (2019). The Potential for microRNA Therapeutics and Clinical Research. Front. Genet. *10*, 478. https://doi.org/10.3389/fgene.2019.00478.

70. Ho, P.T.B., Clark, I.M., and Le, L.T.T. (2022). MicroRNA-Based Diagnosis and Therapy. Int. J. Mol. Sci. *23*, 7167. https://doi.org/10.3390/ijms23137167.

71. Kim, T., and Croce, C.M. (2023). MicroRNA: trends in clinical trials of cancer diagnosis and therapy strategies. Exp. Mol. Med. *55*, 1314–1321. https://doi.org/10.1038/s12276-023-01050-9.

72. Krutzfeldt, J., Kuwajima, S., Braich, R., Rajeev, K.G., Pena, J., Tuschl, T., Manoharan, M., and Stoffel, M. (2007). Specificity, duplex degradation and subcellular localization of antagomirs. Nucleic Acids Res. *35*, 2885–2892. https://doi.org/10.1093/nar/gkm024.

73. Corsten, M.F., Miranda, R., Kasmieh, R., Krichevsky, A.M., Weissleder, R., and Shah, K. (2007). MicroRNA-21 knockdown disrupts glioma growth in vivo and displays synergistic cytotoxicity with neural precursor cell delivered S-TRAIL in human gliomas. Cancer Res. *67*, 8994–9000. https://doi.org/10.1158/0008-5472.CAN-07-1045.

74. Lee, T.J., Yoo, J.Y., Shu, D., Li, H., Zhang, J., Yu, J.G., Jaime-Ramirez, A.C., Acunzo, M., Romano, G., Cui, R., et al. (2017). RNA Nanoparticle-Based Targeted Therapy for Glioblastoma through Inhibition of Oncogenic miR-21. Mol. Ther. *25*, 1544–1555. https://doi.org/10.1016/j.ymthe.2016.11.016.

75. Wang, J.Y., and Doudna, J.A. (2023). CRISPR technology: A decade of genome editing is only the beginning. Science *379*, eadd8643. https://doi.org/10.1126/science.add8643.

76. Kang, X., Wang, Y., Liu, P., Huang, B., Zhou, B., Lu, S., Geng, W., and Tang, H. (2023). Progresses, Challenges, and Prospects of CRISPR/Cas9 Gene-Editing in Glioma Studies. Cancers (Basel) *15*, 396. https://doi.org/10.3390/cancers15020396.

77. Liu, Z., Feng, J., and Shen, Y. (2023). The Application of CRISPR-Cas System in Glioblastoma Research and Treatment. BIO Web of Conferences *60*, 01011. https://doi.org/10.1051/bioconf/20236001011.

78. Huang, K., Yang, C., Wang, Q.X., Li, Y.S., Fang, C., Tan, Y.L., Wei, J.W., Wang, Y.F., Li, X., Zhou, J.H., et al. (2017). The CRISPR/Cas9 system targeting EGFR exon 17 abrogates NF-kappaB activation via epigenetic modulation of UBXN1 in EGFRwt/vIII glioma cells. Cancer Lett. *388*, 269–280. https://doi.org/10.1016/j.canlet.2016.12.011.

79. Moure, C.J., Diplas, B.H., Chen, L.H., Yang, R., Pirozzi, C.J., Wang, Z., Spasojevic, I., Waitkus, M.S., He, Y., and Yan, H. (2019). CRISPR Editing of Mutant IDH1 R132H Induces a CpG Methylation-Low State in Patient-Derived Glioma Models of G-CIMP. Mol. Cancer Res. *17*, 2042–2050. https://doi.org/10.1158/1541-7786.MCR-19-0309.

80. Fierro, J., Jr., DiPasquale, J., Perez, J., Chin, B., Chokpapone, Y., Tran, A.M., Holden, A., Factoriza, C., Sivagnanakumar, N., Aguilar, R., et al. (2022). Dual-sgRNA CRISPR/Cas9 knockout of PD-L1 in human U87 glioblastoma tumor cells inhibits proliferation, invasion, and tumor-associated macrophage polarization. Sci. Rep. *12*, 2417. https://doi.org/10.1038/s41598-022-06430-1.

81. Morimoto, T., Nakazawa, T., Matsuda, R., Nishimura, F., Nakamura, M., Yamada, S., Nakagawa, I., Park, Y.S., Tsujimura, T., and Nakase, H. (2021). CRISPR-Cas9-Mediated TIM3 Knockout in Human Natural Killer Cells Enhances Growth Inhibitory Effects on Human Glioma Cells. Int. J. Mol. Sci. *22*, 3489. https://doi.org/10.3390/ijms22073489.

82. Kolliopoulos, C., Ali, M.M., Castillejo-Lopez, C., Heldin, C.H., and Heldin, P. (2022). CD44 Depletion in Glioblastoma Cells Suppresses Growth and Stemness and Induces Senescence. Cancers (Basel) *14*, 3747. https://doi.org/10.3390/cancers14153747.

83. Zou, Y., Sun, X., Yang, Q., Zheng, M., Shimoni, O., Ruan, W., Wang, Y., Zhang, D., Yin, J., Huang, X., et al. (2022). Blood-brain barrier-penetrating single CRISPR-Cas9 nanocapsules for effective and safe glioblastoma gene therapy. Sci. Adv. *8*, eabm8011. https://doi.org/10.1126/sciadv.abm8011.

84. Chen, K., Stahl, E.C., Kang, M.H., Xu, B., Allen, R., Trinidad, M., and Doudna, J.A. (2024). Engineering self-deliverable ribonucleoproteins for genome editing in the brain. Nat. Commun. *15*, 1727. https://doi.org/10.1038/s41467-024-45998-2.

85. Santiago-Ortiz, J.L., and Schaffer, D.V. (2016). Adeno-associated virus (AAV) vectors in cancer gene therapy. J. Control Release *240*, 287–301. https://doi.org/10.1016/j.jconrel.2016.01.001.

86. Bulcha, J.T., Wang, Y., Ma, H., Tai, P.W.L., and Gao, G. (2021). Viral vector platforms within the gene therapy landscape. Signal Transduct. Target. Ther. *6*, 53. https://doi.org/10.1038/s41392-021-00487-6.

87. Kuzmin, D.A., Shutova, M.V., Johnston, N.R., Smith, O.P., Fedorin, V.V., Kukushkin, Y.S., van der Loo, J.C.M., and Johnstone, E.C. (2021). The clinical landscape for AAV gene therapies. Nat. Rev. Drug Discov. *20*, 173–174. https://doi.org/10.1038/d41573-021-00017-7.

88. Leandro, K., Rufino-Ramos, D., Breyne, K., Di Ianni, E., Lopes, S.M., Jorge Nobre, R., Kleinstiver, B.P., Perdigão, P.R.L., Breakefield, X.O., and Pereira de Almeida, L. (2024). Exploring the potential of cell-derived vesicles for transient delivery of gene editing payloads. Adv. Drug Deliv. Rev. *211*, 115346. https://doi.org/10.1016/j.addr.2024.115346.

89. Hanlon, K.S., Meltzer, J.C., Buzhdygan, T., Cheng, M.J., Sena-Esteves, M., Bennett, R.E., Sullivan, T.P., Razmpour, R., Gong, Y., Ng, C., et al. (2019). Selection of an Efficient AAV Vector for Robust CNS Transgene Expression. Mol. Ther. Methods Clin. Dev. *15*, 320–332. https://doi.org/10.1016/j.omtm.2019.10.007.

90. Kreitz, J., Friedrich, M.J., Guru, A., Lash, B., Saito, M., Macrae, R.K., and Zhang, F. (2023). Programmable protein delivery with a bacterial contractile injection system. Nature *616*, 357–364. https://doi.org/10.1038/s41586-023-05870-7.

91. Yao, Y., Wang, J., Liu, Y., Qu, Y., Wang, K., Zhang, Y., Chang, Y., Yang, Z., Wan, J., Liu, J., et al. (2022). Variants of the adeno-associated virus serotype 9 with enhanced penetration of the blood-brain barrier in rodents and primates. Nat. Biomed. Eng. *6*, 1257–1271. https://doi.org/10.1038/s41551-022-00938-7.

92. Huang, Q., Chan, K.Y., Wu, J., Botticello-Romero, N.R., Zheng, Q., Lou, S., Keyes, C., Svanbergsson, A., Johnston, J., Mills, A., et al. (2024). An AAV capsid reprogrammed to bind human transferrin receptor mediates brain-wide gene delivery. Science *384*, 1220–1227. https://doi.org/10.1126/science.adm8386.

93. Zhang, Q., Zhang, J., Tian, Y., Zhu, G., Liu, S., and Liu, F. (2020). Efficacy of a novel double-controlled oncolytic adenovirus driven by the Ki67 core promoter and armed with IL-15 against glioblastoma cells. Cell Biosci. *10*, 124. https://doi.org/10.1186/s13578-020-00485-1.

94. Stanton, A.C., Lagerborg, K.A., Tellez, L., Krunnfusz, A., King, E.M., Ye, S., Solomon, I.H., Tabebordbar, M., and Sabeti, P.C. (2023). Systemic administration of novel engineered AAV capsids facilitates enhanced transgene expression in the macaque CNS. Med *4*, 31–50.e8. https://doi.org/10.1016/j.medj.2022.11.002.

95. Slyk, Z., Wrzesien, R., Barszcz, S., Gawrychowski, K., and Malecki, M. (2024). Adeno-associated virus vector hydrogel formulations for brain cancer gene therapy applications. Biomed. Pharmacother. *170*, 116061. https://doi.org/10.1016/j.biopha.2023.116061.

96. Rhim, J., Baek, W., Seo, Y., and Kim, J.H. (2022). From Molecular Mechanisms to Therapeutics: Understanding MicroRNA-21 in Cancer. Cells *11*, 2791. https://doi.org/10.3390/cells11182791.

97. McCarty, N.S., Graham, A.E., Studená, L., and Ledesma-Amaro, R. (2020). Multiplexed CRISPR technologies for gene editing and transcriptional regulation. Nat. Commun. *11*, 1281. https://doi.org/10.1038/s41467-020-15053-x.

98. Mahinfar, P., Mansoori, B., Rostamzadeh, D., Baradaran, B., Cho, W.C., and Mansoori, B. (2022). The Role of microRNAs in Multidrug Resistance of Glioblastoma. Cancers (Basel) *14*, 3217. https://doi.org/10.3390/cancers14133217.

99. Akhtarkhavari, T., Bahrami, A.R., and Matin, M.M. (2022). Downregulation of miR-21 as a promising strategy to overcome drug resistance in cancer. Eur. J. Pharmacol. *932*, 175233. https://doi.org/10.1016/j.ejphar.2022.175233.

100. Volak, A., LeRoy, S.G., Natasan, J.S., Park, D.J., Cheah, P.S., Maus, A., Fitzpatrick, Z., Hudry, E., Pinkham, K., Gandhi, S., et al. (2018). Virus vector-mediated genetic modification of brain tumor stromal cells after intravenous delivery. J. Neurooncol. *139*, 293–305. https://doi.org/10.1007/s11060-018-2889-2.

101. Abels, E.R., Maas, S.L.N., Nieland, L., Wei, Z., Cheah, P.S., Tai, E., Kolsteeg, C.J., Dusoswa, S.A., Ting, D.T., Hickman, S., et al. (2019). Glioblastoma-Associated Microglia Reprogramming Is Mediated by Functional Transfer of Extracellular miR-21. Cell Rep. *28*, 3105–3119.e7. https://doi.org/10.1016/j.celrep.2019.08.036.

102. Clement, K., Rees, H., Canver, M.C., Gehrke, J.M., Farouni, R., Hsu, J.Y., Cole, M.A., Liu, D.R., Joung, J.K., Bauer, D.E., and Pinello, L. (2019). CRISPResso2 provides accurate and rapid genome editing sequence analysis. Nat. Biotechnol. *37*, 224–226. https://doi.org/10.1038/s41587-019-0032-3.

103. Edgar, R., Domrachev, M., and Lash, A.E. (2002). Gene Expression Omnibus: NCBI gene expression and hybridization array data repository. Nucleic Acids Res. *30*, 207–210. https://doi.org/10.1093/nar/30.1.207.

YMTHE, Volume *33*

Supplemental Information

CRISPR targeting of mmu-miR-21a through a single adeno-associated virus vector prolongs survival of glioblastoma-bearing mice

Lisa Nieland, Anne B. Vrijmoet, Isabelle W. Jetten, David Rufino-Ramos, Alexandra J.E.M. de Reus, Koen Breyne, Benjamin P. Kleinstiver, Casey A. Maguire, Marike L.D. Broekman, Xandra O. Breakefield, and Erik R. Abels

SUPPLEMENTAL MATERIAL

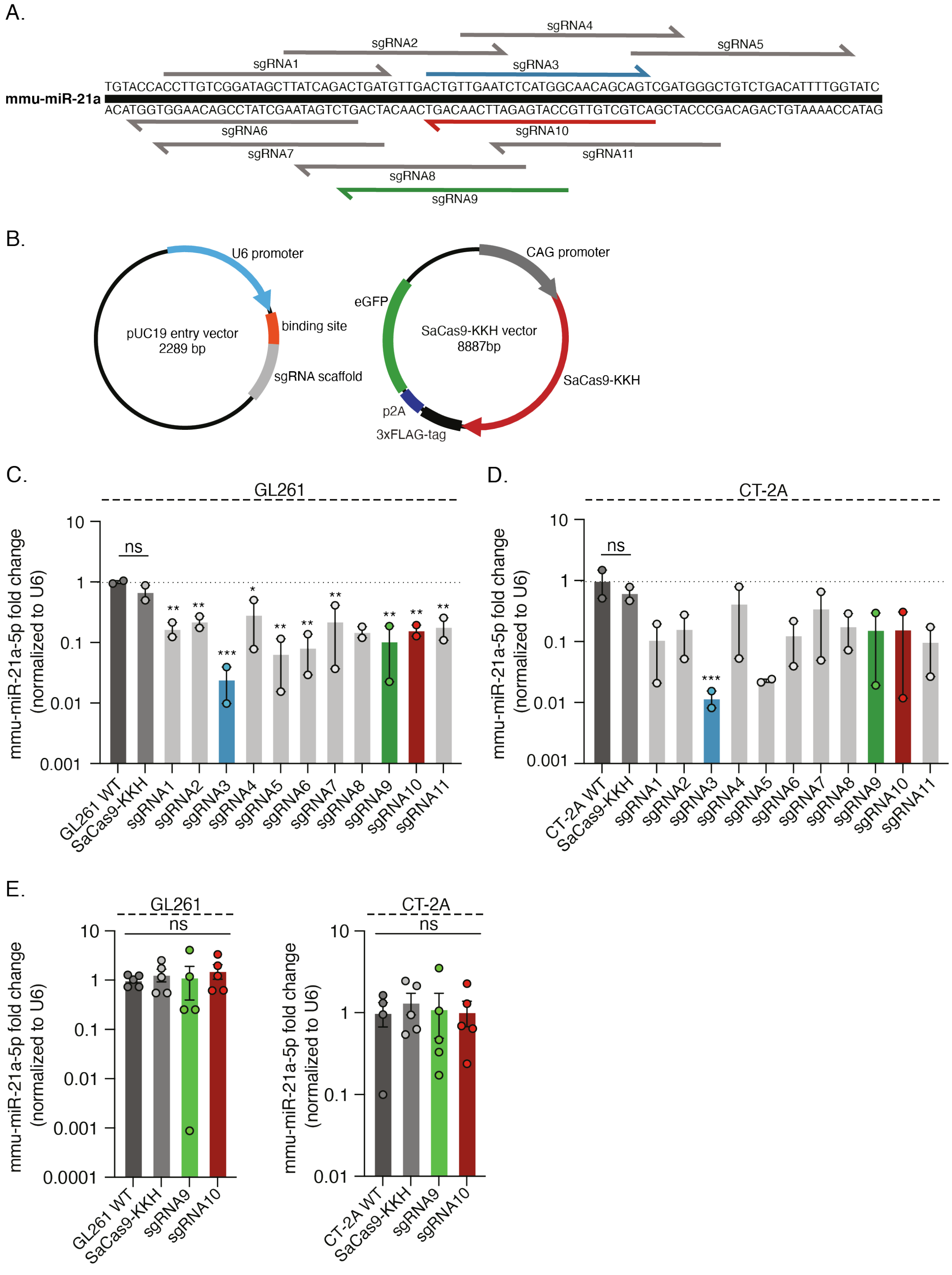

Figure S1. Screening of eleven guides targeting mouse mmu-mir-21a locus. (A) Schematic display of the mmu-mir-21a locus showing the locations of 11 guides at the mmu-miR-21a locus

at different locations showing sgRNA3 highlighted in blue, sgRNA9 in green, and sgRNA10 in red. (B) Plasmid pUC19 (left; size: 2289bp) schematically shows the U6 promoter (blue), binding site (orange), and sgRNA scaffold (grey). Plasmid MSP2588 (right; size: 8887bp) containing the SaCas9-KKH (red) under a CAG (grey) promoter and tagged by both 3xFLAG-tag (black), a peptide 2A (p2A) sequence (blue) and a green fluorescent protein (GFP - green). (C-D) Fold expression levels of mmu-miRa-21-5p targeted by 11 different guides showing 200-fold and 100-fold reduction for sgRNA3 (blue) in both GL261 and CT-2A respectively. And ~10-fold reduction for sgRNA9 (green) and sgRNA10 (red) in both GL261 and CT-2A. Cells co-transfected with SaCas9-KKH expressing plasmid and a single guide were compared to WT representing non-transfected control cell line or the SaCas9-KKH control, referring to cells single transfected with the SaCas9-KKH expressing plasmid. Data represent duplicates and are presented as the mean with SEM (error bars). Multi-comparison (Tukey) one-way ANOVA, **$p < 0.01$, ***$p < 0.001$, (ns = not significant). (E) Cell lines (GL261 and CT-2A) co-transfected with sgRNA9 (green) and sgRNA10 (red) did not show a significant change in mmu-miR-21a-5p fold expression compared to WT representing non-transfected control cell line or SaCas9-KKH control, referring to cells single transfected with the or SaCas9-KKH expressing plasmid. Data are presented as the mean with SEM (error bars). Multi-comparison (Tukey) one-way ANOVA, (ns = not significant)

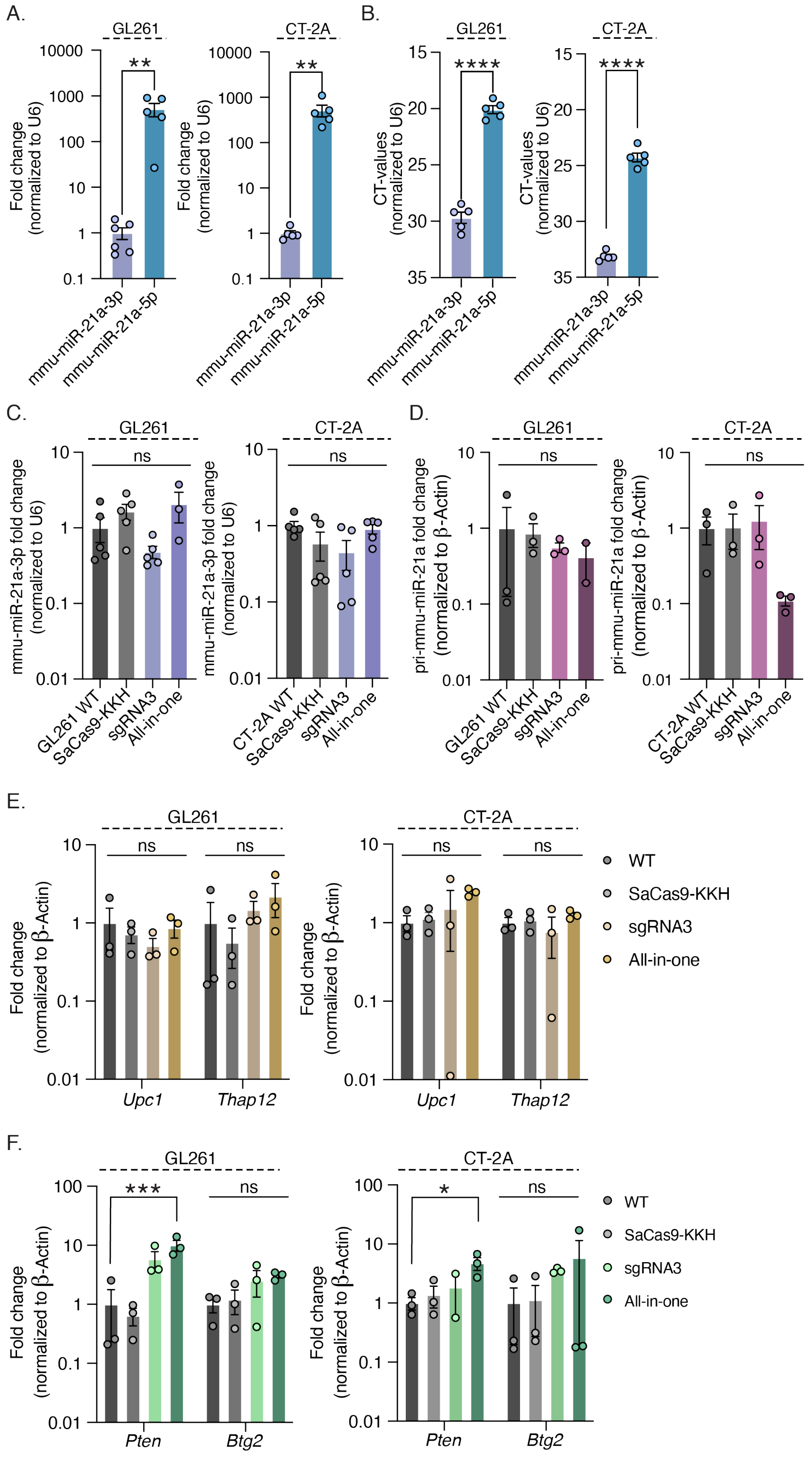
A.
GL261
CT-2A
Fold change (normalized to U6)
mmu-miR-21a-3p
mmu-miR-21a-5p
B.
CT-values (normalized to U6)
C.
mmu-miR-21a-3p fold change (normalized to U6)
GL261 WT
CT-2A WT
SaCas9-KKH
sgRNA3
All-in-one
D.
pri-mmu-miR-21a fold change (normalized to β-Actin)
E.
Fold change (normalized to β-Actin)
Upc1
Thap12
WT
SaCas9-KKH
sgRNA3
All-in-one
F.
Pten
Btg2

Figure S2. Mmu-miR-21a-3p and mmu-miR-21-5p arm analysis. (A) The mmu-miR-21a-5p arm is 516- and 520-fold upregulated compared to the mmu-miR-21a-3p arm in both GL261 and CT-2A wild-type (WT) cells respectively as normalized to the U6 housekeeping gene. Data are presented as the mean with SEM (error bars). Multi-comparison (Tukey) one-way ANOVA, ****p < 0.0001. (B) CT-values normalized to the U6 housekeeping show low expression levels of mmu-miR-21a-3p (~35 CT) compared to mmu-miR-21a-5p (~20 CT) in WT GL261 and CT-2A cell lines. Data are presented as the mean with SEM (error bars). Multi-comparison (Tukey) one-way ANOVA, ****p < 0.0001. (C) Fold expression of mmu-miR-21a-3p showed no differences between edited (sgRNA3 and all-in-one) compared to non-edited (WT and SaCas9-KKH) controls for both cell lines (GL261 and CT-2A). Data are presented as the mean with SEM (error bars). Multi-comparison (Tukey) one-way ANOVA, ns – not significant. (D) Fold expression of mmu-pri-miR-21a shows no differences between edited (sgRNA3 and all-in-one) compared to non-edited (WT and SaCas9-KKH) controls for both cell lines (GL261 and CT-2A). Data are presented as the mean with SEM (error bars). Multi-comparison (Tukey) one-way ANOVA, ns – not significant. (E) Downstream mRNA target of the mmu-miR-21a-5p arm *Pten* was upregulated in all-in-one CRISPR-edited cells compared to WT cells in GL261 and CT-2A cells, respectively. Btg2 was not differently expressed between groups. Data are presented as the mean with SEM (error bars). Multi-comparison (Tukey) one-way ANOVA, *p < 0.05, ***p < 0.001, ns – not significant. (F) Downstream mRNA targets of the mmu-miR-21a-3p arm, *Upc1* and *Thap12* showed no differences in edited cells (sgRNA3 and all-in-one) compared to WT for both GL261 and CT-2A cell lines. Data are presented as the mean with SEM (error bars). Multi-comparison (Tukey) one-way ANOVA, ns – not significant.

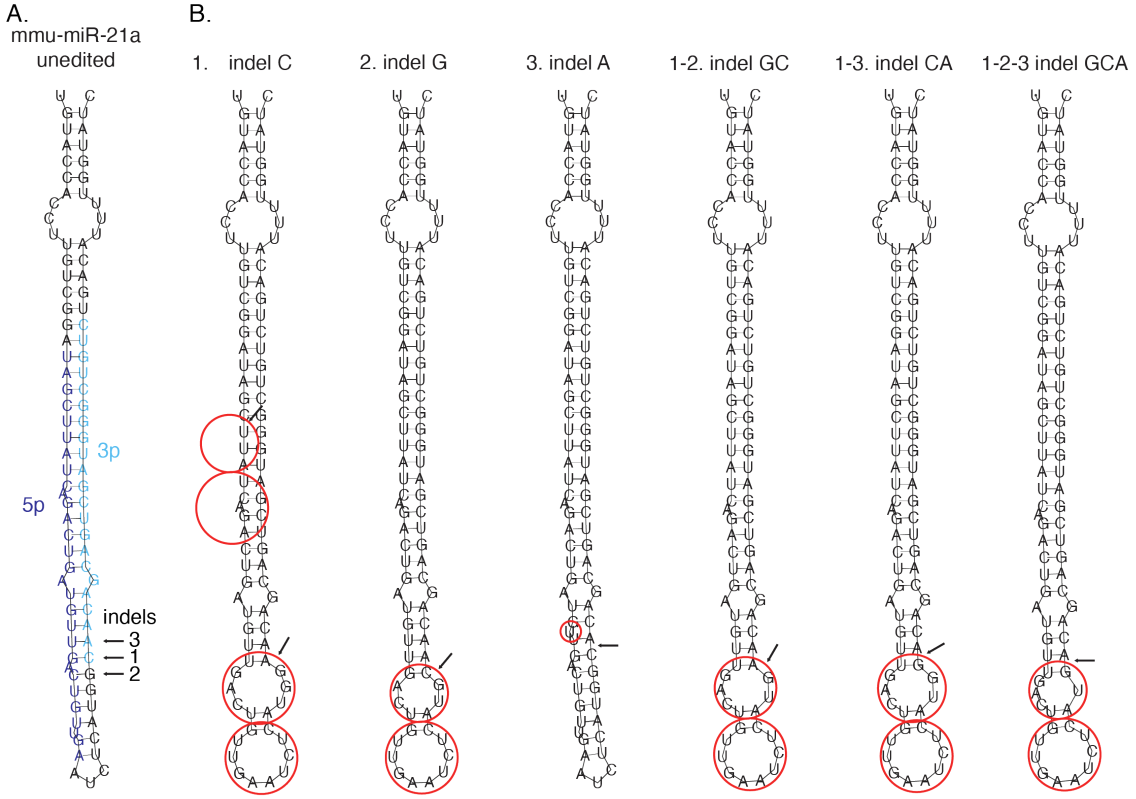

Figure S3. Predicted structures of RNA folding of the pre-mmu-miR-21a hairpin loop. (A) Schematic illustration of the non-edited pre-mmu-miR-21a hairpin loop with the -5p and -3p highlighted in blue. (B) By using RNA-fold (http://rna.tbi.univie.ac.at/cgi-bin/RNAWebSuite/RNAfold.cgi) hairpin loop predicted structures are displayed of RNA folding after deleting one of the three most frequent indels observed: 1) indel "C" (55.5%), 2) indel "G", 3) indel "T" (64.2%), or combinations; 1-2 indel "GC", 1-3 indel CA; 1-2-3 indel "GCA" (Figure 1E (shown in reverse complement)). The arrows point to the location of the deletion and in red are the altered sequences highlighted compared to the non-edited hairpin loop.

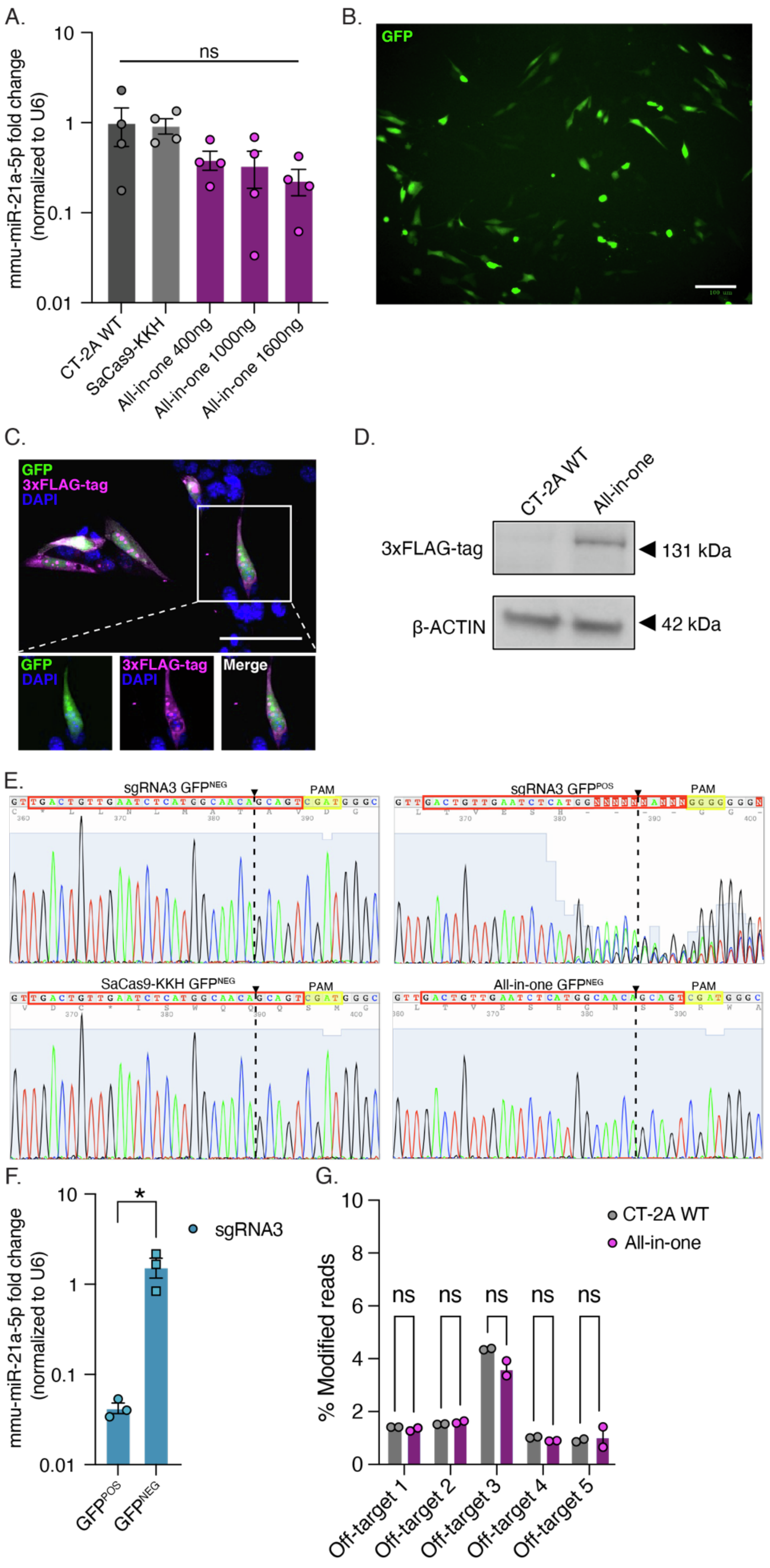
A.
mmu-miR-21a-5p fold change (normalized to U6)
10
1
0.1
0.01
ns
CT-2A WT
SaCas9-KKH
All-in-one 400ng
All-in-one 1000ng
All-in-one 1600ng
B.
GFP
C.
GFP
3xFLAG-tag
DAPI
Merge
D.
CT-2A WT
All-in-one
3xFLAG-tag
131 kDa
β-ACTIN
42 kDa
E.
sgRNA3 GFPNEG
sgRNA3 GFPPOS
SaCas9-KKH GFPNEG
All-in-one GFPNEG
PAM
F.
mmu-miR-21a-5p fold change (normalized to U6)
sgRNA3
GFPPOS
GFPNEG
G.
% Modified reads
CT-2A WT
All-in-one
ns
Off-target 1
Off-target 2
Off-target 3
Off-target 4
Off-target 5

Figure S4. Validation of all-in-one transfection components in tumor cells. (A) Fold expression of mmu-miR-21a-5p levels after transfection with three different concentrations of the all-in-one construct (400ng, 1000ng and 1600ng) targeting mmu-miR-21a-5p. Data represent triplicates and are presented as the mean with SEM (error bars). Multi-comparison one-way ANOVA, ns – not significant. (B) The representative image shows 80% of the cells positive for GFP after a single transfection with the all-in-one plasmid. (Magnification 4x; scale bar 100 μm). (C) Representative images showing GFP^{POS} cells post-transfection with the all-in-one plasmid co-localized with 3xFLAG-tag antibody which is fused to the SaCas9-KKH protein. (Magnification 40x; scale bar 10 μm). (D) Western blot analysis shows the presence of 3xFLAG-tag, GFP and SaCas9-KKH protein in GFP^{POS} sorted cells construct, normalized to β-actin. (E) Sanger sequencing plots show no disruption of the mmu-miR-21a sequence after co-transfection of glioma cells with sgRNA3 and SaCas9-KKH plasmid sorted for GFP^{NEG} but showed an indel for GFP^{POS} sorted cells. Both SaCas9-KKH only GFP^{NEG} and all-in-one GFP^{NEG} showed a non-edited mmu-miR-21a sequence. (F) qRT-PCR shows significantly reduced mmu-miR-21a-5p fold expression of CT-2A cells co-transfected with sgRNA3 and SaCas9-KKH sorted for GFP^{POS} compared to GFP^{NEG} (blue), non-transfected CT-2A WT cells and single-transfected SaCas9-KKH only (grey) GFP^{POS} and GFP^{NEG} control. Data represent triplicates and are presented as the mean with SEM (error bars). Multi-comparison one-way ANOVA, *$p<0.05$, ****$p<0.0001$. (G) NGS analysis of the top 5 saCas9-KKH predicted off-targets comparing CT-2A WT non-edited with CRISPR-edited cells of all-in-one GFP^{POS} sorted. No differences in the percentage of modified reads were observed.

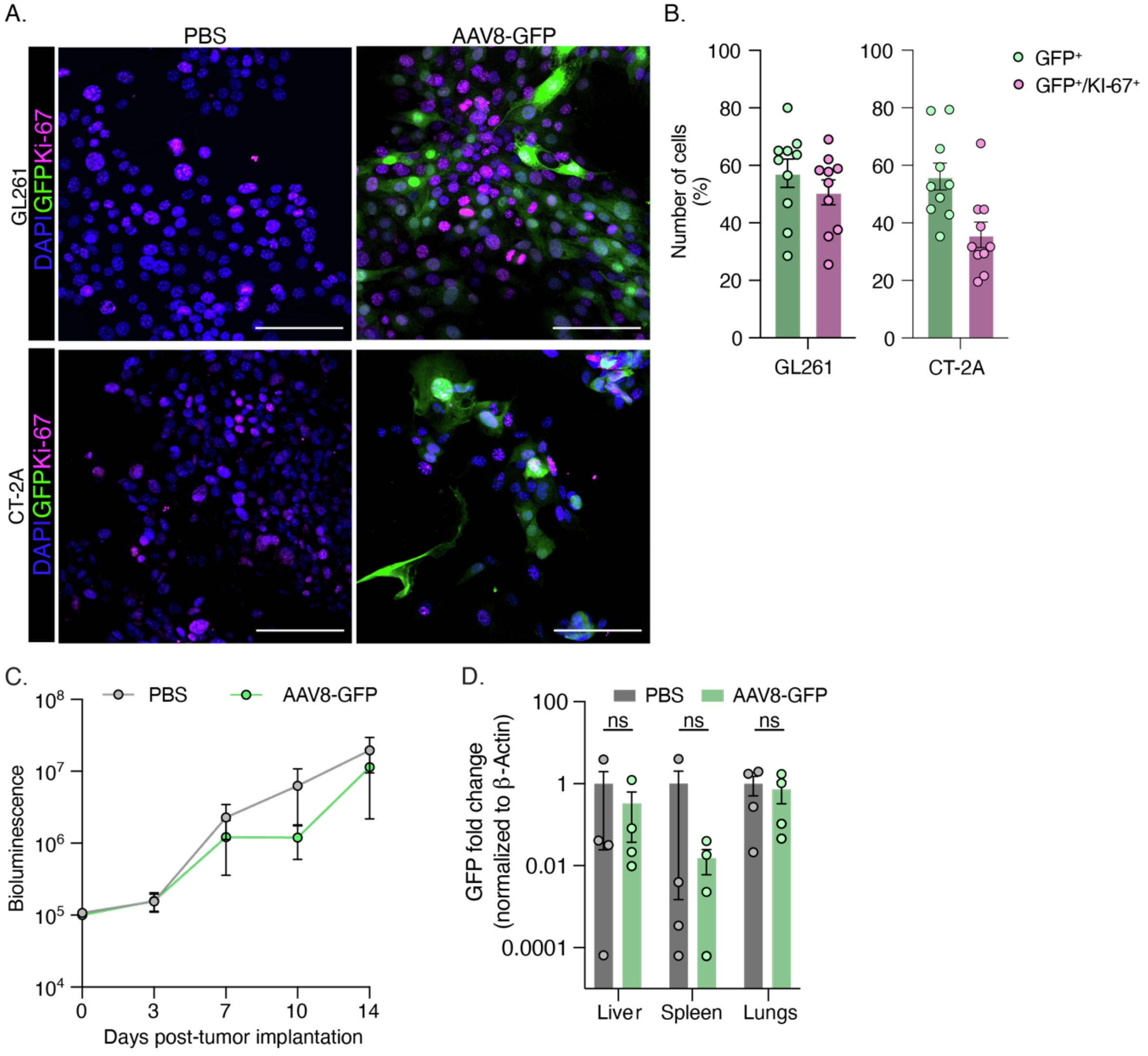

Figure S5. Transduction efficiency of AAV8 in GL261 and CT-2A in culture and orthotopic tumor grafts. (A) Representative images show the transduction profile of AAV8-GFP in glioma cell lines GL261 and CT-2A showing co-localization of GFP^{POS} cells with Ki-67 (proliferation marker) (pink) compared to PBS control. (Magnification 10x; scale bar 100 μm). (B) The number of GFP^{POS} cells as a percentage of total cells based on $DAPI^{POS}$ cells, were quantified showing a ~55% transduction rate in GL261 cells and CT-2A cells *in vitro*. Co-localization of and GFP^{POS}Ki-67^{POS} cells were quantified as a percentage of total cells ($DAPI^{POS}$) and showed a ~50% and ~35% in both cell lines, respectively. (C) Bioluminescence levels of mice implanted with CT-2A cells

and i.c. injected with AAV8-GFP (green) or PBS (grey) at day 7 post-tumor injection showing tumor growth, as measured by IVIS and over 14 days, did not significantly differ between mice injected with PBS (n=6) or AAV8-GFP (n=6). Data are presented as the mean with SEM (error bars). Using Šídák’s multiple comparisons two-way ANOVA, showed no significant differences between groups. (D) qRT-PCR measuring GFP levels in the lungs and spleen showed no significant differences between AAV8-GFP (green) and PBS control (grey). In the liver, significant higher expression of GFP was observed in AAV8-GFP. Data are presented as four replicates and show the mean with SEM (error bars). Šídák's multiple comparisons two-way ANOVA, no significant differences between groups were observed.

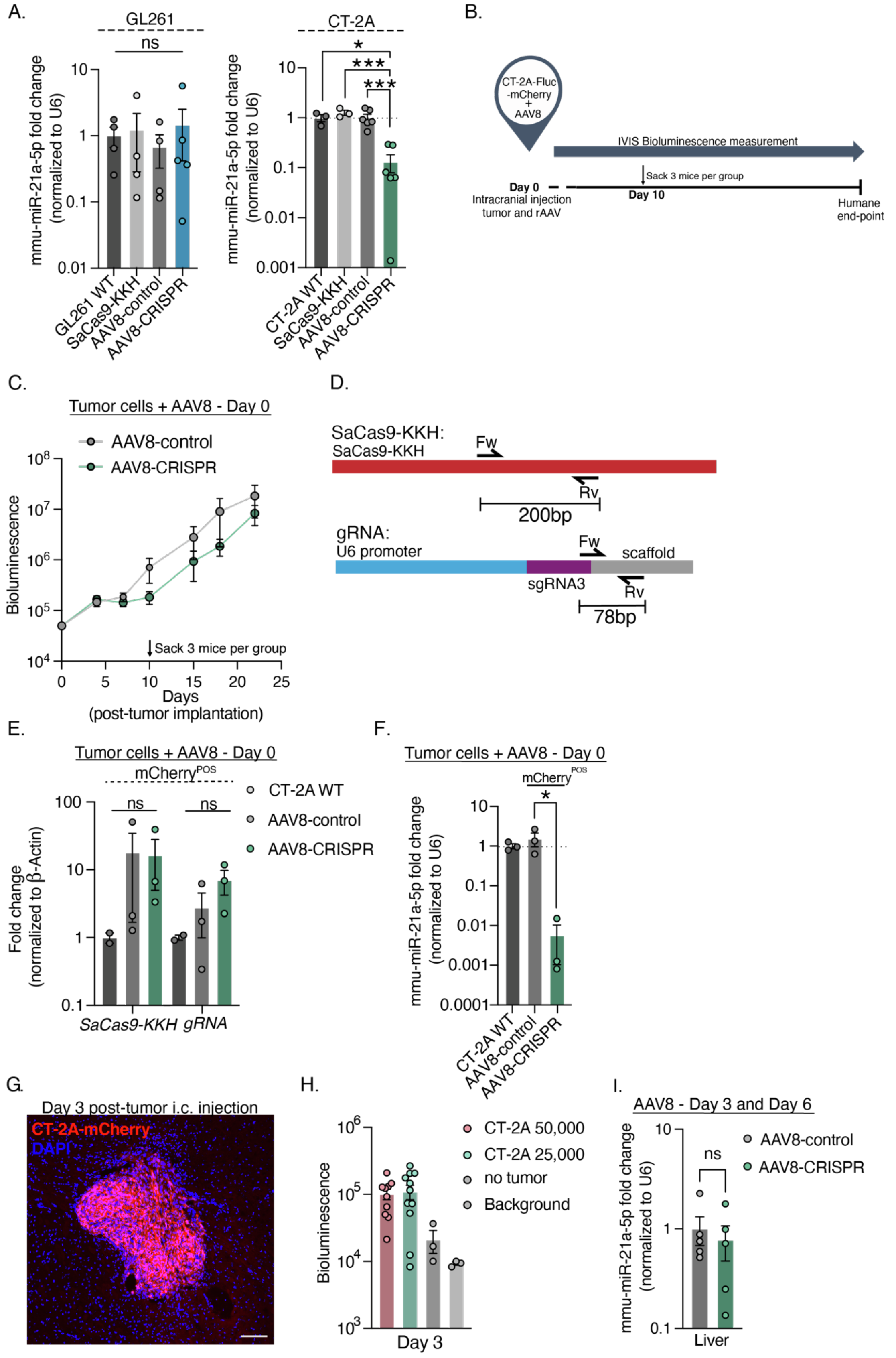

A.
GL261
ns
mmu-miR-21a-5p fold change (normalized to U6)
GL261 WT
SaCas9-KKH
AAV8-control
AAV8-CRISPR
CT-2A
*
***
***
CT-2A WT
B.
CT-2A-Fluc -mCherry + AAV8
IVIS Bioluminescence measurement
Sack 3 mice per group
Day 0
Intracranial injection tumor and rAAV
Day 10
Humane end-point
C.
Tumor cells + AAV8 - Day 0
AAV8-control
AAV8-CRISPR
Bioluminescence
Sack 3 mice per group
Days (post-tumor implantation)
D.
SaCas9-KKH:
SaCas9-KKH
Fw
Rv
200bp
gRNA:
U6 promoter
scaffold
sgRNA3
78bp
E.
Tumor cells + AAV8 - Day 0
mCherry POS
ns
CT-2A WT
AAV8-control
AAV8-CRISPR
Fold change (normalized to β-Actin)
SaCas9-KKH gRNA
F.
Tumor cells + AAV8 - Day 0
mCherry POS
*
G.
Day 3 post-tumor i.c. injection
CT-2A-mCherry
DAPI
H.
CT-2A 50,000
CT-2A 25,000
no tumor
Background
Day 3
I.
AAV8 - Day 3 and Day 6
Liver

Figure S6. Testing of AAV8-CRISPR *in vitro* and in tumor-bearing mice. (A) *In vitro* transfection with the AAV8-CRISPR and AAV8-control vectors pre-packaging in CT-2A (left) and GL261 (right) glioma cells. mmu-miR-21a-5p expression was significantly decreased in CT-2A cells transfected with 400ng pre-packaged AAV8-CRISPR plasmid compared to CT-2A WT, SaCas9-KKH, and AAV8-control. Mmu-miR-21a-5p expression was reduced in GL261 cells transfected with pre-packaged AAV8-CRISPR compared to GL261 WT, SaCas9-KKH, and AAV8-Control, observed differences were not significant. Multi-comparison (Tukey) one-way ANOVA, $*p < 0.05$. (B) Schematic illustration of the experimental setup to deliver AAV8-CRISPR i.c. simultaneously with the tumor implantation. At day 0, mice were i.c. injected with 5×10^4 CT-2A cells resuspended in AAV8-CRISPR or AAV8-control (2×10^{10} gc/μL). Three mice per group were sacrificed for pathology purposes at day 10 post i.c. injections. (C) Bioluminescence signal was measured by IVIS, comparing mice i.c. injected with AAV8-CRISPR (n=8) or AAV8-control (n=8). Data are presented as the mean with SEM (error bars). Šídák's multiple comparisons two-way ANOVA showed no significant differences between groups. (D) Schematic display of a primer set flanking the SaCas9-KKH with an amplicon of 200bp and a primer set to amplify the gRNA with an amplicon of 78bp. (E) Mice implanted with CT-2A-FLuc-mCherry tumor cells and AAV (AAV8-CRISPR and AAV8-control) simultaneously. Brains were FACS-sorted for mCherry^{POS} cells 10 days post-i.c. injection. Fold expression of SaCas9-KKH and guide (sgRNA3) were measured by qRT-PCR. No significant differences were observed for SaCas9-KKH and gRNA expression levels in mCherry^{POS} tumor cells transduced by AAV8-CRISPR and AAV8-SaCas9-Control compared to CT-2A WT. Data represent triplicates (n=3 mice) and are presented as the mean with SEM (error bars). Multi-comparison (Tukey) one-way ANOVA, (ns = not significant). (F) Mmu-miR-21a-5p expression analysis of FACS sorted

$mCherry^{POS}$ tumor cells showed a 180-fold reduction in tumor-bearing mice injected with AAV8-CRISPR compared to AAV8-control and to CT-2A WT cells. Data represents triplicates (n=3 mice) and are presented as the mean with SEM (error bars). Multiple-comparison (Turkey) one-way ANOVA, *p<0.05 (G) Tumor engraftment was validated by mCherry fluorescent cells visualized in the left striatum 3 days post-implantation of CT-2A-FLuc-mCherry cells. (Magnification 4x; scale bar 100 μm). (H) Mice that got injected with 25,000 (n=12) or 50,000 (n=12) CT-2A-FLuc-mCherry cells showed positive bioluminescence signal at day 3 post-tumor implantation prior to AAV8 administration compared to mice without tumor cells implanted and to the IVIS background signal. Data are presented as the mean with SEM (error bars), Multi-comparison (Tukey) one-way ANOVA, (ns = not significant). (I) Fold expression of mmu-miR-21a-5p was measured after 16 days in the livers of mice that got treated with AAV8 at day 3 and day 6 post-tumor implantation and showed no significant difference between AAV8-control (n=5) and AAV8-CRISPR treated mice (n=5). Data represent five replicates and are presented as the mean with SEM (error bars). Unpaired t test, (ns = not significant).

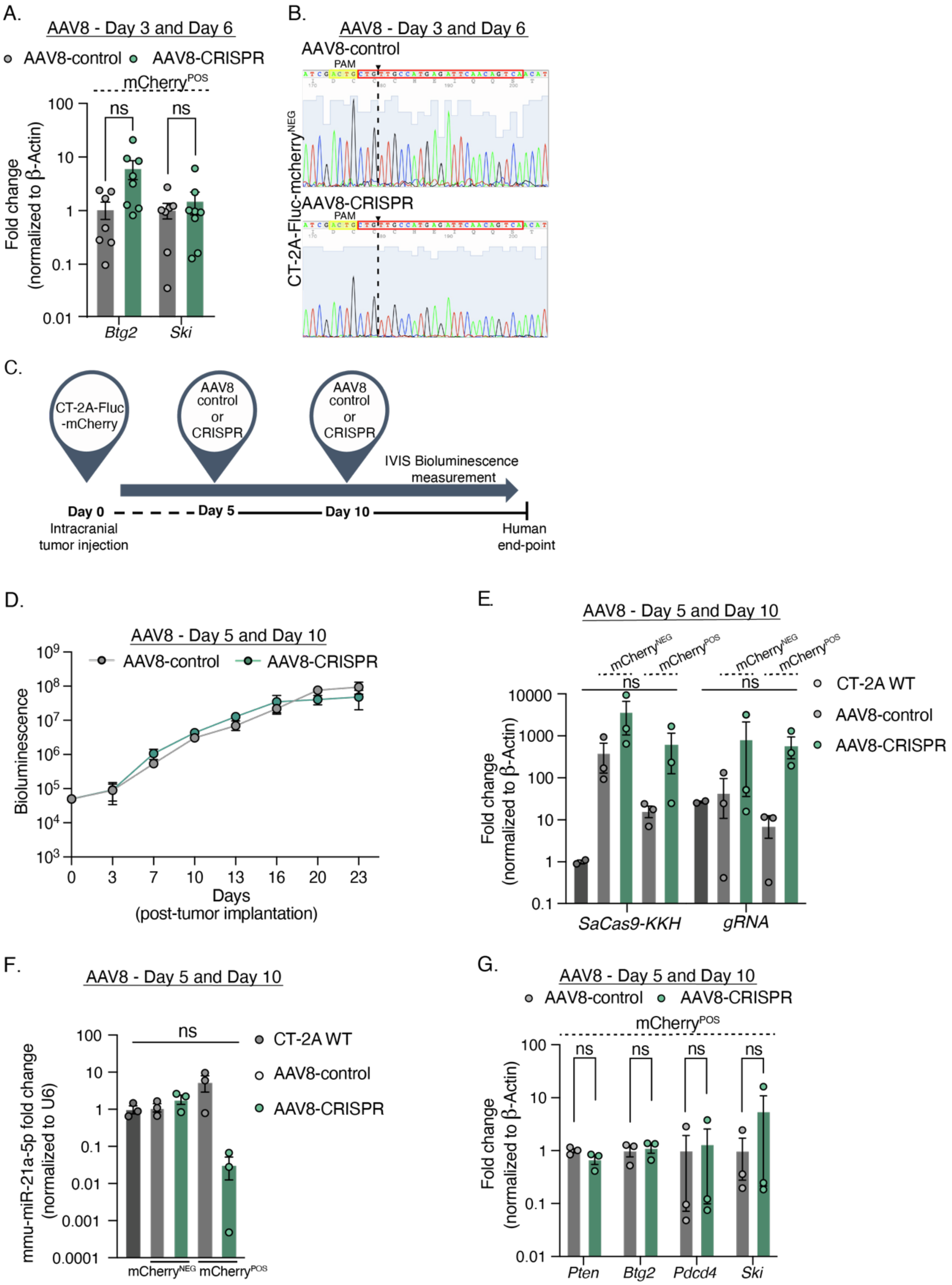
A.
AAV8 - Day 3 and Day 6
AAV8-control
AAV8-CRISPR
mCherryPOS
ns
ns
Fold change (normalized to β-Actin)
Btg2
Ski
B.
AAV8 - Day 3 and Day 6
AAV8-control
PAM
AAV8-CRISPR
CT-2A-Fluc-mcherryNEG
C.
CT-2A-Fluc -mCherry
AAV8 control or CRISPR
AAV8 control or CRISPR
IVIS Bioluminescence measurement
Day 0
Day 5
Day 10
Intracranial tumor injection
Human end-point
D.
AAV8 - Day 5 and Day 10
AAV8-control
AAV8-CRISPR
Bioluminescence
Days (post-tumor implantation)
E.
AAV8 - Day 5 and Day 10
mCherryNEG
mCherryPOS
ns
CT-2A WT
AAV8-control
AAV8-CRISPR
Fold change (normalized to β-Actin)
SaCas9-KKH
gRNA
F.
AAV8 - Day 5 and Day 10
ns
CT-2A WT
AAV8-control
AAV8-CRISPR
mmu-miR-21a-5p fold change (normalized to U6)
mCherryNEG
mCherryPOS
G.
AAV8 - Day 5 and Day 10
AAV8-control
AAV8-CRISPR
mCherryPOS
ns
Fold change (normalized to β-Actin)
Pten
Btg2
Pdcd4
Ski

Figure S7. Timing of AAV treatment is important in tumor reduction. (A) Fold expression of mmu-miR-21a-5p downstream target genes (*Btg2*, *Ski*) were analyzed at day 16 post-tumor implantation, and no significant differences were observed in cells of mice treated with AAV8-CRISPR (n=8) or AAV8-control (n=8) at day 3 and day 6 post-tumor implantation for mCherry^{POS} (tumor) cells. Data are presented as the mean with SEM (error bars). Unpaired t-test, (ns = not significant). (B) Representative panels of sanger sequencing of PCR products spanning mmu-miR-21a using 4Peaks software shows position of the sgRNA3 (red) and the nicking site (black dashed line) in the mmu-miR-21a reverse sequence traces in mCherry^{NEG} cells of tumor-bearing mice i.c. injected with AAV8-CRISPR compared to the AAV8-control at day 3 and day 6 post-tumor implantation. Blue shading behind the peaks represents base quality. No indels were detected in the mmu-miR-21a sequence of both AAV-CRISPR and AAV8-control. (C) Schematic layout of the experimental design treating mice at day 5 and day 10 with AAV8-CRISPR or AAV8-control post-tumor implantation. Mice were sacrificed for pathology purposes at day 15 post i.c. injections. (D) Across all time points, bioluminescence signal (as measured by IVIS) did not significantly differ between mice injected with AAV8-CRISPR (n=11) or AAV8-control (n=11) at day 5 and day 10 post-tumor implantation. Data are presented as the mean with SEM (error bars). Šídák's multiple comparisons two-way ANOVA showed no significant differences between groups. (E) Fold expression of SaCas9-KKH and gRNA of mice treated at day 5 and day 10 with AAV8-CRISPR or AAV8-control post-tumor injection and FACS-sorted for mCherry^{POS} tumor cells and mCherry^{NEG} non-tumor cells at day 15 post-tumor implantation were measured by qRT-PCR and showed no significant differences between groups. Fold expression of gRNA levels were not significantly different between groups. Data represent triplicates (n=3 mice) and are presented as the mean with SEM (error bars). Multi-comparison (Tukey) one-way ANOVA. (F) Mmu-miR-

21a-5p expression analysis of FACS sorted mCherry^{POS} (tumor) cells and mCherry^{NEG} (non-tumor) cells in tumor-bearing mice administered with AAV8-CRISPR or AAV8-control showed no significant differences. Data represent triplicates and are presented as the mean with SEM (error bars). Multi-comparison one-way ANOVA, (ns = not significant). (G) Fold expression analysis of downstream mmu-miR-21a-5p mRNA targets (*Pten, Btg2, Pdcd4* and *Ski*) were analyzed at day 15 post-tumor implantation of mice administered with AAV8-CRISPR (n=3) or AAV8-control (n=3) at day 5 and day 10 post-tumor implantation for mCherry^{POS} (tumor) cells. Data and are presented as the mean with SEM (error bars). Unpaired t-test. (ns = not significant).

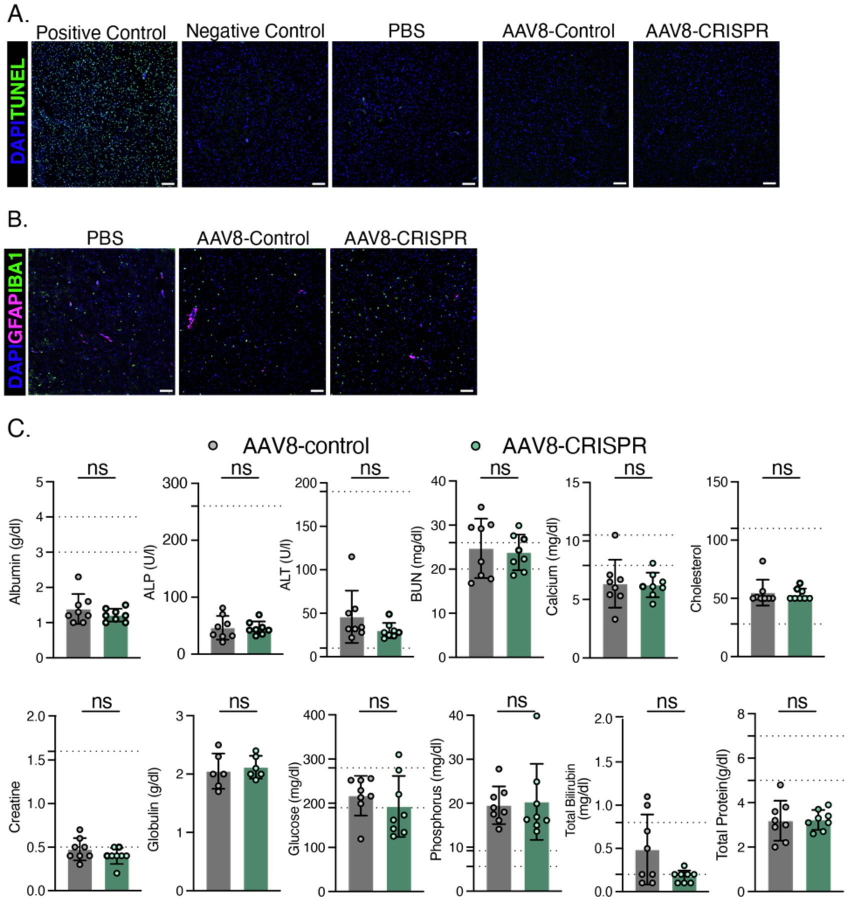

Figure S8. Local toxicity and blood toxicology following AAV injection. (A) AAV8-induced local toxicity was determined by terminal deoxynucleotidyl transferase dUTP nick end labeling (TUNEL) staining of brains from mice injected at day 7 with PBS, AAV8-control or AA8-CRISPR and sacrificed at day 14. The positive control, tissue incubated with DNAse I, showed DNA breaks in green in each cell stained for DAPI, no DNA breaks were observed in the negative control and PBS conditions, or brain sections of mice treated with AAV8-control or AA8-CRISPR. (B) Local toxicity post-AAV8-control or -AAV8-CRISPR injection compared to PBS was analyzed in brains stained for GFAP and IBA1. No increase in gliosis or microglia activation was observed. (C) Blood pathology was analyzed to determine AAV-induced toxicology after mice were treated with AAV-CRISPR or AAV8-control, no significant elevated levels of multiple blood

markers (Albumin; ALP; ALT; BUN; Calcium; Cholesterol; Creatine; Globulin; Glucose; Phosphorus; Total Bilirubin; Total Protein) were detected for both groups. Data represent two independent experiments (n=8 mice) and are presented as the mean with SEM (error bars). Multi-comparison one-way ANOVA, (ns = not significant).

SUPPLEMENTAL TABLES

Table S1. Overview of the top 5 predicted off-targets as determined by Cas-OFFinder.

Off-target	DNA	Location	Position	Direction	Mismatches
Off-target 1	ACTGTgGcATCTCATGaCAACAGAAAT	Chr4	153280201	+	3
Off-target 2	ACTGTTaAAcCTCATGGCAAtTATAGT	Chr11	112908309	-	3
Off-target 3	ACTGTTGAcTCTCcTGGCAAtCACGGT	Chr11	119227215	-	3
Off-target 4	tCTtTTGAAgCTCATGGCAACTTAAAT	Chr10	36314200	-	3
Off-target 5	AaTGTTGAtgaTCATGGCAACGTAAAT	Chr15	45517500	-	4

Table S2. Primers sets amplifying the off-targets.

Off-targets		Primer sequence (5'→3')	Amplicon size (bp)	Annealing temperature
Off-target 1	FW	CACTGCCCCAGCTGTAAGAT	248	60C
	RV	CCCTTCATCCACTTTGGCTT		
Off-target 2	FW	TAGAAAAGGTCGGCGCTGG	243	60C
	RV	CTCTTGAAGTATGGCCGTGGT		
Off-target 3	FW	CCCACACATGGTGATCCGA	247	60C
	RV	GGCCGGCTAAGATTCACCC		
Off-target 4	FW	ACAGCCAGGAAATATAGGATCATTC	236	60C
	RV	TTTTGGTGCAGCAGTTGGGT		
Off-target 5	FW	GTGGGGCTCAGATGCAGTAT	227	60C
	RV	TGACTAGGACACACAGCAAATGT		

Table S3. sgRNA sequences.

Name	Sequence	PAM
sgRNA1	GCCTTGTCGGATAGCTTATCAG	NNTGAT
sgRNA2	GTTATCAGACTGATGTTGACTG	NNGAAT
sgRNA3	GACTGTTGAATCTCATGGCAAC	NNCAGT
sgRNA4	GTTGAATCTCATGGCAACAGCA	NNCGAT
sgRNA5	GAGTCGATGGGCTGTCTGACAT	NNTGGT
sgRNA6	GGTCTGATAAGCTATCCGACAA	NNTGGT
sgRNA7	GTCAGTCTGATAAGCTATCCGA	NNAGGT
sgRNA8	GAGATTCAACAGTCAACATCAG	NNTGAT
sgRNA9	GCCATGAGATTCAACAGTCAAC	NNCAGT
sgRNA10	GACTGCTGTTGCCATGAGATTC	NNCAGT
sgRNA11	GGCCCATCGACTGCTGTTGCCA	NNAGAT

Table S4. Overview of the primer sets used to amplify mmu-miR-21a sequence to analyze for Sanger sequencing and NGS.

Primer ID		Primer sequence (5'→3')	Amplicon size (bp)	Annealing temperature
**Sanger sequencing**				
mmu-miR-21a	FW	GGTTCACCTAGAGTGGGAATCT	625	60C
mmu-miR-21a	RV	ATTGGGGTAGTCGTCACAGTC		
**NGS sequencing**				
mmu-miR-21a	FW	TTGACTGCAAACCATGATGCT	222	67C
mmu-miR-21a	RV	TGCTTTAAACCCTGCCTGAGC		
oS280 pUC19		CAGGGTTATTGTCTCATGAGCGG		

Table S5. TaqMan primers

Primers	Assay ID	Manufacturer
U6	001973	Thermo Fisher
Mmu-miR-21a-5p	000397	Thermo Fisher
Mmu-miR-21a-3p	002493	Thermo Fisher
pri-mmu-miR-21a	Mm03306822_pri	Thermo Fisher

Table S6. qRT-PCR Primer sequences

Primer ID	Primer sequence (5’→3’)
SaCas9-KKH forward primer	AGAAGATGATTAACGAAATGCAGAAGCG
SaCas9-KKH reverse primer	TCGTAATTGAATGGATTATTCAGCAGATCTTC
gRNA forward primer	CTGTTGAATCTCATGGCAACGTTTTAGTAC
gRNA reverse primer	GAGATAAACACGGCATTTTGCCTT
GFP forward primer	CAAGGGCGAGGAGCTGTT
GFP reverse primer	TCGTCCATGCCGAGAGTGAT
Pten forward primer	TGGATTCGACTTAGACTTGACCT
Pten reverse primer	GCGGTGTCATAATGTCTCTCAG
Btg2 forward primer	TGAGCCACGGGAAGAGAAC
Btg2 reverse primer	GCCCTACTGAAAACCTTGAGTC
Pdcd4 forward primer	AAGACGACTGCGGAAAAATTCA
Pdcd4 reverse primer	CTTCTAACCGCTTCACTTCCATT
Ski forward primer	CAAAACAGACGACACTTCCTCA
Ski reverse primer	CAGCCGAGGCTCTTATTGGAG
Ucp1 forward primer	GCTTTGCCTCACTCAGGATTGG
Ucp1 reverse primer	CCAATGAACACTGCCACACCTC
Thap12 forward primer	TGAACAACCCGCATAGCAGACAC
Thap12 reverse primer	CCTGCTCTTGTTCAGAAGTCTCC
